# Thiazolidinedione-Conjugated Lupeol Derivatives as Potent Anticancer Agents Through a Mitochondria-Mediated Apoptotic Pathway

**DOI:** 10.3390/molecules29204957

**Published:** 2024-10-20

**Authors:** Siqi Deng, Yinxu Zhao, Xiaoshan Guo, Xian Hong, Gang Li, Yuchun Wang, Qingyi Li, Ming Bu, Ming Wang

**Affiliations:** 1 College of Pharmacy, Qiqihar Medical University, Qiqihar 161006, China; 17735745701@163.com (S.D.); zyx99813@163.com (Y.Z.); xiaoshan990519@163.com (X.G.); wyc58811@163.com (Y.W.); qingyili2022@163.com (Q.L.); 2 Research Institute of Medicine & Pharmacy, Qiqihar Medical University, Qiqihar 161006, China; hongxian24@163.com (X.H.); lgyjy@qmu.edu.cn (G.L.)

**Keywords:** triterpenoid, lupeol, thiazolidinedione, hybrids, antitumor, apoptotic

## Abstract

To improve the potential of lupeol against cancer cells, a privileged structure, thiazolidinedione, was introduced into its C-3 hydroxy group with ester, piperazine-carbamate, or ethylenediamine as a linker, and three series of thiazolidinedione-conjugated compounds (**6a**–**i**, **9a**–**i**, and **12a**–**i**) were prepared. The target compounds were evaluated for their cytotoxic activities against human lung cancer A549, human breast cancer MCF-7, human hepatocarcinoma HepG2, and human hepatic LO2 cell lines, and the results revealed that most of the compounds displayed improved potency over lupeol. Compound **12i** exhibited significant activity against the HepG2 cell line, with an IC_50_ value of 4.40 μM, which is 9.9-fold more potent than lupeol (IC_50_ = 43.62 μM). Mechanistic studies suggested that **12i** could induce HepG2 cell apoptosis, as evidenced by AO/EB staining and annexin V-FITC/propidium iodide dual staining assays. Western blot analysis suggested that compound **12i** can upregulate Bax expression, downregulate Bcl-2 expression, and activate the mitochondria-mediated apoptotic pathway. Collectively, compound **12i** is worthy of further investigation to support the discovery of effective agents against cancer.

## 1. Introduction

Cancer is the leading cause of death and has a serious impact on human health worldwide [1,2]. Chemotherapy is a traditional method for treating cancer [3]. However, despite the effectiveness of chemotherapeutic drugs, they can also cause adverse reactions in normal cells and are associated with multidrug resistance (MDR) [4]. Therefore, it is urgent to develop more effective drugs with low toxicity. Natural products have a long history of medicinal use and are one of the main sources of new antitumor drugs [5,6]. At present, many plant-based antitumor drugs are in clinical use, such as camptothecin [7], paclitaxel [8], and podophyllotoxin [9].

**Figure 1:**
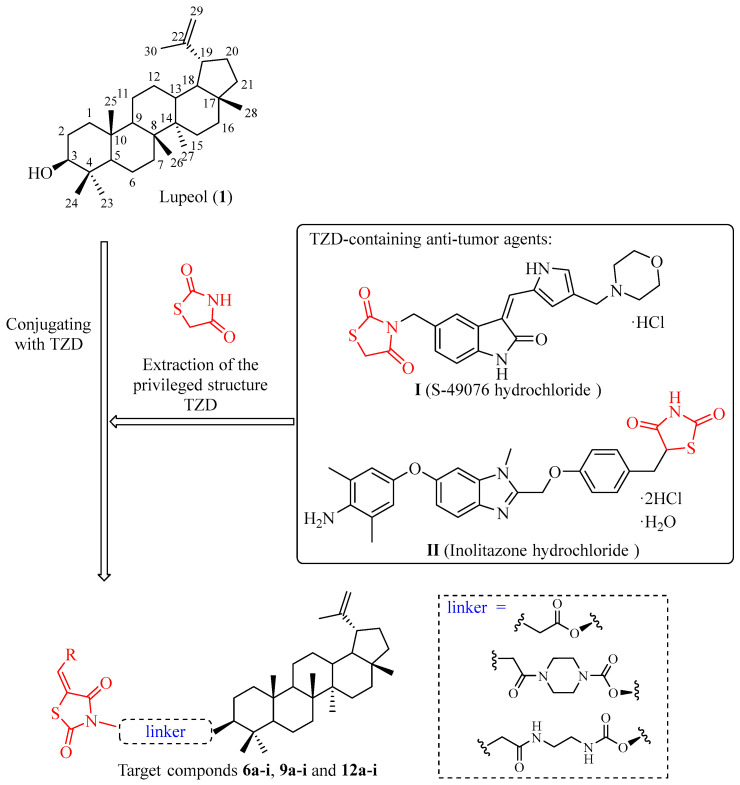
Structures of lupeol and the design of TZD-conjugated lupeol derivatives.

Lupeol (**1**) (Figure 1) is a triterpenoid widely found in fruits and vegetables, such as grapes, white cabbage, green pepper, strawberry, and others [10,11]. It has numerous biological activities, including those against arthritis, inflammation, diabetes, cancer, renal toxicity, heart diseases, and hepatic toxicity, among others [12,13,14,15,16,17]. Of these, its antitumor activity has received the most attention, and many reports in the literature have discussed the antitumor effects of lupeol. T.R. Min et al. reported that lupeol can induce cancer cell apoptosis by suppressing EGFR/STAT3 activity [18]. Bhattacharyya et al. reported that lupeol showed antitumor activity by inhibiting angiogenesis in a mouse model of melanoma [19]. Zhang X. et al. revealed that lupeol can induce autophagy by inhibiting the Akt-mTOR pathway and activating an autophagy-inhibited epithelial–mesenchymal transition (EMT) [20]. Homa Fatma et al. reported that lupeol can inhibit cancer cell proliferation by regulating the PI3K/AkT/mTOR and RAS/BRAF/MEK/ERK pathways, inducing apoptosis in cancer cells [21]. Nigam et al. showed that topical lupeol applications (200 μg/mouse) can prevent 7,12-dimethylbenz(a)anthracene (DMBA)-induced DNA damage (DNA strand breaks) in murine skin [22].

Because of lupeol’s moderate antitumor activity and low water solubility, medicinal chemists have carried out structural modification studies based on it, resulting in many structurally novel lupeol derivatives. For example, lupeol-3-succinate induced autophagy in tumor cells, and lupeol carbamate–quaternary ammonium salt derivatives have an anti-proliferative activity that is tens of times higher than their parent compounds in hepatocellular carcinoma [23,24].

Thiazolidinedione (TZD) is a five-membered thiazolidine ring with carbonyl groups at the two and four positions. It has a high dipole moment and can form hydrogen bonds with target proteins, which could be favorable in the binding of biomolecules. TZD was introduced in the late 1990s as a potential antidiabetic agent for treating type II diabetes [25]. In addition, TZD has many biological activities, including antibacterial, anticancer, anti-arthritic, anti-inflammatory, antioxidant, and so on [26]. TZD regulates apoptosis and proliferation in various cancer cell lines [27,28]. Steven D. Knight et al. have reported that the TZD derivative GSK1059615 can exert anticancer effects by inhibiting PI3K-α [29]. Liu et al. reported a TZD with potential anticancer activity through the Raf/MEK/ERK and PI3K/Akt signaling pathways [30]. Several anticancer drugs containing TZD moieties are currently undergoing clinical trials, including chemical I (S-49076 hydrochloride) for the treatment of solid tumors and chemical II (inolitazone hydrochloride) for the treatment of colorectal cancer (Figure 1). Therefore, TZD moieties can be considered privileged structures in the design of antitumor agents [31,32].

We decided to incorporate the TZD moiety into the C-3 position of lupeol with ester, piperazine–carbamate, or ethylenediamine as a linker. In this way, we designed the TZD-conjugated compounds **6a**–**i**, **9a**–**i**, and **12a**–**i** to improve the potential of lupeol against different cancer cells (Figure 1). The target compounds were synthesized, and their antitumor activities against the human non-small cell lung cancer cells (A549), human breast cancer cells (MCF-7), human hepatocellular carcinomas cells (HepG2), and human hepatic cells (LO2) were evaluated. In addition, the mechanism of action of compound **12i** was further investigated.

## 2. Results and Discussion

### 2.1. Synthesis of Lupeol Derivatives

#### 2.1.1. Synthesis of Intermediates **5a**–**i**

**Scheme 1:**
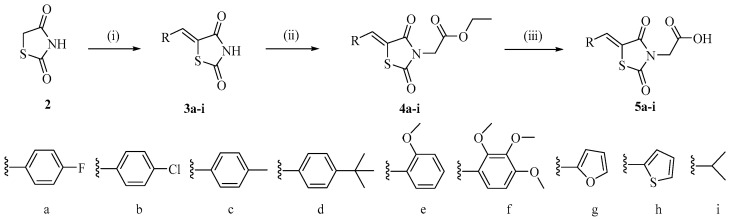
Synthesis of intermediates **5a**–**i**. (i) Different aldehydes, piperidine, EtOH, reflux, 8–10 h, 76–88%; (ii) ethyl bromoacetate, K_2_CO_3_, KI, DMF, 50 °C, 5–7 h, 67–82%; (iii) AcOH, HCl, reflux, 5–8 h, 58–72%.

Intermediates **3a**–**i** were obtained via the Knoevenagel condensation reaction of 2,4-thiazolidinedione (**2**) with different aldehydes in the presence of piperidine as the catalyst [33]. Intermediates **3a**–**i** were alkylated with ethyl bromoacetate, yielding intermediates **4a**–**i**. Subsequent hydrolysis under acidic conditions yielded **5a**–**i**. This synthetic route is detailed in Scheme 1.

#### 2.1.2. Synthesis of Target Compounds **6a**–**i**

**Scheme 2:**
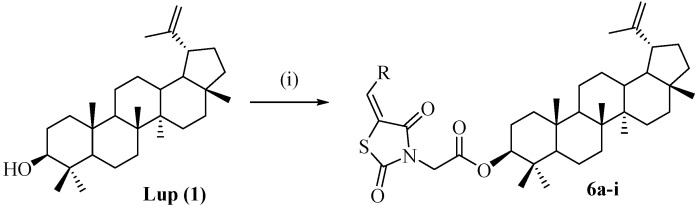
Synthesis of target compounds **6a**–**i**. (i) **5a**–**i**, EDCI, DMAP, CH_2_Cl_2_, rt, 8–10 h, 35–70%.

The esterification of lupeol (**1**) with intermediates **5a**–**i** in the presence of 1-ethyl-3-(3-dimethylaminopropyl)carbodiimide (EDCI) and 4-dimethylaminopyridine (DMAP) led to target compounds **6a**–**i**. The synthetic route is detailed in Scheme 2.

#### 2.1.3. Synthesis of Target Compounds **9a**–**i**

**Scheme 3:**
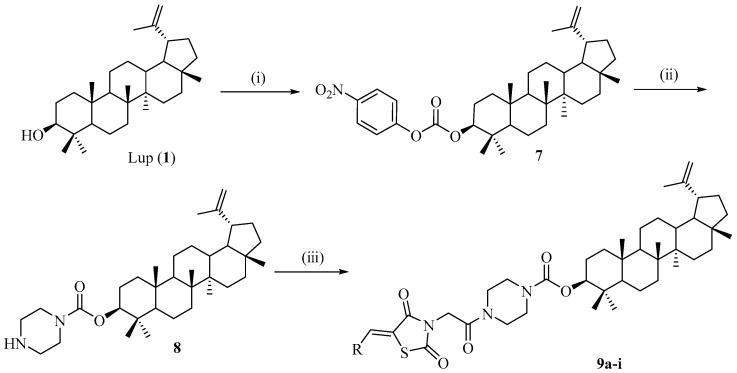
Synthesis of target products **9a**–**i**. (i) 4-nitrophenyl chloroformate, pyridine, CH_2_Cl_2_, 0-rt, 1–2 h, 80%; (ii) piperazine, Et_3_N, CH_2_Cl_2_, rt, 4–5 h, 75%; (iii) **5a**–**i**, EDCI, DMAP, CH_2_Cl_2_, rt, 6–8 h, 38–62%.

A substitution reaction of the C-3 hydroxyl lupeol group and 4-nitrophenyl chloroformate in the presence of pyridine produced compound **7**; compound **7** was then reacted with piperazine to introduce a carbamate at the C-3 position to produce compound **8**. Compound **8** reacted with compounds **5a**–**i** via amidation to obtain compounds **9a**–**i**. The synthetic route is detailed in Scheme 3.

#### 2.1.4. Synthesis of Target Compounds **12a**–**i**

**Scheme 4:**
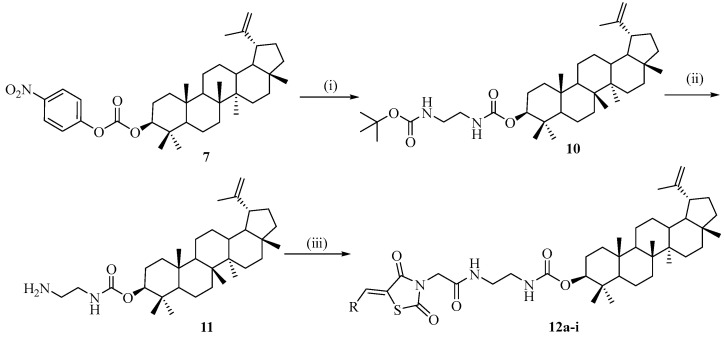
Synthesis of target products **12a**–**i**. (i) N-Boc-ethylenediamine, Et_3_N, CH_2_Cl_2_, rt, 10–12 h, 79%; (ii) CF_3_COOH, CH_2_Cl_2_, 0 °C-rt, overnight, 70%; (iii) **5a**–**i**, EDCI, HOBT, DIPEA, CH_2_Cl_2_, 0-rt, 8–10 h, 32–58%.

A substitution reaction occurred between compound **7** and N-Boc-ethylenediamine in the presence of Et_3_N, producing compound **10**. Then, the N-Boc protection was removed using a trifluoroacetic acid (TFA) treatment to obtain compound **11**, which then reacted with compounds **5a**–**i** in the presence of EDCI, N-hydroxybenzotrizole (HOBT), and N, N-diisopropylethylamine (DIPEA) to obtain compounds **12a**–**i**. The synthetic route is detailed in Scheme 4.

### 2.2. Biological Evaluation

#### 2.2.1. In Vitro Antiproliferative Activity and Structure–Activity Relationship Studies

The antitumor activities of compounds **6a**–**i**, **9a**–**i**, and **12a**–**i** toward human lung cancer A549, human breast cancer MCF-7, human hepatocarcinoma HepG2, and human hepatic LO2 cells were determined using the (3-(4,5-dimethylthiazol-2-yl)-2,5-diphenyltetrazolium bromide (MTT) assay. Lupeol and cisplatin were used as the positive controls. The IC_50_ results are shown in Table 1.

Table 1 shows that all of the synthesized lupeol-3-thiazolidinedione derivatives (**6a**–**i**, **9a**–**i**, and **12a**–**i**) showed significantly higher antitumor activities than the parent lupeol against all three cancer cell lines. Compounds **9a**–**i** contained a piperazine–carbamate linker, and compounds **12a**–**i** contained an ethylenediamine linker, which showed good antitumor activity. Compound **12i** showed the most significant antitumor activity against the three cancer cell lines, with the IC_50_ values ranging from 4.40 to 10.06 μM. The antitumor activity against HepG2 cells was 9.91-fold higher than that of lupeol. In the A549 cell line, compounds **6c**, **6e**, **6f**, **6i**, **9d**, **9e**, **9f**, **9g**, **12a**, **12f**, and **12i** showed the most significant antitumor activities, with IC_50_ values ranging from 9.24 to 11.71 μM. In the MCF-7 cell lines, compounds **6h**, **6i**, **9f**, **12d**, **12f**, and **12i** showed significant antitumor activity, with the IC_50_ values ranging from 8.70 to 11.34 μM. In the HepG2 cell line, compounds **6d**, **6i**, **9d**, **9f**, **9i**, **12c**, **12f**, and **12i** showed the most significant antitumor activity, with IC_50_ values ranging from 4.40 to 10.86 μM. In addition, the IC_50_ values of all compounds tested were greater than 28 μM, thus demonstrating that these compounds exhibited relatively weak cytotoxicity against the human hepatic LO2 cells and were selective to a certain extent for cancer cells.

Based on the preliminary conformational relationships, we found that the introduction of thiazolidinedione, as well as these three types of linkers (ester, piperazine-carbamate, and ethylenediamine), significantly enhances the antitumor activity of the compounds. This discovery underscores the potential of these specific molecular modifications in the development of more effective cancer therapies. We also found that the effect of the substituents on the antitumor activity of compounds **6i**, **9i**, and **12i** was more significant than that of the compounds **6a**–**h**, **9a**–**h**, and **12a**–**h**. This indicates that the antitumor activity of the aliphatic substituent is superior to that of the aromatic substituents. The antitumor activities of compounds **6c**–**f**, **9c**–**f**, and **12c**–**f**, containing electron donor groups such as -CH_3_, -C(CH_3_)_3_, and -OCH_3_, among others, are generally superior to those of compounds **6a**–**b**, **9a**–**b**, and **12a**–**b**, containing electron-withdrawing groups such as -F and -Cl.

Compound **12i** showed the strongest antitumor activity. Thus, we further investigated its effects and mechanism regarding in vitro anti-hepatocellular carcinoma activity.

#### 2.2.2. Compound **12i** Induced Apoptosis in HepG2 Cells

**Figure 2:**
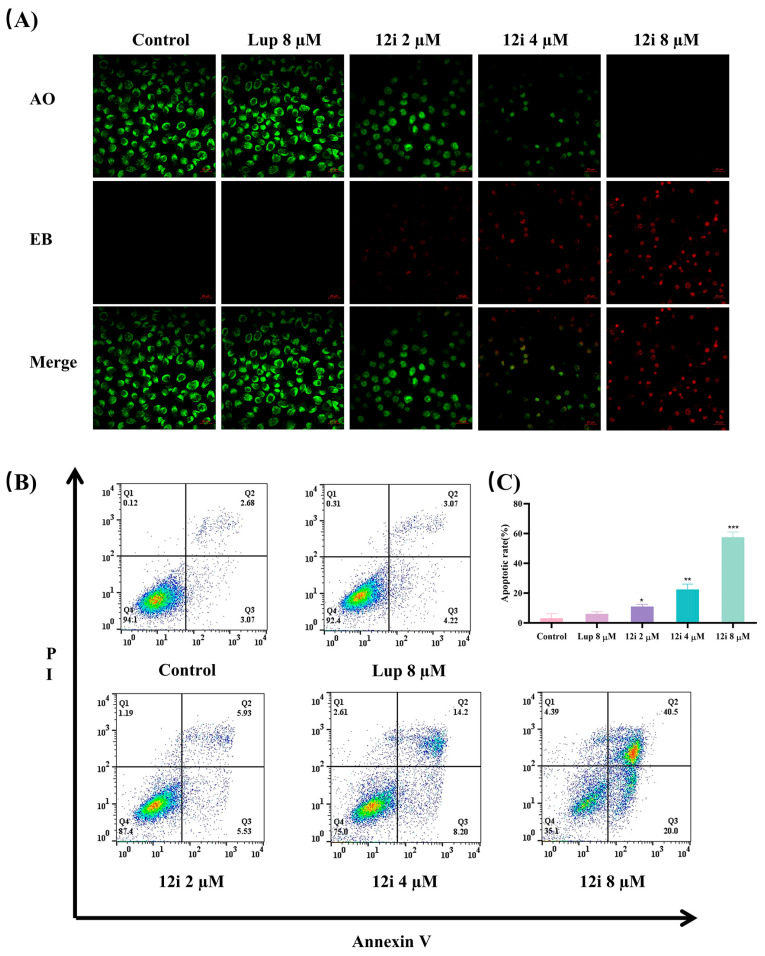
Compound **12i** induces HepG2 cell apoptosis. (**A**) HepG2 cells were treated with **12i** (2, 4, and 8 µM) or 0.1% DMSO (blank control) and then stained with AO/EB. Normal cells were stained green with AO staining solution with intact nuclei, apoptotic cells were stained red with EB staining solution. (**B**) Detection of apoptosis in HepG2 cells treated with various concentrations (0, 2, 4, and 8 µM) of **12i** for 48 h using an Annexin V-FITC/PI double-staining assay. (**C**) Percentage of cell apoptosis for different concentrations of **12i** (* *p* < 0.05, ** *p* < 0.01, and *** *p* < 0.001 compared with the blank control group). Error bars are representative of three independent experiments.

To further evaluate compound **12i**-induced HepG2 cell apoptosis, HepG2 cells were treated with this compound (at 0, 2, 4, and 8 μM), followed by staining with Acridine Orange/Ethidium bromide (AO/EB). The morphological variations in the cells were observed using fluorescence microscopy, as shown in Figure 2A, and the nuclei of the cells in the control group were intact and stained green by AO. With the increasing concentration of compound **12i**, the cell membrane ruptured; the nuclei crumpled; and the green fluorescence gradually weakened and turned orange–red. Subsequently, we used Annexin V-FITC/PI double staining to characterize the effect of compound **12**i based on the total apoptosis rate of HepG2 cells. The apoptosis rate was detected via flow cytometry, as shown in Figure 2B,C. The total apoptosis rate of the HepG2 cells increased with the increasing concentrations of compound **12i** compared with the blank control. It significantly increased from 5.75% in the blank control to 11.46%, 22.40%, and 60.50%. Thus, compound **12i** induces HepG2 apoptosis.

#### 2.2.3. Effects of **12i** on Reactive Oxygen Species (ROS) Generation

**Figure 3:**
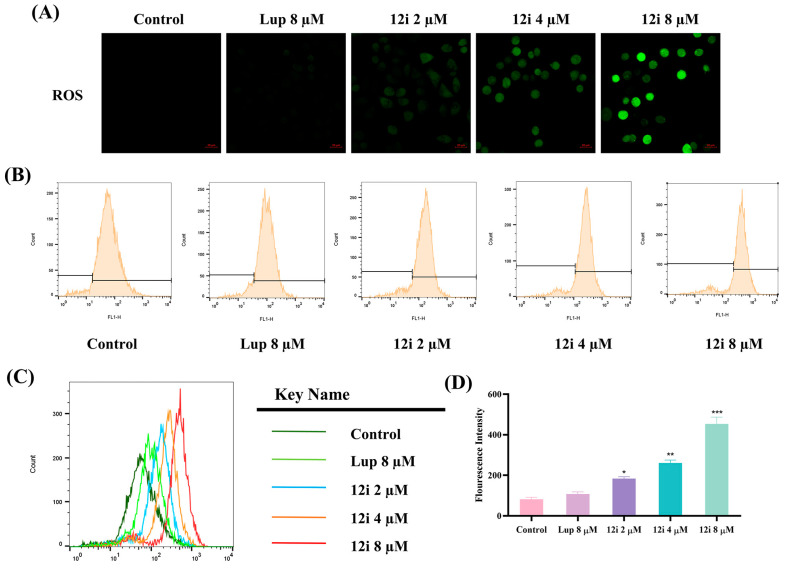
Compound **12i** induces ROS generation in HepG2 cells. (**A**) HepG2 cells were treated with **12i** (2, 4, and 8 µM) and 0.1% DMSO (blank control) and then stained with DCFH-DA. (**B**,**C**) Determination of ROS content in HepG2 cells. (**D**) ROS content for different **12i** concentrations (* *p* < 0.05, ** *p* < 0.01, and *** *p* < 0.001 compared with the blank control group). Error bars represent three independent experiments.

ROS are a key element in cancer therapy [34]. To evaluate whether compound **12i** can affect ROS production in HepG2 cells, we used 2′,7′-dichlorofluorescein diacetate (DCFH-DA) staining to determine the ROS levels. After 48 h, morphological variations in the cells were observed using fluorescence microscopy, as shown in Figure 3A. The green fluorescence of the **12i**-treated experiments was more obvious than that of the blank control, and the green fluorescence of **12i** (8 μM) was the most obvious. According to the above data, compound **12i** induces ROS production dose-dependently in HepG2 cells. Flow cytometry was used to detect changes in HepG2 cell ROS content, and the results are shown in Figure 3B–D. The above data indicates that compound **12i** significantly increased the production of ROS, which may also be responsible for its induction of apoptosis.

#### 2.2.4. Effects of **12i** on Mitochondria Membrane Potential (MMP)

**Figure 4:**
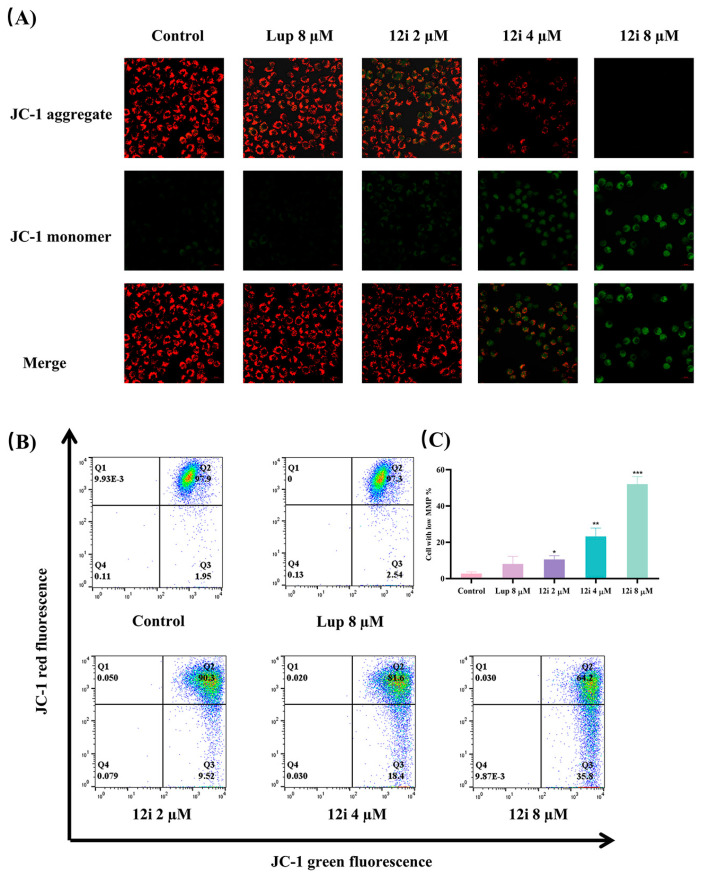
Compound **12i** induces MMP in HepG2 cells. (**A**) HepG2 cells were treated with **12i** (2, 4, and 8 µM) or 0.1% DMSO (blank control) and then stained with JC-1. (**B**) Determination of MMP depolarization rate in HepG2 cells. (**C**) Percentage of decreasing MMP for different **12i** concentrations (* *p* < 0.05, ** *p* < 0.01, and *** *p* < 0.001 compared with the blank control group). Error bars represent three independent experiments.

MMP depolarization is one of the landmark events of apoptosis [35]. To investigate the impact of **12i** on MMP depolarization in HepG2 cells, we used 5,5′,6,6,6,6′-tetrachloro-1,1′,3,3′-tetraethylioiodine carbocyan (JC-1) staining. When the mitochondrial membrane potential is high, JC-1 aggregates in the substrate to form a polymer (J-aggregates), which produces red fluorescence. When the mitochondrial membrane potential is low, JC-1 cannot accumulate in the mitochondrial matrix and exists as a monomer to produce green fluorescence. As shown in Figure 4A, the compound **12i**-treated experimental groups showed increased green fluorescence and decreased red fluorescence, with increasing concentrations compared with the blank control. Thus, compound **12i** could significantly reduce Δψm. The flow cytometry results are shown in Figure 4B,C. The compound **12i** concentration was positively correlated with the monomer rate of JC-1; the monomer rate of JC-1 increased from 1.95% in the blank control group to 35.8% in the highest concentration group (8 μM). Altogether, these data suggest that compound **12i** decreased the membrane potential of HepG2 cells.

#### 2.2.5. Effects of **12i** on the Mitochondrial Apoptosis Pathway

**Figure 5:**
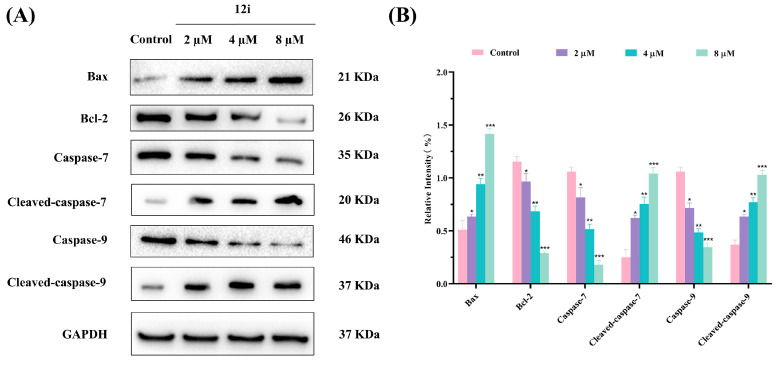
Compound **12i** inhibited the mitochondrial apoptosis pathway. (**A**) Western blot analysis of the expression levels of Bax, Bcl-2, caspase-7, cleaved-caspase-7, caspase-9, and cleaved-caspase-9 proteins in HepG2 cells treated with **12i** (2, 4, and 8 µM) and 0.1% DMSO (the blank control group). (**B**) Statistical analysis (* *p* < 0.05, ** *p* < 0.01, and *** *p* < 0.001 compared with the blank control group). Error bars represent three independent experiments.

Compound **12i** induces HepG2 cell apoptosis. To further demonstrate this mechanism, we used a Western blot to detect the expression levels of the relevant apoptotic proteins. The GAPDH expression level was used as an internal control group. Figure 5A,B shows that compound **12i** can downregulate the anti-apoptotic protein Bcl-2 and upregulate the pro-apoptotic protein Bax in HepG2 cells. Compound **12i** also activated the expression levels of both cleaved caspase-7 and cleaved caspase-9 dose dependently. These data suggest that compound **12i** induces HepG2 cell apoptosis, possibly via the mitochondrial apoptotic pathway.

#### 2.2.6. Effects of **12i** on the PI3K/AKT/mTOR Pathway

**Figure 6:**
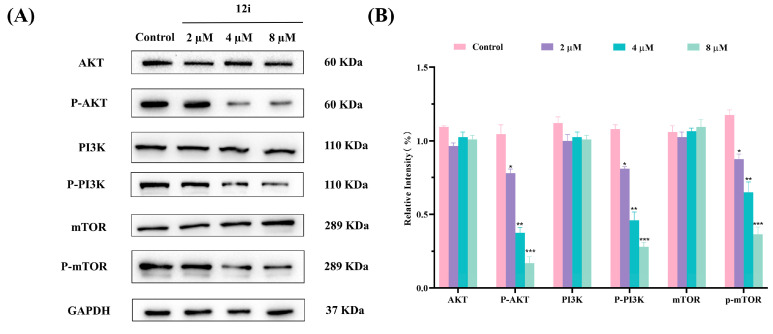
Compound **12i** inhibits the PI3K/Akt/mTOR pathway. (**A**) Western blot analysis of the expression levels of Akt, P-Akt, PI3K, P-PI3K, mTOR, and P-mTOR in HepG2 cells after exposure to the blank control and different concentrations (2, 4, and 8 µM) of **12i** for 48 h. (**B**) The percentage of cell apoptosis for different concentrations of **12i** (*: *p* < 0.05, **: *p* < 0.01, ***: *p* < 0.001, compared to the blank control group) Error bars are representatives of three independent experiments.

The literature reports suggest that lupeol can exert antitumor effects through the phosphoinositide 3-kinase (PI3K)/protein kinase B (AKT)/mammalian target of the rapamycin (mTOR) pathway, and thiazolidinedione has also been shown to induce cancer cell apoptosis via the PI3K/AKT/mTOR pathway. Therefore, we predict that compound **12i** may also exhibit antitumor activity through the PI3K/AKT/mTOR pathway. Therefore, Western blotting was conducted to determine the expression of the key proteins Akt, P-Akt, PI3K, P-PI3K, mTOR, and P-mTOR. As shown in Figure 6A,B, compound **12i** was able to reduce the phosphorylation levels of P-Akt, P-PI3K, and P-mTOR in a dose-dependent manner. The results indicate that compound **12i** can inhibit the PI3K/AKT/mTOR pathway.

#### 2.2.7. Compound **12i** Is Predicted to Bind to PI3K

**Figure 7:**
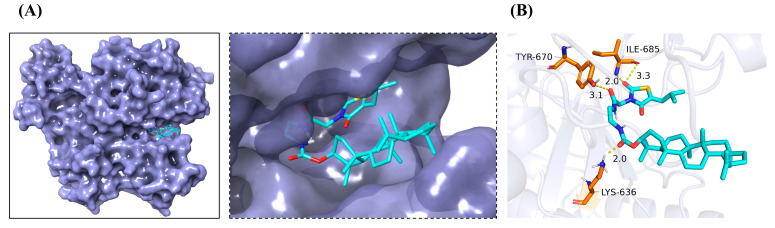
There was an eutectic structure of compound **12i** with PI3K (PDB: 4OYS). (**A**) Modeled and enlarged close-up of the surface mosaic of PI3K tetramer and **12i** binding. (**B**) Close-up of **12i** interactions in the PI3K allosteric binding pocket. Here, **12i** was rendered as a rod and colored according to atom type. Cyan denoted carbon, blue denoted nitrogen, and red denoted oxygen. Key residual atoms in PI3K that interacted with the compound were denoted in cyan.

In order to investigate the binding mode of compound **12i** to PI3K, molecular docking studies based on the crystal structure of PI3K (PDB: 4OYS) were performed, which revealed that **12i** interacts with different reactive sites in the 4OYS variable pocket, as shown in Figure 7. Amino acid residues TRY-670 and ILE-685 form hydrogen bonds with the carbonyl groups at positions C34 and C36 in compound **12i**. Perhaps this hydrogen bonding interaction pushes the bonds and substituents deep into the narrow lumen of 4OYS. Perhaps it can more efficiently promote the binding of the carbonyl group at position C31 to the active center lysine LYS-636 in the narrow grooves of 4OYS, which may affect cell growth and reproduction or the regulation of mitochondrial function. In particular, the binding of the protein–ligand complex could reach −9.07 kcal/mol, which is a good indication that compound **12i** has good inhibitory activity against PI3K.

## 3. Materials and Methods

### 3.1. Chemistry

#### 3.1.1. Reagents and Instruments

Thiazolidinedione was purchased from Anhui Zesheng Technology Co., Ltd. (Shanghai, China), and the other reagents (analytical grade) were purchased from Tianjin Fuyu Fine Chemical Co., Ltd. (Tianjin, China). The progress of all chemical reactions was carefully monitored via thin-layer chromatography (TLC) using silica gel GF254 (Qingdao Ocean Chemical Co., Ltd., Qingdao, China) as the stationary phase. Column chromatography purified the intermediates and target derivatives (300–400-mesh silica gel, Yantai Yinlong Silica Gel Co., Ltd., Yantai, China). ^1^H NMR and ^13^C NMR analyses of the target compounds were conducted using a Bruker NMR instrument (Bruker Avance DRX400). The target compounds were also characterized using a Bruker high-resolution mass spectrometer (Bruker Esquire 6000). The Supplementary Material includes HRMS, 1H NMR, and 13C NMR spectral data of all derivatives.

#### 3.1.2. Synthesis of Intermediates **3a**–**i**

Compound **2** (TZD, 500 mg, 4.26 mmol) was refluxed with different aldehydes (1.00 equiv) and piperidines (1.00 equiv) in EtOH (10 mL), stirring for 8–10 h, and monitored via TLC until the reaction was complete. The solvent was poured into water, and the pH of the system was adjusted to 7 with HCl (37%). The organic phase was separated, and the aqueous phase was extracted with ethyl acetate, washed with saturated salted water, dried with anhydrous Na_2_SO_4_, and concentrated under reduced pressure. The crude products (**3a**–**i**) were directly carried out in the next step of the reaction.

#### 3.1.3. Synthesis of Intermediates **4a**–**i**

Compounds **3a**–**i** (500 mg, 1.69–2.93 mmol), K_2_CO_3_ (1.20 equiv), KI (1.00 equiv), and ethyl bromoacetate (1.00 equiv) were added to a round bottom flask, containing 8 mL of dimethylformamide (DMF). TLC monitored the reaction until there were no ingredients left. The sample was extracted with dichloromethane (CH_2_Cl_2_) and washed with saturated salted water; the organic phases were then separated and combined. The organic phase was dried with anhydrous Na_2_SO_4_ and filtered. The solvent was removed under reduced pressure, and the residue was purified via silica gel column chromatography (PE/EA = 20:1, *v*/*v*) to obtain **4a**–**i**.

#### 3.1.4. Synthesis of Intermediates **5a**–**i**

A mixture of compounds **4a**–**i** (400 mg, 1.04–1.56 mmol), HCl (37%, 2 mL), and AcOH (3 mL) was refluxed for 5–8 h, using TLC to monitor the reaction. After the reaction, the solution was extracted with ethyl acetate, washed with saturated salted water, dried over Na_2_SO_4_, and concentrated under reduced pressure to yield the corresponding intermediates **5a**–**i**.

#### 3.1.5. Synthesis of Compounds **6a**–**i**

A mixture of lupeol (**1**, 50 mg, 0.12 mmol), intermediates **5a**–**i** (0.18mmol), EDCI (34.42 mg, 0.18 mmol), and DMAP (14.66 mg, 0.12 mmol) in CH_2_Cl_2_ (6 mL) were stirred for 8–12 h. Throughout this period, TLC analysis monitored the process until the starting material completely disappeared. The solvent was then removed under reduced pressure, resulting in crude product formation. Further purification involved separation via silica gel column chromatography using CH_2_Cl_2_ as a developing agent. This process produced target products **6a**–**i**.

##### Lupeol-3-[(1-Ethyl)-2]-5-(4-Fluorobenzylidene)-2,4-Thiazolidinedione (**6a**)

White solid, yield: 65%; mp: 176.8–178.5 °C.^1^H NMR (600 MHz, chloroform-*d*) *δ* 7.90 (s, 1H, CH_2_=CH_2_), 7.52 (dd, *J* = 8.5, 5.2 Hz, 2H, Ar-H), 7.19 (t, *J* = 8.4 Hz, 2H, Ar-H), 4.68 (d, *J* = 2.4 Hz, 1H, CH_2_=CH_2_), 4.56 (s, 1H, CH_2_=CH_2_), 4.54 (d, *J* = 4.7 Hz, 1H, CH), 4.47 (s, 2H, CH_2_), 2.37 (td, *J* = 11.0, 5.8 Hz, 1H), 1.94–1.88 (m, 1H), 1.68 (s, 3H, CH_3_), 1.67–1.61 (m, 6H), 1.48 (ddd, *J* = 13.0, 8.2, 3.1 Hz, 2H), 1.38 (q, *J* = 8.3, 7.9 Hz, 6H), 1.34 (d, *J* = 2.7 Hz, 1H), 1.29 (d, *J* = 2.5 Hz, 1H), 1.26 (d, *J* = 7.9 Hz, 2H), 1.23–1.09 (m, 3H), 1.06 (dd, *J* = 12.9, 4.7 Hz, 1H), 1.02 (s, 3H, CH_3_), 1.00–0.95 (m, 2H), 0.93 (s, 3H, CH_3_), 0.86 (s, 3H, CH_3_), 0.83 (s, 3H, CH_3_), 0.78 (d, *J* = 4.5 Hz, 6H, CH_3_×2). ^13^C NMR (150 MHz, chloroform-*d*) *δ* 167.29 (C33), 165.99 (C35), 165.68 (C31), 164.77 (C20), 163.08 (C40), 151.09 (C38), 133.39 (C37), 132.56 (C42), 132.50 (C36), 120.85 (C39), 116.85 (C34), 116.70 (C41), 109.52 (C29), 55.43 (C3), 50.41 (C5), 48.39 (C9), 48.12 (C18), 43.12 (C19), 42.96 (C17), 42.56 (C14), 40.95 (C32), 40.12 (C8), 38.38 (C22), 38.14 (C13), 37.97 (C1), 37.16 (C4), 35.68 (C10), 34.27 (C16), 29.94 (C7), 29.84 (C21), 28.04 (C15), 27.54 (C12), 25.17 (C2), 23.69 (C23), 21.07 (C24), 19.40 (C11), 18.26 (C30), 18.13 (C28), 16.52 (C6), 16.24 (C25), 16.09 (C26), 14.65 (C27). HRMS (ESI) *m*/*z*: calcd for C_42_H_56_NO_4_FNaS [M+Na]^+^ 712.3812, found 712.3804.

##### Lupeol-3-[(1-Ethyl)-2]-5-(4-Chlorobenzylidene)-2,4-Thiazolidinedione (**6b**)

Yellow solid, yield: 70%; mp: 168.6–170.4 °C. ^1^H NMR (600 MHz, chloroform-*d*) *δ* 7.88 (s, 1H, CH_2_=CH_2_), 7.46 (s, 4H, Ar-H), 4.68 (d, *J* = 2.4 Hz, 1H, CH_2_=CH_2_), 4.56 (d, *J* = 3.1 Hz, 1H, CH_2_=CH_2_), 4.54 (d, *J* = 4.8 Hz, 1H, CH), 4.47 (d, *J* = 1.7 Hz, 2H, CH_2_), 2.37 (dt, *J* = 11.1, 5.5 Hz, 1H), 1.91 (ddd, *J* = 13.0, 6.9, 2.7 Hz, 1H), 1.68 (s, 3H, CH_3_), 1.67–1.61 (m, 6H), 1.48 (td, *J* = 8.2, 3.9 Hz, 2H), 1.40–1.36 (m, 6H), 1.34–1.32 (m, 1H), 1.31–1.29 (m, 1H), 1.26 (d, *J* = 7.6 Hz, 2H), 1.24–1.14 (m, 3H), 1.06 (dd, *J* = 12.9, 4.7 Hz, 1H), 1.02 (s, 3H, CH_3_), 1.00–0.95 (m, 2H), 0.93 (s, 3H, CH_3_), 0.86 (s, 3H, CH_3_), 0.83 (s, 3H, CH_3_), 0.78 (d, *J* = 3.8 Hz, 6H, CH_3_×2). ^13^C NMR (150 MHz, chloroform-*d*) *δ* 167.13 (C33), 165.94 (C35), 165.59 (C31), 151.09 (C20), 137.02 (C40), 133.18 (C38, C42), 131.68 (C37), 131.52 (C36), 129.76 (C39, C41), 121.80 (C34), 109.52 (C29), 83.57 (C3), 55.43 (C5), 50.41 (C9), 48.39 (C18), 48.12 (C19), 43.12 (C17), 42.96 (C14), 42.58 (C32), 40.95 (C8), 40.12 (C22), 38.38 (C13), 38.14 (C1), 37.97 (C4), 37.16 (C10), 35.68 (C16), 34.27 (C7), 29.94 (C21), 28.05 (C15), 27.54 (C12), 25.17 (C2), 23.70 (C23), 21.07 (C24), 19.40 (C11), 18.26 (C30), 18.13 (C28), 16.52 (C6), 16.24 (C25), 16.09 (C26), 15.06 (C27). HRMS (ESI) *m*/*z*: calcd for C_42_H_56_NO_4_NaSCl [M+Na]^+^ 728.3516, found 728.3510.

##### Lupeol-3-[(1-Ethyl)-2]-5-(4-Methylbenzylidene)-2,4-Thiazolidinedione (**6c**)

Light-yellow solid, yield: 62%; mp: 182.7–184.5 °C. ^1^H NMR (600 MHz, chloroform-*d*) *δ* 7.91 (s, 1H, CH_2_=CH_2_), 7.42 (d, *J* = 7.7 Hz, 2H, Ar-H), 7.29 (d, *J* = 7.8 Hz, 2H, Ar-H), 4.68 (s, 1H, CH_2_=CH_2_), 4.56 (s, 1H, CH_2_=CH_2_), 4.54 (d, *J* = 4.5 Hz, 1H, CH), 4.47 (s, 2H, CH_2_), 2.41 (s, 3H, CH_3_), 2.36 (dd, *J* = 11.0, 5.8 Hz, 1H), 1.94–1.87 (m, 1H), 1.68 (s, 3H, CH_3_), 1.67–1.59 (m, 6H), 1.47 (dd, *J* = 15.3, 6.0 Hz, 2H), 1.45–1.36 (m, 6H), 1.36 (d, *J* = 4.5 Hz, 1H), 1.34 (s, 1H), 1.32–1.24 (m, 2H), 1.24–1.08 (m, 3H), 1.06 (dd, *J* = 12.9, 4.4 Hz, 1H), 1.01 (s, 3H, CH_3_), 0.97 (d, *J* = 12.0 Hz, 2H), 0.93 (s, 3H, CH_3_), 0.84 (d, *J* = 15.6 Hz, 6H, CH_3_), 0.78 (s, 6H, CH_3_×2). ^13^C NMR (150 MHz, chloroform-*d*) *δ* 167.70 (C33), 166.07 (C35), 165.87 (C31), 151.08 (C20), 141.69 (C40), 134.82 (C37), 130.54 (C38, C42), 130.48 (C36), 130.17 (C39, C41), 119.89 (C34), 109.95 (C29), 83.46 (C3), 55.43 (C5), 50.40 (C9), 48.38 (C18), 48.12 (C19), 43.11 (C17), 42.95 (C14), 42.49 (C32), 40.94 (C8), 40.11 (C22), 38.81 (C13), 38.13 (C1), 37.95 (C4), 37.15 (C10), 36.04 (C16), 34.27 (C7), 29.93 (C21), 28.03 (C15), 27.54 (C12), 25.17 (C2), 23.68 (C23), 21.75 (C43), 21.06 (C24), 19.40 (C11), 18.25 (C30), 18.12 (C28), 16.50 (C6), 16.22 (C25), 16.08 (C26), 14.64 (C27). HRMS (ESI) *m/z*: calcd for C_43_H_59_NO_4_NaS [M+Na]^+^ 708.4062, found 708.4055.

##### Lupeol-3-[(1-Ethyl)-2]-5-(4-Tert-Butylphenyl)-2,4-Thiazolidinedione (**6d**)

White solid, yield: 45%; mp: 188.6–190.5 °C. ^1^H NMR (600 MHz, chloroform-*d*) *δ* 7.92 (s, 1H, CH_2_=CH_2_), 7.51 (d, *J* = 8.3 Hz, 2H, Ar-H), 7.47 (d, *J* = 8.3 Hz, 2H, Ar-H), 4.68 (d, *J* = 2.5 Hz, 1H, CH_2_=CH_2_), 4.56 (d, *J* = 2.7 Hz, 1H, CH_2_=CH_2_), 4.55–4.51 (m, 1H, CH), 4.47 (d, *J* = 2.9 Hz, 2H, CH_2_), 2.37 (td, *J* = 11.0, 5.8 Hz, 1H), 1.94–1.88 (m, 1H), 1.68 (s, 3H, CH_3_), 1.67–1.60 (m, 6H), 1.49–1.45 (m, 2H), 1.38 (d, *J* = 4.2 Hz, 6H), 1.35 (s, 9H, CH_3_×3), 1.32–1.29 (m, 1H), 1.29–1.22 (m, 2H), 1.22–1.09 (m, 3H), 1.09–1.04 (m, 1H), 1.01 (s, 3H, CH_3_), 0.97 (d, *J* = 11.1 Hz, 2H), 0.93 (s, 3H, CH_3_), 0.85 (s, 3H, CH_3_), 0.83 (s, 3H, CH_3_), 0.78 (s, 6H, CH_3_×2). ^13^C NMR (150 MHz, chloroform-*d*) *δ* 167.74 (C33), 166.08 (C35), 165.89 (C31), 154.72 (C20), 151.08 (C40), 134.69 (C37), 130.46 (C38, C42), 130.05 (C36), 126.46 (C39, C41), 120.00 (C34), 109.92 (C29), 83.47 (C3), 55.43 (C5), 50.40 (C9), 48.39 (C18), 48.13 (C19), 43.11 (C17), 42.96 (C14), 42.51 (C32), 40.95 (C8), 40.12 (C22), 38.38 (C13), 38.14 (C1), 37.95 (C4), 37.15 (C10), 35.68 (C16), 35.23 (C43), 34.27 (C7), 31.20 (C44, C45, C46), 29.94 (C21), 28.04 (C15), 27.54 (C12), 25.17 (C2), 23.68 (C23), 21.07 (C24), 19.40 (C11), 18.26 (C30), 18.13 (C28), 16.50 (C6), 16.23 (C25), 16.08 (C26), 14.65 (C27). HRMS (ESI) *m*/*z*: calcd for C_46_H_65_NO_4_NaS [M+Na]^+^ 750.4532, found 750.4532.

##### Lupeol-3-[(1-Ethyl)-2]-5-(2-Methoxybenzylidene)-2,4-Thiazolidinedione (**6e**)

White solid, yield: 43%; mp: 174.8–176.5 °C. ^1^H NMR (600 MHz, chloroform-*d*) *δ* 8.31 (s, 1H, CH_2_=CH_2_), 7.45 (dd, *J* = 7.8, 1.6 Hz, 1H, Ar-H), 7.43–7.39 (m, 1H, Ar-H), 7.05 (t, *J* = 7.6 Hz, 1H, Ar-H), 6.95 (d, *J* = 8.3 Hz, 1H, Ar-H), 4.68 (d, *J* = 2.4 Hz, 1H, CH_2_=CH_2_), 4.56 (d, *J* = 2.4 Hz, 1H, CH_2_=CH_2_), 4.54 (t, *J* = 5.7 Hz, 1H, CH), 4.47 (d, *J* = 3.1 Hz, 2H, CH_2_), 3.90 (s, 3H, OCH_3_), 2.37 (td, *J* = 11.1, 5.8 Hz, 1H), 1.91 (ddd, *J* = 13.1, 7.1, 2.9 Hz, 1H), 1.68 (s, 3H, CH_3_), 1.67–1.58 (m, 6H), 1.51–1.46 (m, 2H), 1.39 (q, *J* = 4.9, 3.9 Hz, 6H), 1.36 (d, *J* = 3.9 Hz, 1H), 1.34–1.32 (m, 1H), 1.32–1.25 (m, 2H), 1.25–1.08 (m, 3H), 1.06 (dd, *J* = 12.8, 4.7 Hz, 1H), 1.02 (s, 3H, CH_3_), 1.01–0.95 (m, 2H), 0.93 (s, 3H, CH_3_), 0.86 (s, 3H, CH_3_), 0.83 (s, 3H, CH_3_), 0.78 (d, *J* = 5.8 Hz, 6H, CH_3_×2). ^13^C NMR (150 MHz, chloroform-*d*) *δ* 168.13 (C33), 166.17 (C35), 165.88 (C31), 158.71 (C20), 151.09 (C40), 132.60 (C37), 130.50 (C36), 129.59 (C39), 122.41 (C41), 121.03 (C38, C42), 111.32 (C34), 109.92 (C29), 83.41 (C3), 55.66 (C43), 55.44 (C5), 50.40 (C9), 48.39 (C18), 48.13 (C19), 43.11 (C17), 42.96 (C14), 42.43 (C32), 40.95 (C8), 40.12 (C22), 38.39 (C13), 38.14 (C1), 37.96 (C4), 37.16 (C10), 35.68 (C16), 34.28 (C7), 29.94 (C21), 28.05 (C15), 27.54 (C12), 25.18 (C2), 23.69 (C23), 21.07 (C24), 19.40 (C11), 18.26 (C30), 18.13 (C28), 16.51 (C6), 16.24 (C25), 16.08 (C26), 14.65 (C27). HRMS (ESI) *m*/*z*: calcd for C_43_H_59_NO_5_NaS [M+Na]^+^ 724.4012, found 724.4009.

##### Lupeol-3-[(1-Ethyl)-2]-5-(2,3,4-Trimethoxybenzylidene)-2,4-Thiazolidinedione (**6f**)

Light-yellow solid, yield: 35%; mp: 183.6–185.5 °C. ^1^H NMR (600 MHz, chloroform-*d*) *δ* 8.19 (s, 1H, CH_2_=CH_2_), 7.21 (d, *J* = 8.7 Hz, 1H, Ar-H), 6.78 (d, *J* = 8.8 Hz, 1H, Ar-H), 4.68 (d, *J* = 2.5 Hz, 1H, CH_2_=CH_2_), 4.57–4.56 (m, 1H, CH_2_=CH_2_), 4.54 (d, *J* = 4.5 Hz, 1H, CH), 4.47 (d, *J* = 3.9 Hz, 2H, CH_2_), 3.94 (d, *J* = 9.8 Hz, 6H, OCH_3_×2), 3.89 (s, 3H, OCH_3_), 2.37 (td, *J* = 11.0, 5.8 Hz, 1H), 1.94–1.87 (m, 1H), 1.68 (s, 3H, CH_3_), 1.67–1.61 (m, 6H), 1.50–1.46 (m, 2H), 1.45–1.37 (m, 6H), 1.36 (d, *J* = 4.0 Hz, 1H), 1.34 (d, *J* = 2.3 Hz, 1H), 1.25 (s, 2H), 1.23–1.11 (m, 3H), 1.06 (dd, *J* = 12.9, 4.5 Hz, 1H), 1.02 (s, 3H, CH_3_), 1.00–0.95 (m, 2H), 0.93 (s, 3H, CH_3_), 0.86 (s, 3H, CH_3_), 0.83 (s, 3H, CH_3_), 0.78 (d, *J* = 4.4 Hz, 6H, CH_3_×2). ^13^C NMR (150 MHz, chloroform-*d*) *δ* 168.16 (C33), 166.22 (C35), 165.92 (C31), 156.51 (C20), 154.10 (C40), 151.11 (C37), 142.52 (C38), 130.08 (C36), 124.83 (C42), 120.44 (C39), 119.69 (C41), 109.52 (C34), 107.73 (C29), 83.42 (C3), 62.04 (C43), 61.12 (C44), 56.31 (C45), 55.45 (C5), 50.41 (C9), 48.82 (C18), 48.14 (C19), 43.12 (C17), 42.96 (C14), 42.43 (C32), 40.96 (C8), 40.13 (C22), 38.40 (C13), 38.15 (C1), 37.97 (C4), 37.16 (C10), 35.69 (C16), 34.28 (C7), 29.95 (C21), 29.84 (C11), 28.05 (C15), 27.55 (C12), 25.18 (C2), 23.69 (C23), 21.07 (C24), 18.27 (C30), 18.13 (C28), 16.51 (C6), 16.24 (C25), 16.09 (C26), 14.66 (C27). HRMS (ESI) *m*/*z*: calcd for C_45_H_63_NO_7_NaS [M+Na]^+^ 784.4223, found 784.4225.

##### Lupeol-3-[(1-Ethyl)-2]-5-(2-Furanylmethylene)-2,4-Thiazolidinedione (**6g**)

Light-brown solid, yield: 48%; mp: 185.4–187.2 °C. ^1^H NMR (600 MHz, chloroform-*d*) *δ* 7.68 (d, *J* = 1.7 Hz, 1H, CH_2_=CH_2_), 7.67 (s, 1H, CH_2_=CH_2_), 6.81 (d, *J* = 3.5 Hz, 1H, CH_2_=CH_2_), 6.59 (dd, *J* = 3.6, 1.8 Hz, 1H, CH_2_=CH_2_), 4.68 (d, *J* = 2.4 Hz, 1H, CH_2_=CH_2_), 4.56 (dd, *J* = 2.5, 1.4 Hz, 1H, CH_2_=CH_2_), 4.55–4.52 (m, 1H, CH), 4.45 (d, *J* = 3.1 Hz, 2H, CH_2_), 2.37 (dt, *J* = 11.1, 5.5 Hz, 1H), 1.91 (ddd, *J* = 11.2, 7.0, 2.7 Hz, 1H), 1.68 (s, 3H, CH_3_), 1.66–1.59 (m, 6H), 1.50–1.46 (m, 2H), 1.42–1.37 (m, 6H), 1.36 (d, *J* = 4.3 Hz, 1H), 1.34 (d, *J* = 2.8 Hz, 1H), 1.29 (ddd, *J* = 21.6, 11.7, 7.4 Hz, 2H), 1.24–1.08 (m, 3H), 1.06 (dd, *J* = 12.8, 4.7 Hz, 1H), 1.01 (s, 3H, CH_3_), 1.00–0.95 (m, 2H), 0.93 (s, 3H, CH_3_), 0.85 (s, 3H, CH_3_), 0.83 (s, 3H, CH_3_), 0.78 (s, 6H, CH_3_×2). ^13^C NMR (150 MHz, chloroform-*d*) *δ* 168.47 (C35), 166.11 (C33), 165.57 (C31), 151.09 (C20), 149.81 (C37), 146.78 (C40), 120.31 (C34), 118.85 (C38), 118.31 (C36), 113.38 (C39), 109.52 (C29), 83.41 (C3), 55.43 (C5), 50.40 (C9), 48.39 (C18), 48.13 (C19), 43.12 (C17), 42.96 (C14), 42.41 (C32), 40.95 (C8), 40.12 (C22), 38.38 (C13), 38.14 (C1), 37.95 (C4), 37.15 (C10), 35.68 (C16), 34.27 (C7), 29.94 (C21), 28.03 (C15), 27.54 (C12), 25.17 (C2), 23.68 (C23), 21.06 (C24), 19.40 (C11), 18.25 (C30), 18.13 (C28), 16.52 (C6), 16.23 (C25), 16.08 (C26), 14.65 (C27). HRMS (ESI) *m*/*z*: calcd for C_40_H_55_NO_5_NaS [M+Na]^+^ 684.3699, found 684.3698.

##### Lupeol-3-[(1-Ethyl)-2]-5-(2-Thienylmethylene)-2,4-Thiazolidinedione (**6h**)

Yellow solid, yield: 64%; mp: 177.6–179.5 °C. ^1^H NMR (600 MHz, chloroform-*d*) *δ* 8.10 (s, 1H, CH_2_=CH_2_), 7.68 (d, *J* = 5.0 Hz, 1H, CH_2_=CH_2_), 7.42 (d, *J* = 3.8 Hz, 1H, CH_2_=CH_2_), 7.20 (t, *J* = 4.4 Hz, 1H, CH_2_=CH_2_), 4.68 (d, *J* = 2.4 Hz, 1H, CH_2_=CH_2_), 4.56 (s, 1H, CH_2_=CH_2_), 4.54 (d, *J* = 4.7 Hz, 1H, CH), 4.46 (d, *J* = 2.3 Hz, 2H, CH_2_), 2.37 (q, *J* = 5.3 Hz, 1H), 1.93–1.88 (m, 1H), 1.68 (s, 3H, CH_3_), 1.65 (d, *J* = 10.4 Hz, 6H), 1.49–1.46 (m, 2H), 1.40–1.36 (m, 6H), 1.36 (s, 1H), 1.34 (d, *J* = 2.8 Hz, 1H), 1.31–1.25 (m, 2H), 1.24–1.16 (m, 3H), 1.08–1.05 (m, 1H), 1.01 (s, 3H, CH_3_), 0.98–0.95 (m, 2H), 0.93 (s, 3H, CH_3_), 0.85 (s, 3H, CH_3_), 0.83 (s, 3H, CH_3_), 0.78 (s, 6H, CH_3_×2). ^13^C NMR (150 MHz, chloroform-*d*) *δ* 167.04 (C35), 166.04 (C33), 165.52 (C31), 151.73 (C20), 137.66 (C37), 133.81 (C40), 132.40 (C34), 128.83 (C38), 127.21 (C36), 119.47 (C39), 109.91 (C29), 83.49 (C3), 55.43 (C5), 50.40 (C9), 48.39 (C18), 48.13 (C19), 43.11 (C17), 42.96 (C14), 42.62 (C32), 40.95 (C8), 40.12 (C22), 38.39 (C13), 38.14 (C1), 37.96 (C4), 37.15 (C10), 35.68 (C16), 34.27 (C7), 29.94 (C21), 28.04 (C15), 27.54 (C12), 25.17 (C2), 23.68 (C23), 21.45 (C24), 19.85 (C11), 18.26 (C30), 18.13 (C28), 16.51 (C6), 16.24 (C25), 16.08 (C26), 14.65 (C27). HRMS (ESI) *m*/*z*: calcd for C_40_H_55_NO_4_NaS_2_ [M+Na]^+^ 700.3470, found 700.3479.

##### Lupeol-3-[(1-Ethyl)-2]-5-(2-Methylpropylidene)-2,4-Thiazolidinedione (**6i**)

White solid, yield: 45%; mp: 176.5–178.4 °C. ^1^H NMR (600 MHz, chloroform-*d*) *δ* 6.97 (d, *J* = 9.7 Hz, 1H, CH_2_=CH_2_), 4.68 (d, *J* = 2.5 Hz, 1H, CH_2_=CH_2_), 4.56 (s, 1H, CH_2_=CH_2_), 4.52 (dd, *J* = 11.5, 4.7 Hz, 1H, CH), 4.40 (d, *J* = 4.1 Hz, 2H, CH_2_), 2.46–2.41 (m, 1H), 2.38 (dt, *J* = 11.1, 5.4 Hz, 1H), 1.94–1.88 (m, 1H), 1.68 (s, 3H, CH_3_), 1.67–1.60 (m, 6H), 1.58 (d, *J* = 18.1 Hz, 1H), 1.50–1.46 (m, 2H), 1.41–1.37 (m, 6H, CH_3_×2), 1.36 (s, 1H), 1.34 (d, *J* = 2.2 Hz, 1H), 1.25 (s, 2H), 1.24–1.17 (m, 3H), 1.14 (dd, *J* = 6.6, 1.7 Hz, 6H), 1.06 (dd, *J* = 12.8, 4.6 Hz, 1H), 1.02 (s, 3H, CH_3_), 1.01–0.96 (m, 2H), 0.93 (s, 3H, CH_3_), 0.84 (d, *J* = 2.7 Hz, 6H, CH_3_×2), 0.78 (s, 6H, CH_3_×2). ^13^C NMR (150 MHz, chloroform-*d*) *δ* 168.36 (C35), 166.10 (C31), 165.45 (C33), 152.03 (C20), 145.50 (C36), 123.54 (C34), 109.52 (C29), 83.46 (C3), 55.44 (C5), 50.41 (C9), 48.39 (C18), 48.13 (C19), 43.12 (C17), 42.97 (C14), 42.31 (C32), 40.95 (C8), 40.12 (C22), 38.38 (C13), 38.14 (C1), 37.93 (C4), 37.16 (C10), 35.69 (C16), 34.28 (C7), 32.09 (C37), 29.94 (C21), 29.84 (C15), 28.03 (C38), 27.55 (C12), 25.18 (C2), 23.67 (C23), 21.36 (C24), 21.07 (C39), 19.41 (C11), 18.27 (C30), 18.13 (C28), 16.46 (C6), 16.24 (C25), 16.09 (C26), 14.66 (C27). HRMS (ESI) *m*/*z*: calcd for C_39_H_59_NO_4_NaS [M+Na]^+^ 660.4062, found 660.4068.

#### 3.1.6. Synthesis of Lupeol-3-(4-Nitrobenzoate) (**7**)

Lupeol (1, 500 mg, 1.17 mmol) and 4-nitrophenyl chloroformate (471 mg, 2.34 mmol) were reacted in CH_2_Cl_2_ (10 mL) under a N_2_ environment, after which pyridine (277 μL, 3.51 mmol) was added at 0 °C. The mixture was stirred for 30 min at 0 °C. Then, the reaction was continued at room temperature for 30–90 min. The reaction’s progress was monitored using TLC until there was no starting material left. The solvent was washed with distilled water twice and then saturated in a NaCl solution, dried over Na_2_SO_4_, and concentrated under reduced pressure. Crude product 7 was purified via column chromatography (CH_2_Cl_2_).

##### Lupeol-3-(4-Nitrobenzoate) (**7**)

White solid, yield: 80%; mp: 195.9–197.7 °C. ^1^H NMR (600 MHz, chloroform-*d*) *δ* 8.27 (d, *J* = 9.2 Hz, 2H, Ar-H), 7.38 (d, *J* = 9.1 Hz, 2H, Ar-H), 4.69 (d, *J* = 2.4 Hz, 1H, CH_2_=CH_2_), 4.57 (dd, *J* = 2.6, 1.4 Hz, 1H, CH_2_=CH_2_), 4.44 (dd, *J* = 11.4, 5.1 Hz, 1H), 2.38 (td, *J* = 11.0, 5.8 Hz, 1H), 1.92 (ddt, *J* = 13.8, 10.4, 5.2 Hz, 1H), 1.88–1.70 (m, 4H), 1.68 (s, 3H, CH_3_), 1.68–1.62 (m, 2H), 1.54–1.47 (m, 2H), 1.46–1.38 (m, 6H), 1.36 (s, 1H), 1.34 (d, *J* = 3.0 Hz, 1H), 1.30 (d, *J* = 2.8 Hz, 1H), 1.29–1.11 (m, 3H), 1.08 (dd, *J* = 12.7, 4.7 Hz, 1H), 1.04 (s, 3H, CH_3_), 1.02–1.00 (m, 1H), 0.99 (s, 3H, CH_3_), 0.95 (s, 3H, CH_3_), 0.89 (d, *J* = 8.2 Hz, 6H, CH_3_×2), 0.82 (dd, *J* = 9.1, 2.8 Hz, 1H), 0.79 (s, 3H, CH_3_). ^13^C NMR (150 MHz, chloroform-*d*) *δ* 155.92 (C32), 152.57 (C31), 151.08 (C22), 145.37 (C35), 125.39 (C29), 121.99 (C33, C37), 109.53 (C34, C36), 87.78 (C3), 55.48 (C5), 50.46 (C9), 48.40 (C18), 48.12 (C19), 43.12 (C17), 42.98 (C14), 40.97 (C20), 40.12 (C8), 38.43 (C13), 38.30 (C1), 38.14 (C4), 37.20 (C10), 35.68 (C16), 34.28 (C7), 29.95 (C21), 28.09 (C15), 27.56 (C12), 25.18 (C2), 23.68 (C23), 21.10 (C24), 19.42 (C11), 18.27 (C30), 18.13 (C28), 16.52 (C6), 16.31 (C25), 16.11 (C26), 14.66 (C27).

#### 3.1.7. Synthesis of Lupeol-3-Piperazinecarboxylate (**8**)

A solution of compound **7** (430 mg, 0.73 mmol), piperazine (125 mg, 1.46 mmol), and Et_3_N (202 μL, 1.46 mmol) was reacted at room temperature for 4–5 h. The reaction’s progress was monitored using TLC until no starting material remained. The sample was then concentrated under reduced pressure to remove the excess solvent. The residue was purified using silica gel column chromatography (CH_2_Cl_2_/MeOH = 40:1, *v*/*v*) to produce intermediate **8**.

##### Lupeol-3-Piperazinecarboxylate (**8**)

White solid, yield: 75%; mp: 190.7–192.5 °C. ^1^H NMR (600 MHz, chloroform-*d*) *δ* 4.68 (d, *J* = 2.4 Hz, 1H, CH_2_=CH_2_), 4.57 (t, *J* = 2.0 Hz, 1H, CH_2_=CH_2_), 4.36 (dd, *J* = 11.8, 4.5 Hz, 1H), 3.46 (t, *J* = 5.2 Hz, 4H, CH_2_×2), 2.84 (s, 4H, CH_2_×2), 2.38 (td, *J* = 11.0, 5.8 Hz, 1H), 2.20–2.15 (m, 1H), 1.91 (ddt, *J* = 13.7, 10.5, 5.2 Hz, 1H), 1.71 (dd, *J* = 8.6, 4.5 Hz, 1H), 1.69–1.68 (m, 3H, CH_3_), 1.67–1.61 (m, 3H), 1.59 (dd, *J* = 12.6, 3.2 Hz, 1H), 1.52–1.46 (m, 2H), 1.43–1.33 (m, 8H), 1.33–1.27 (m, 2H), 1.25 (d, *J* = 5.4 Hz, 1H), 1.23–1.16 (m, 2H), 1.07 (dd, *J* = 12.9, 4.6 Hz, 1H), 1.03 (s, 3H, CH_3_), 1.00 (dt, *J* = 13.4, 3.4 Hz, 2H), 0.94 (s, 3H, CH_3_), 0.88 (s, 3H, CH_3_), 0.84 (d, *J* = 12.4 Hz, 6H, CH_3_×2), 0.79 (s, 3H, CH_3_). ^13^C NMR (150 MHz, chloroform-*d*) *δ* 155.72 (C31), 151.12 (C22), 109.50 (C29), 82.12 (C3), 55.48 (C5), 50.42 (C9), 48.40 (C18), 48.13 (C19), 45.94 (C32, C35), 43.12 (C17), 42.95 (C14), 40.96 (C26), 40.12 (C33, C34), 38.47 (C8), 38.26 (C22), 38.17 (C13), 37.18 (C4), 35.70 (C1), 34.32 (C10), 29.94 (C16), 28.19 (C7), 27.56 (C21), 25.21 (C15), 24.30 (C12), 21.07 (C2), 19.40 (C23, C24), 18.34 (C11), 18.13 (C30), 16.96 (C28), 16.27 (C6), 16.10 (C25), 14.67 (C27).

#### 3.1.8. Synthesis of Compounds **9a**–**i**

A solution of compound **8** (50 mg, 0.09 mmol), intermediates **5a**–**i** (0.14 mmol), EDCI (26 mg, 0.14 mmol), and DMAP (6.10 mg, 0.05 mmol) in CH_2_Cl_2_ (6 mL) was stirred for 6–8 h. The reaction’s progress was monitored using TLC until no starting material remained. The solvent was then removed under reduced pressure to provide the crude product. Further purification involved separation via silica gel column chromatography (CH_2_Cl_2_/MeOH = 160:1, *v*/*v*) to produce compounds **9a**–**i**.

##### Lupeol-3-[(1-Piperazinyl)-4]-5-(4-Fluorobenzylidene)-2,4-Thiazolidinedione (**9a**)

White solid, yield: 55%; mp: 197.6–199.3 °C. ^1^H NMR (600 MHz, chloroform-*d*) *δ* 7.89 (s, 1H, CH_2_=CH_2_), 7.52 (dd, *J* = 8.7, 5.3 Hz, 2H, Ar-H), 7.18 (t, *J* = 8.5 Hz, 2H, Ar-H), 4.69 (d, *J* = 2.5 Hz, 1H, CH_2_=CH_2_), 4.58–4.56 (m, 1H, CH_2_=CH_2_), 4.55 (s, 2H, CH_2_), 4.40 (dd, *J* = 11.6, 4.4 Hz, 1H, CH), 3.56 (d, *J* = 62.4 Hz, 8H, CH_2_×4), 2.38 (td, *J* = 11.1, 5.8 Hz, 1H), 1.91 (ddd, *J* = 13.6, 10.9, 8.7 Hz, 1H), 1.69 (s, 3H, CH_3_), 1.68–1.53 (m, 6H), 1.52–1.47 (m, 2H), 1.46–1.38 (m, 6H), 1.37 (s, 1H), 1.35 (d, *J* = 5.7 Hz, 1H), 1.34–1.29 (m, 2H), 1.27–1.11 (m, 3H), 1.08 (dd, *J* = 12.9, 4.6 Hz, 1H), 1.03 (s, 3H, CH_3_), 1.03–0.98 (m, 2H), 0.95 (s, 3H, CH_3_), 0.89 (s, 3H, CH_3_), 0.86 (s, 3H, CH_3_), 0.84 (s, 3H, CH_3_), 0.79 (s, 3H, CH_3_). ^13^C NMR (150 MHz, chloroform-*d*) *δ* 167.79 (C36), 166.05 (C38), 164.72 (C40), 163.24 (C45), 163.03 (C31), 151.12 (C20), 133.39 (C41), 132.49 (C46), 132.43 (C43), 129.62 (C42), 121.13 (C39), 116.82 (C44), 116.67 (C46), 110.16 (C29), 82.85 (C3), 55.47 (C5), 50.44 (C9), 48.40 (C18), 48.14 (C19), 44.81 (C32, C33), 43.12 (C17), 42.96 (C14), 42.51 (C37), 42.27 (C34, C35), 40.97 (C8), 40.13 (C22), 38.45 (C13), 38.27 (C4), 38.16 (C1), 37.19 (C10), 35.69 (C16), 34.31 (C7), 29.95 (C21), 28.25 (C15), 27.56 (C12), 25.21 (C2), 24.27 (C23), 21.08 (C24), 19.41 (C11), 18.34 (C30), 18.13 (C28), 17.00 (C6), 16.28 (C25), 16.10 (C26), 15.04 (C27). HRMS (ESI) *m*/*z*: calcd for C_47_H_64_N_3_O_5_NaSF [M+Na]^+^ 824.4448, found 824.4444.

##### Lupeol-3-[(1-Piperazinyl)-4]-5-(4-Chlorobenzylidene)-2,4-Thiazolidinedione (**9b**)

Light-yellow solid, yield: 57%; mp: 186.8–188.6 °C. ^1^H NMR (600 MHz, chloroform-*d*) *δ* 7.87 (s, 1H, CH_2_=CH_2_), 7.45 (s, 4H, Ar-H), 4.69 (d, *J* = 2.5 Hz, 1H, CH_2_=CH_2_), 4.58–4.56 (m, 1H, CH_2_=CH_2_), 4.55 (s, 2H, CH_2_), 4.40 (dd, *J* = 11.7, 4.4 Hz, 1H, CH), 3.56 (d, *J* = 60.2 Hz, 8H, CH_2_×4), 2.38 (td, *J* = 11.0, 5.8 Hz, 1H), 1.95–1.88 (m, 1H), 1.69 (s, 3H, CH_3_), 1.63 (ddd, *J* = 20.1, 12.4, 3.8 Hz, 6H), 1.52–1.47 (m, 2H), 1.44–1.38 (m, 6H), 1.37 (s, 1H), 1.35 (d, *J* = 5.9 Hz, 1H), 1.34–1.27 (m, 2H), 1.27–1.11 (m, 3H), 1.08 (dd, *J* = 12.9, 4.6 Hz, 1H), 1.03 (s, 3H, CH_3_), 1.02–0.98 (m, 2H), 0.95 (s, 3H, CH_3_), 0.89 (s, 3H, CH_3_), 0.86 (s, 3H, CH_3_), 0.84 (s, 3H, CH_3_), 0.79 (s, 3H, CH_3_). ^13^C NMR (150 MHz, chloroform-*d*) *δ* 167.62 (C36), 165.97 (C38), 163.26 (C40), 155.44 (C45), 151.12 (C31), 136.92 (C20), 133.17 (C41), 131.76 (C42), 131.47 (C43, C47), 129.73 (C44, C46), 122.06 (C39), 109.50 (C29), 82.86 (C3), 55.46 (C5), 50.44 (C9), 48.40 (C18), 48.13 (C19), 44.80 (C32, C33), 43.12 (C17), 42.96 (C14), 42.53 (C37), 42.27 (C34, C35), 40.96 (C8), 40.12 (C22), 38.45 (C13), 38.26 (C4), 38.16 (C1), 37.19 (C10), 35.69 (C16), 34.30 (C7), 29.94 (C21), 28.25 (C15), 27.56 (C12), 25.20 (C2), 24.26 (C23), 21.08 (C24), 19.41 (C11), 18.34 (C30), 18.13 (C28), 16.99 (C6), 16.28 (C25), 16.10 (C26), 14.66 (C27). HRMS (ESI) *m*/*z*: calcd for C_47_H_64_N_3_O_5_NaSCl [M+Na]^+^ 840.4153, found 840.4153.

##### Lupeol-3-[(1-Piperazinyl)-2]-5-(4-Methylbenzylidene)-2,4-Thiazolidinedione (**9c**)

White solid, yield: 42%; mp: 191.8–193.6 °C. ^1^H NMR (600 MHz, chloroform-*d*) *δ* 7.90 (s, 1H, CH_2_=CH_2_), 7.41 (d, *J* = 8.0 Hz, 2H, Ar-H), 7.28 (d, *J* = 7.9 Hz, 2H, Ar-H), 4.69 (d, *J* = 2.5 Hz, 1H, CH_2_=CH_2_), 4.57 (t, *J* = 2.0 Hz, 1H, CH_2_=CH_2_), 4.55 (s, 2H, CH_2_), 4.40 (dd, *J* = 11.7, 4.4 Hz, 1H, CH), 3.63–3.48 (m, 8H, CH_2_×4), 2.41 (s, 3H, CH_3_), 2.37 (dt, *J* = 11.2, 5.6 Hz, 1H), 1.95–1.88 (m, 1H), 1.69 (d, *J* = 6.8 Hz, 3H, CH_3_), 1.68–1.59 (m, 6H), 1.52–1.46 (m, 2H), 1.46–1.37 (m, 6H), 1.37 (s, 1H), 1.35 (d, *J* = 5.7 Hz, 1H), 1.34–1.28 (m, 2H), 1.28–1.10 (m, 3H), 1.08 (dd, *J* = 12.9, 4.6 Hz, 1H), 1.03 (s, 3H, CH_3_), 1.02–0.97 (m, 2H), 0.95 (s, 3H, CH_3_), 0.89 (s, 3H, CH_3_), 0.86 (s, 3H, CH_3_), 0.84 (s, 3H, CH_3_), 0.79 (s, 3H, CH_3_). ^13^C NMR (150 MHz, chloroform-*d*) *δ* 168.20 (C36), 166.25 (C38), 163.36 (C40), 155.50 (C45), 151.11 (C31), 141.58 (C20), 134.83 (C41), 130.56 (C42), 130.48 (C43, C47), 130.15 (C44, C46), 120.16 (C39), 109.50 (C29), 82.82 (C3), 55.47 (C5), 50.43 (C9), 48.40 (C18), 48.13 (C19), 44.79 (C32, C33), 43.12 (C17), 42.96 (C14), 42.41 (C37), 42.24 (C34, C35), 40.96 (C8), 40.12 (C22), 38.45 (C13), 38.26 (C4), 38.16 (C1), 37.57 (C10), 35.69 (C16), 34.30 (C7), 29.94 (C21), 28.24 (C15), 27.56 (C12), 25.20 (C2), 24.26 (C23), 21.74 (C48), 21.08 (C24), 19.41 (C11), 18.33 (C30), 18.12 (C28), 16.99 (C6), 16.27 (C25), 16.09 (C26), 14.66 (C27). HRMS (ESI) *m*/*z*: calcd for C_48_H_67_N_3_O_5_NaS [M+Na]^+^ 820.4699, found 820.4700.

##### Lupeol-3-[(1-Piperazinyl)-4]-5-(4-Tert-Butylphenyl)-2,4-Thiazolidinedione (**9d**)

White solid, yield: 38%; mp: 187.4–189.2 °C. ^1^H NMR (600 MHz, chloroform-*d*) *δ* 7.91 (s, 1H, CH_2_=CH_2_), 7.50 (d, *J* = 8.2 Hz, 2H, Ar-H), 7.46 (d, *J* = 8.5 Hz, 2H, Ar-H), 4.69 (d, *J* = 2.6 Hz, 1H, CH_2_=CH_2_), 4.57 (t, *J* = 2.0 Hz, 1H, CH_2_=CH_2_), 4.55 (s, 2H, CH_2_), 4.40 (dd, *J* = 11.7, 4.4 Hz, 1H, CH), 3.56 (d, *J* = 64.3 Hz, 8H, CH_2_×4), 2.38 (td, *J* = 11.0, 5.8 Hz, 1H), 1.95–1.88 (m, 1H), 1.69 (s, 3H, CH_3_), 1.68–1.57 (m, 6H), 1.52–1.47 (m, 2H), 1.45–1.38 (m, 6H), 1.37–1.36 (m, 1H), 1.34 (s, 9H, CH_3_×3), 1.32–1.28 (m, 2H), 1.28–1.11 (m, 3H), 1.08 (dd, *J* = 13.0, 4.5 Hz, 1H), 1.03 (s, 3H, CH_3_), 1.02–0.99 (m, 2H), 0.95 (s, 3H, CH_3_), 0.89 (s, 3H, CH_3_), 0.86 (s, 3H, CH_3_), 0.84 (s, 3H, CH_3_), 0.79 (s, 3H, CH_3_). ^13^C NMR (150 MHz, chloroform-*d*) *δ* 168.23 (C36), 166.26 (C38), 163.38 (C40), 155.42 (C45), 154.60 (C31), 151.11 (C20), 134.70 (C41), 130.50 (C42), 130.39 (C43, C47), 126.42 (C44, C46), 120.27 (C39), 109.51 (C29), 82.82 (C3), 55.47 (C5), 50.43 (C9), 48.40 (C18), 48.13 (C19), 44.80 (C32, C33), 43.12 (C17), 42.96 (C14), 42.41 (C37), 42.24 (C34, C35), 40.96 (C8), 40.12 (C22), 38.45 (C13), 38.26 (C4), 38.16 (C1), 37.19 (C10), 35.69 (C16), 35.21 (C48), 34.31 (C7), 31.20 (C49, C50, C51), 29.95 (C21), 28.25 (C15), 27.56 (C12), 25.20 (C2), 24.26 (C23), 21.08 (C24), 19.41 (C11), 18.34 (C30), 18.13 (C28), 17.00 (C6), 16.27 (C25), 16.10 (C26), 14.66 (C27). HRMS (ESI) *m*/*z*: calcd for C_51_H_73_N_3_O_5_NaS [M+Na]^+^ 862.5169, found 862.5168.

##### Lupeol-3-[(1-Piperazinyl)-4]-5-(2-Methoxybenzylidene)-2,4-Thiazolidinedione (**9e**)

Light-yellow solid, yield: 45%; mp: 194.6–196.4 °C. ^1^H NMR (600 MHz, chloroform-*d*) *δ* 8.29 (s, 1H, CH_2_=CH_2_), 7.45 (dd, *J* = 7.8, 1.5 Hz, 1H, Ar-H), 7.43–7.39 (m, 1H, Ar-H), 7.04 (t, *J* = 7.5 Hz, 1H, Ar-H), 6.94 (d, *J* = 8.3 Hz, 1H, Ar-H), 4.69 (d, *J* = 2.6 Hz, 1H, CH_2_=CH_2_), 4.57 (t, *J* = 2.1 Hz, 1H, CH_2_=CH_2_), 4.55 (s, 2H, CH_2_), 4.40 (dd, *J* = 11.7, 4.5 Hz, 1H, CH), 3.89 (s, 3H, OCH_3_), 3.56 (d, *J* = 61.1 Hz, 8H, CH_2_×4), 2.38 (td, *J* = 11.0, 5.8 Hz, 1H), 1.95–1.88 (m, 1H), 1.69 (s, 3H, CH_3_), 1.68–1.58 (m, 6H), 1.52–1.46 (m, 2H), 1.46–1.38 (m, 6H), 1.37–1.36 (m, 1H), 1.35 (d, *J* = 5.6 Hz, 1H), 1.32–1.29 (m, 2H), 1.27–1.21 (m, 3H), 1.08 (dd, *J* = 12.9, 4.5 Hz, 1H), 1.03 (s, 3H, CH_3_), 1.02–0.98 (m, 2H), 0.95 (s, 3H, CH_3_), 0.89 (s, 3H, CH_3_), 0.86 (s, 3H, CH_3_), 0.84 (s, 3H, CH_3_), 0.79 (s, 3H, CH_3_). ^13^C NMR (150 MHz, chloroform-*d*) *δ* 168.61 (C36), 166.22 (C38), 163.49 (C40), 158.65 (C45), 151.11 (C31), 132.50 (C20), 130.58 (C42), 129.57 (C41), 122.49 (C43), 121.74 (C44), 121.01 (C47), 111.27 (C46), 110.35 (C39), 109.50 (C29), 82.81 (C3), 55.63 (C5), 55.47 (C9), 48.40 (C18), 48.13 (C19), 44.80 (C32, C33), 43.12 (C17), 42.96 (C14), 42.31 (C37), 42.22 (C34, C35), 40.96 (C8), 40.12 (C22), 38.45 (C13), 38.26 (C4), 38.16 (C1), 37.18 (C10), 35.69 (C16), 34.30 (C7), 29.94 (C21), 28.24 (C15), 27.56 (C12), 25.20 (C2), 24.26 (C23), 22.78 (C48), 21.08 (C24), 19.41 (C11), 18.33 (C30), 18.12 (C28), 16.99 (C6), 16.27 (C25), 16.09 (C26), 14.66 (C27). HRMS (ESI) *m*/*z*: calcd for C_48_H_67_N_3_O_6_NaS [M+Na]^+^ 836.4648, found 836.4649.

##### Lupeol-3-[(1-Piperazinyl)-4]-5-(2,3,4-Trimethoxybenzylidene)-2,4-Thiazolidinedione (**9f**)

Yellow solid, yield: 62%; mp: 195.8–197.6 °C. ^1^H NMR (600 MHz, chloroform-*d*) *δ* 8.18 (s, 1H, CH_2_=CH_2_), 7.21 (d, *J* = 8.8 Hz, 1H, Ar-H), 6.78 (d, *J* = 8.8 Hz, 1H, Ar-H), 4.69 (d, *J* = 2.6 Hz, 1H, CH_2_=CH_2_), 4.57 (t, *J* = 2.0 Hz, 1H, CH_2_=CH_2_), 4.55 (s, 2H, CH_2_), 4.40 (dd, *J* = 11.6, 4.4 Hz, 1H, CH), 3.93 (d, *J* = 8.2 Hz, 6H, OCH_3_×2), 3.88 (s, 3H, OCH_3_), 3.56 (d, *J* = 61.1 Hz, 8H, CH_2_×4), 2.38 (td, *J* = 11.1, 5.8 Hz, 1H), 1.95–1.88 (m, 1H), 1.69 (s, 3H, CH_3_), 1.68–1.54 (m, 6H), 1.52–1.47 (m, 2H), 1.46–1.38 (m, 6H), 1.37 (s, 1H), 1.35 (d, *J* = 5.5 Hz, 1H), 1.31 (dd, *J* = 12.7, 2.7 Hz, 2H), 1.27–1.10 (m, 3H), 1.08 (dd, *J* = 12.9, 4.5 Hz, 1H), 1.03 (s, 3H, CH_3_), 1.03–0.98 (m, 2H), 0.95 (s, 3H, CH_3_), 0.89 (s, 3H, CH_3_), 0.86 (s, 3H, CH_3_), 0.84 (s, 3H, CH_3_), 0.79 (s, 3H, CH_3_). ^13^C NMR (150 MHz, chloroform-*d*) *δ* 168.60 (C36), 166.27 (C38), 163.65 (C40), 156.43 (C45), 155.54 (C31), 154.06 (C20), 151.12 (C41), 142.94 (C42), 130.04 (C43), 124.78 (C44), 120.50 (C47), 119.55 (C46), 109.50 (C39), 107.72 (C29), 82.82 (C3), 62.05 (C48), 61.09 (C49), 56.28 (C50), 55.47 (C5), 50.43 (C9), 48.40 (C18), 48.13 (C19), 44.81 (C32, C33), 43.12 (C17), 42.96 (C14), 42.31 (C37), 42.23 (C34, C35), 40.96 (C8), 40.12 (C22), 38.45 (C13), 38.26 (C4), 38.16 (C1), 37.19 (C10), 35.69 (C16), 34.30 (C7), 29.94 (C21), 28.24 (C15), 27.56 (C12), 25.20 (C2), 24.26 (C23), 21.08 (C24), 19.40 (C11), 18.33 (C30), 18.12 (C28), 16.99 (C6), 16.27 (C25), 16.09 (C26), 14.66 (C27). HRMS (ESI) *m*/*z*: calcd for C_50_H_71_N_3_O_8_NaS [M+Na]^+^ 896.4860, found 896.4853.

##### Lupeol-3-[(1-Piperazinyl)-4]-5-(2-Furanylmethylene)-2,4-Thiazolidinedione (**9g**)

Light-brown solid, yield: 57%; mp: 189.3–191.2 °C. ^1^H NMR (600 MHz, chloroform-*d*) *δ* 7.68 (d, *J* = 1.8 Hz, 1H, CH_2_=CH_2_), 7.66 (s, 1H, CH_2_=CH_2_), 6.80 (d, *J* = 3.5 Hz, 1H, CH_2_=CH_2_), 6.58 (dd, *J* = 3.6, 1.8 Hz, 1H, CH_2_=CH_2_), 4.69 (d, *J* = 2.5 Hz, 1H, CH_2_=CH_2_), 4.57 (d, *J* = 2.6 Hz, 1H, CH_2_=CH_2_), 4.53 (s, 2H, CH_2_), 4.39 (dd, *J* = 11.7, 4.4 Hz, 1H, CH), 3.55 (d, *J* = 63.0 Hz, 8H, CH_2_×4), 2.38 (td, *J* = 11.1, 5.8 Hz, 1H), 1.95–1.88 (m, 1H), 1.69 (s, 3H, CH_3_), 1.68–1.58 (m, 6H), 1.52–1.46 (m, 2H), 1.46–1.37 (m, 6H), 1.37 (s, 1H), 1.35 (d, *J* = 5.7 Hz, 1H), 1.33–1.27 (m, 2H), 1.27–1.11 (m, 3H), 1.08 (dd, *J* = 12.9, 4.6 Hz, 1H), 1.03 (s, 3H, CH_3_), 1.01 (dd, *J* = 13.7, 3.7 Hz, 2H), 0.95 (s, 3H, CH_3_), 0.89 (s, 3H, CH_3_), 0.86 (s, 3H, CH_3_), 0.84 (s, 3H, CH_3_), 0.79 (s, 3H, CH_3_). ^13^C NMR (150 MHz, chloroform-*d*) *δ* 168.93 (C36), 165.96 (C38), 163.40 (C40), 155.60 (C45), 151.11 (C31), 149.85 (C20), 146.73 (C41), 120.82 (C43), 119.06 (C42), 118.19 (C39), 113.33 (C44), 109.50 (C29), 82.81 (C3), 55.47 (C5), 50.43 (C9), 48.40 (C18), 48.13 (C19), 44.78 (C32, C33), 43.12 (C17), 42.96 (C14), 42.30 (C37), 42.21 (C34, C35), 40.96 (C8), 40.12 (C22), 38.44 (C13), 38.25 (C4), 38.15 (C1), 37.18 (C10), 35.68 (C16), 34.30 (C7), 29.94 (C21), 28.24 (C15), 27.56 (C12), 25.20 (C2), 24.25 (C23), 21.07 (C24), 19.40 (C11), 18.33 (C30), 18.12 (C28), 16.99 (C6), 16.27 (C25), 16.09 (C26), 14.66 (C27). HRMS (ESI) *m*/*z*: calcd for C_45_H_63_N_3_O_6_NaS [M+Na]^+^ 796.4335, found 796.4327.

##### Lupeol-3-[(1-Piperazinyl)-4]-5-(2-Thienylmethylene)-2,4-Thiazolidinedione (**9h**)

Light-yellow solid, yield: 53%; mp: 191.7–193.4 °C. ^1^H NMR (600 MHz, chloroform-*d*) *δ* 8.09 (s, 1H, CH_2_=CH_2_), 7.67 (d, *J* = 5.0 Hz, 1H, CH_2_=CH_2_), 7.41 (d, *J* = 3.7 Hz, 1H, CH_2_=CH_2_), 7.20–7.18 (m, 1H, CH_2_=CH_2_), 4.69 (d, *J* = 2.5 Hz, 1H, CH_2_=CH_2_), 4.57 (t, *J* = 2.0 Hz, 1H, CH_2_=CH_2_), 4.54 (s, 2H, CH_2_), 4.40 (dd, *J* = 11.7, 4.4 Hz, 1H, CH), 3.55 (d, *J* = 61.5 Hz, 8H, CH_2_×4), 2.38 (td, *J* = 11.0, 5.8 Hz, 1H), 1.91 (ddd, *J* = 13.1, 7.1, 2.9 Hz, 1H), 1.69 (s, 3H, CH_3_), 1.68–1.59 (m, 6H), 1.52–1.47 (m, 2H), 1.43–1.38 (m, 6H), 1.37 (s, 1H), 1.35 (d, *J* = 5.7 Hz, 1H), 1.33–1.26 (m, 2H), 1.26–1.17 (m, 3H), 1.08 (dd, *J* = 12.9, 4.6 Hz, 1H), 1.03 (s, 3H, CH_3_), 1.00 (d, *J* = 3.6 Hz, 2H), 0.95 (s, 3H, CH_3_), 0.89 (s, 3H, CH_3_), 0.86 (s, 3H, CH_3_), 0.84 (s, 3H, CH_3_), 0.79 (s, 3H, CH_3_). ^13^C NMR (150 MHz, chloroform-*d*) *δ* 167.52 (C36), 165.91 (C38), 163.51 (C40), 155.56 (C45), 151.11 (C31), 137.71 (C20), 133.69 (C41), 132.30 (C43), 128.79 (C42), 127.20 (C39), 119.28 (C44), 109.50 (C29), 82.83 (C3), 55.47 (C5), 50.43 (C9), 48.40 (C18), 48.13 (C19), 44.79 (C32, C33), 43.12 (C17), 42.96 (C14), 42.55 (C37), 42.24 (C34, C35), 40.96 (C8), 40.12 (C22), 38.45 (C13), 38.26 (C4), 38.16 (C1), 37.18 (C10), 35.69 (C16), 34.30 (C7), 29.94 (C21), 28.24 (C15), 27.56 (C12), 25.20 (C2), 24.26 (C23), 21.08 (C24), 19.41 (C11), 18.33 (C30), 18.12 (C28), 16.99 (C6), 16.27 (C25), 16.09 (C26), 14.66 (C27). HRMS (ESI) *m*/*z*: calcd for C_45_H_63_N_3_O_5_NaS_2_ [M+Na]^+^ 812.4107, found 812.4107.

##### Lupeol-3-[(1-Piperazinyl)-4]-5-(2-Methylpropylidene)-2,4-Thiazolidinedione (**9i**)

White solid, yield: 48%; mp: 187.6–189.5 °C. ^1^H NMR (600 MHz, chloroform-*d*) *δ* 6.96 (d, *J* = 9.7 Hz, 1H, CH_2_=CH_2_), 4.69 (d, *J* = 2.6 Hz, 1H, CH_2_=CH_2_), 4.57 (t, *J* = 2.0 Hz, 1H, CH_2_=CH_2_), 4.48 (s, 2H, CH_2_), 4.39 (dd, *J* = 11.7, 4.5 Hz, 1H, CH), 3.54 (d, *J* = 65.5 Hz, 8H, CH_2_×4), 2.47–2.42 (m, 1H), 2.38 (td, *J* = 11.0, 5.8 Hz, 1H), 1.91 (ddd, *J* = 13.2, 6.9, 2.7 Hz, 1H), 1.68 (s, 3H, CH_3_), 1.68–1.53 (m, 6H), 1.52–1.46 (m, 2H), 1.43–1.37 (m, 6H), 1.37 (d, *J* = 2.7 Hz, 1H), 1.35 (d, *J* = 5.7 Hz, 1H), 1.33–1.29 (m, 2H), 1.22 (ddd, *J* = 18.2, 14.3, 10.7 Hz, 3H), 1.14 (d, *J* = 6.7 Hz, 6H, CH_3_×2), 1.08 (dd, *J* = 12.9, 4.5 Hz, 1H), 1.03 (s, 3H, CH_3_), 1.02–0.98 (m, 2H), 0.95 (s, 3H, CH_3_), 0.89 (s, 3H, CH_3_), 0.86 (s, 3H, CH_3_), 0.84 (s, 3H, CH_3_), 0.79 (s, 3H, CH_3_). ^13^C NMR (150 MHz, chloroform-*d*) *δ* 167.98 (C36), 165.19 (C38), 163.64 (C40), 155.55 (C31), 151.12 (C20), 145.43 (C41), 122.96 (C39), 109.50 (C29), 82.83 (C3), 55.47 (C5), 50.44 (C9), 48.40 (C18), 48.14 (C19), 44.77 (C32, C33), 43.48 (C17), 43.12 (C14), 42.96 (C37), 42.19 (C34, C35), 40.96 (C8), 40.13 (C22), 38.45 (C13), 38.26 (C4), 38.16 (C1), 37.19 (C10), 35.69 (C16), 34.31 (C7), 32.09 (C42), 29.95 (C21), 28.24 (C15), 27.56 (C12), 25.21 (C2), 24.26 (C23), 21.37 (C43, C44), 21.08 (C24), 19.41 (C11), 18.34 (C30), 18.13 (C28), 16.99 (C6), 16.27 (C25), 16.10 (C26), 14.66 (C27). HRMS (ESI) *m*/*z*: calcd for C_44_H_67_N_3_O_5_NaS [M+Na]^+^ 772.4699, found 772.4700.

#### 3.1.9. Synthesis of Lupeol-3-[(1-Ethylenediaminyl)-4]-Tert-Butoxycarbonyl (**10**)

Compound **7** (500 mg, 0.85 mmol) and N-Boc-ethylenediamine (408 μL, 2.55 mmol) were reacted in the presence of Et_3_N (354 μL, 2.55 mmol) for 1–2 h. The reaction was monitored via TLC until the raw material was completely reacted. Crude compound **10** was purified using silica gel chromatography (CH_2_Cl_2_/MeOH = 62:1, *v*/*v*); this produced the target product, namely **10**.

##### Lupeol-3-[(1-Ethylenediaminyl)-4]-Tert-Butoxycarbonyl (**10**)

White solid, yield: 79%; mp: 185.8–187.7 °C. ^1^H NMR (600 MHz, chloroform-*d*) *δ* 5.04 (d, *J* = 5.8 Hz, 1H, NH), 4.92 (s, 1H, NH), 4.68 (d, *J* = 2.5 Hz, 1H, CH), 4.57 (s, 1H, CH_2_=CH_2_), 4.33 (dd, *J* = 12.1, 4.2 Hz, 1H, CH_2_=CH_2_), 3.27 (d, *J* = 15.7 Hz, 4H, CH_2_×2), 2.38 (td, *J* = 11.1, 5.8 Hz, 1H), 1.91 (ddd, *J* = 13.2, 7.2, 3.0 Hz, 1H), 1.68 (s, 3H, CH_3_), 1.65 (d, *J* = 3.3 Hz, 2H), 1.52–1.45 (m, 5H), 1.44 (d, *J* = 3.7 Hz, 9H), 1.40–1.35 (m, 7H), 1.31–1.24 (m, 5H), 1.23–1.17 (m, 3H), 1.07 (dd, *J* = 12.9, 4.6 Hz, 1H), 1.03 (s, 3H, CH_3_), 0.98 (d, *J* = 4.1 Hz, 1H), 0.94 (s, 3H, CH_3_), 0.87 (s, 3H, CH_3_), 0.84 (s, 3H, CH_3_), 0.79 (s, 6H, CH_3_×2). ^13^C NMR (150 MHz, chloroform-*d*) *δ* 151.09 (C34), 109.48 (C31), 81.59 (C22), 79.59 (C29), 55.52 (C3), 50.42 (C35), 48.38 (C5), 48.11 (C9), 43.10 (C18), 42.93 (C19), 41.35 (C17), 40.94 (C14), 40.88 (C8), 40.10 (C32), 38.47 (C21), 38.14 (C33), 37.15 (C13), 35.67 (C4), 34.31 (C1), 31.70 (C10), 29.92 (C16), 29.81 (C7), 28.49 (C38, C37, C36), 28.05 (C20), 27.53 (C15), 25.19 (C12), 24.20 (C2), 22.77 (C23), 21.04 (C11), 19.39 (C24), 18.29 (C30), 18.11 (C28), 16.61 (C6), 16.27 (C25), 16.08 (C26), 14.63 (C27).

#### 3.1.10. Synthesis of Intermediate **11**

Excess CF_3_COOH was added dropwise to a stirred solution of intermediate **10** in CH_2_Cl_2_ (8 mL) under nitrogen at 0 °C and monitored using TLC. After the reaction, the solvent was removed under reduced pressure to produce light-yellow oil **11**, which was used in the next step without further purification.

#### 3.1.11. Synthesis of Compounds **12a**–**i**

EDCI (0.80 equiv), HOBT (0.10 equiv), and DIPEA (1.50 equiv) were added to a stirred solution of intermediate **5a**–**i** (70 mg, 0.20–0.31 mmol) in CH_2_Cl_2_ (8 mL) at 0 °C. After 0.5 h, intermediate **11** (0.50 equiv) was added, and the reaction mixture was stirred for 8–12 h at room temperature. The mixtures were concentrated. The residue was purified using silica gel chromatography (CH_2_Cl_2_/MeOH = 80:1, *v*/*v*). This process produced target products in the form of compounds **12a**–**i**.

##### Lupeol-3-[(1-Ethylenediaminyl)-4]-5-(4-Fluorobenzylidene)-2,4-Thiazolidinedione (**12a**)

White solid, yield: 32%; mp: 176.7–178.6 °C. ^1^H NMR (600 MHz, chloroform-*d*) *δ* 7.88 (s, 1H, CH_2_=CH_2_), 7.52 (dd, *J* = 8.8, 5.1 Hz, 2H, Ar-H), 7.17 (t, *J* = 8.3 Hz, 2H, Ar-H), 7.13 (d, *J* = 5.3 Hz, 1H, NH), 5.26 (d, *J* = 6.9 Hz, 1H, CH_2_=CH_2_), 5.19 (t, *J* = 6.1 Hz, 1H, CH_2_=CH_2_), 4.39 (s, 2H, CH_2_), 4.33–4.26 (m, 1H, CH), 3.48–3.25 (m, 4H, CH_2_×2, 1H, NH), 1.78–1.70 (m, 3H), 1.70–1.64 (m, 3H, CH_3_), 1.64 (s, 4H), 1.58 (d, *J* = 8.5 Hz, 1H), 1.56–1.53 (m, 1H), 1.49–1.46 (m, 2H), 1.40 (d, *J* = 3.6 Hz, 1H), 1.35 (t, *J* = 5.1 Hz, 2H), 1.32 (dd, *J* = 13.4, 4.3 Hz, 2H), 1.25 (d, *J* = 6.4 Hz, 3H), 1.23–1.17 (m, 3H), 1.06 (s, 1H), 1.03 (s, 1H), 1.00 (d, *J* = 6.9 Hz, 2H), 0.94 (s, 3H, CH_3_), 0.86 (t, *J* = 11.5 Hz, 9H, CH_3_×3), 0.79 (s, 3H, CH_3_), 0.73 (s, 3H, CH_3_). ^13^C NMR (150 MHz, chloroform-*d*) *δ* 167.58 (C34), 165.90 (C36), 165.71 (C38), 164.69 (C43), 163.00 (C31), 158.49 (C22), 139.97 (C39), 133.22 (C45), 132.47 (C41), 132.41 (C40), 130.23 (C37), 119.01 (C42), 116.80 (C44), 116.66 (C29), 82.29 (C3), 55.57 (C5), 50.44 (C9), 48.80 (C18), 43.99 (C19), 42.43 (C17), 42.28 (C14), 41.16 (C35), 39.29 (C8), 38.54 (C32), 38.13 (C21), 37.07 (C33), 36.79 (C13), 36.42 (C4), 34.49 (C1), 34.23 (C10), 29.82 (C16), 28.13 (C7), 27.71 (C20), 27.14 (C15), 24.15 (C12), 22.65 (C2), 21.75 (C23), 21.72 (C11), 18.26 (C24), 17.82 (C30), 16.67 (C28), 16.42 (C6), 16.13 (C25), 14.88 (C26), 14.25 (C27). HRMS (ESI) *m*/*z*: calcd for C_45_H_62_N_3_O_5_NaSF [M+Na]^+^ 798.4292, found 798.4301.

##### Lupeol-3-[(1-Ethylenediaminyl)-4]-5-(4-Chlorobenzylidene)-2,4-Thiazolidinedione (**12b**)

White solid, yield: 37%; mp: 182.9–184.8 °C. ^1^H NMR (600 MHz, chloroform-*d*) *δ* 7.86 (s, 1H, CH_2_=CH_2_), 7.45 (s, 4H, Ar-H), 6.96 (s, 1H), 5.04 (s, 1H, NH), 4.70 (s, 1H, CH_2_=CH_2_), 4.58 (s, 1H, CH_2_=CH_2_), 4.38 (s, 2H, CH_2_), 4.31–4.23 (m, 1H, CH), 3.37 (d, *J* = 45.4 Hz, 4H, CH_2_×2), 2.38 (td, *J* = 11.0, 5.8 Hz, 1H), 1.93 (q, *J* = 11.1, 10.7 Hz, 1H), 1.70 (s, 3H, CH_3_), 1.69–1.58 (m, 6H), 1.50–1.46 (m, 2H), 1.38 (d, *J* = 10.0 Hz, 6H), 1.32 (s, 1H), 1.31–1.23 (m, 3H), 1.23–1.15 (m, 3H), 1.07 (d, *J* = 11.8 Hz, 1H), 1.02 (s, 3H), 0.99 (s, 1H), 0.94 (s, 3H, CH_3_), 0.84 (d, *J* = 19.4 Hz, 6H, CH_3_×2), 0.78 (d, *J* = 5.5 Hz, 6H, CH_3_×2), 0.72 (d, *J* = 9.0 Hz, 1H). ^13^C NMR (150 MHz, chloroform-*d*) *δ* 167.41 (C34), 165.81 (C36), 165.53 (C38), 158.84 (C43), 151.11 (C31), 136.95 (C22), 133.01 (C39), 131.80 (C37), 131.44 (C41, C45), 129.74 (C42, C44), 122.19 (C44), 109.53 (C29), 82.43 (C3), 55.62 (C5), 50.49 (C9), 48.43 (C18), 48.13 (C19), 44.11 (C17), 43.13 (C14), 42.94 (C35), 42.23 (C8), 40.96 (C32), 40.31 (C21), 40.12 (C33), 38.52 (C13), 38.15 (C4), 37.16 (C1), 35.69 (C10), 34.29 (C16), 29.96 (C7), 29.84 (C20), 28.15 (C15), 27.56 (C12), 25.25 (C2), 24.16 (C23), 21.06 (C11), 19.47 (C24), 18.30 (C30), 18.13 (C28), 16.66 (C6), 16.26 (C25), 16.09 (C26), 14.71 (C27). HRMS (ESI) *m*/*z*: calcd for C_45_H_62_N_3_O_5_NaSCl [M+Na]^+^ 814.3996, found 814.4000.

##### Lupeol-3-[(1-Ethylenediaminyl)-2]-5-(4-Methylbenzylidene)-2,4-Thiazolidinedione (**12c**)

Light-brown solid, yield: 45%; mp: 192.4–194.2 °C. ^1^H NMR (600 MHz, chloroform-*d*) *δ* 7.88 (s, 1H, CH_2_=CH_2_), 7.40 (d, *J* = 7.8 Hz, 2H, Ar-H), 7.27 (d, *J* = 8.5 Hz, 2H, Ar-H), 7.16 (d, *J* = 5.0 Hz, 1H, NH), 5.25 (dd, *J* = 13.4, 5.6 Hz, 1H, CH_2_=CH_2_), 5.20 (d, *J* = 6.1 Hz, 1H, CH_2_=CH_2_), 4.39 (s, 2H, CH_2_), 4.30 (td, *J* = 9.6, 8.7, 4.7 Hz, 1H, CH), 3.43–3.27 (m, 4H, CH_2_×2, 1H, NH), 2.40 (d, *J* = 3.0 Hz, 3H, CH_3_), 2.02 (h, *J* = 6.4 Hz, 1H), 1.76 (ddd, *J* = 18.5, 11.5, 4.6 Hz, 2H), 1.69 (s, 1H), 1.64 (s, 3H, CH_3_), 1.57 (q, *J* = 8.8, 8.3 Hz, 2H), 1.48 (dt, *J* = 13.4, 8.8 Hz, 3H), 1.41 (t, *J* = 7.4 Hz, 3H), 1.35 (s, 1H), 1.31 (td, *J* = 7.9, 3.4 Hz, 3H), 1.25 (d, *J* = 4.6 Hz, 3H), 1.14 (s, 1H), 1.03 (d, *J* = 4.4 Hz, 3H), 1.00 (d, *J* = 5.3 Hz, 3H), 0.94 (s, 3H, CH_3_), 0.89–0.83 (m, 9H, CH_3_×3), 0.78 (d, *J* = 3.5 Hz, 3H, CH_3_), 0.73 (d, *J* = 6.4 Hz, 3H, CH_3_). ^13^C NMR (150 MHz, chloroform-*d*) *δ* 168.02 (C34), 166.04 (C36), 141.61 (C38), 139.95 (C43), 134.76 (C31), 134.40 (C22), 133.36 (C39), 130.46 (C41, C45), 130.14 (C42, C44), 126.21 (C37), 119.02 (C40), 115.78 (C29), 82.33 (C3), 55.55 (C5), 50.40 (C9), 48.81 (C18), 44.73 (C19), 43.94 (C17), 42.42 (C14), 42.27 (C35), 41.16 (C8), 40.31 (C32), 39.29 (C21), 38.52 (C33), 38.12 (C13), 37.06 (C4), 36.79 (C1), 36.42 (C10), 34.49 (C16), 34.22 (C7), 33.44 (C46), 32.47 (C20), 29.82 (C15), 28.11 (C12), 27.72 (C2), 27.14 (C23), 24.13 (C11), 22.68 (C24), 21.75 (C30), 18.26 (C28), 17.81 (C6), 16.42 (C25), 16.12 (C26), 14.87 (C27). HRMS (ESI) *m*/*z*: calcd for C_46_H_65_N_3_O_5_NaS [M+Na]^+^ 794.4543, found 794.4537.

##### Lupeol-3-[(1-Ethylenediaminyl)-4]-5-(4-Tert-Butylphenyl)-2,4-Thiazolidinedione (**12d**)

White solid, yield: 39%; mp: 184.6–186.3 °C. ^1^H NMR (600 MHz, chloroform-*d*) *δ* 7.89 (s, 1H, CH_2_=CH_2_), 7.50–7.43 (m, 4H, Ar-H), 7.23–7.15 (m, 1H, NH), 5.26 (d, *J* = 7.0 Hz, 2H, CH_2_=CH_2_), 4.40 (s, 2H, CH_2_), 4.33 (p, *J* = 6.2, 5.1 Hz, 1H, CH), 3.47–3.26 (m, 4H, CH_2_×2, 1H, NH), 2.02 (tt, *J* = 12.8, 6.5 Hz, 1H), 1.75 (s, 1H), 1.71–1.66 (m, 3H), 1.63 (s, 3H, CH_3_), 1.57–1.54 (m, 1H), 1.49 (ddd, *J* = 15.0, 10.6, 6.1 Hz, 3H), 1.44–1.37 (m, 5H), 1.33 (s, 9H, CH_3_×3), 1.29 (d, *J* = 4.1 Hz, 1H), 1.25 (s, 2H), 1.23–1.18 (m, 2H), 1.15 (s, 1H), 1.04 (d, *J* = 4.8 Hz, 2H), 0.99 (dd, *J* = 10.9, 4.4 Hz, 4H), 0.94 (d, *J* = 6.8 Hz, 3H), 0.89–0.83 (m, 9H, CH_3_×3), 0.79 (s, 3H, CH_3_), 0.72 (d, *J* = 20.0 Hz, 3H, CH_3_). ^13^C NMR (150 MHz, chloroform-*d*) *δ* 168.05 (C34), 166.16 (C36), 163.17 (C38), 158.40 (C43), 154.65 (C31), 139.93 (C22), 134.68 (C39), 130.39 (C41, C45), 126.39 (C42, C44), 120.13 (C40), 119.00 (C20), 115.76 (C29), 82.28 (C3), 55.55 (C37), 50.41 (C5), 48.78 (C9), 44.73 (C18), 43.88 (C17), 42.42 (C14), 41.15 (C35), 40.31 (C8), 39.29 (C32), 38.13 (C46), 37.07 (C33), 36.78 (C13), 36.41 (C4), 35.16 (C1), 34.47 (C10), 34.23 (C19), 33.42 (C7), 32.46 (C16), 31.16 (C47, C48, C49), 29.80 (C21), 28.08 (C15), 27.72 (C12), 27.12 (C2), 24.19 (C23), 22.66 (C11), 21.74 (C24), 18.25 (C30), 17.80 (C28),16.65 (C6), 16.42 (C25), 16.12 (C26), 14.87 (C27). HRMS (ESI) *m*/*z*: calcd for C_49_H_71_N_3_O_5_NaS [M+Na]^+^ 836.5012, found 836.5015.

##### Lupeol-3-[(1-Ethylenediaminyl)-4]-5-(2-Methoxybenzylidene)-2,4-Thiazolidinedione (**12e**)

Light-yellow solid, yield: 58%; mp: 189.6–191.5 °C. ^1^H NMR (600 MHz, chloroform-*d*) *δ* 8.28 (s, 1H, CH_2_=CH_2_), 7.45 (d, *J* = 7.7 Hz, 1H, Ar-H), 7.41 (t, *J* = 7.9 Hz, 1H, Ar-H), 7.04 (t, *J* = 7.5 Hz, 1H, Ar-H), 6.99 (s, 1H, NH), 6.94 (d, *J* = 8.3 Hz, 1H, Ar-H), 5.26 (d, *J* = 7.0 Hz, 1H, CH_2_=CH_2_), 5.13 (d, *J* = 6.4 Hz, 1H, CH_2_=CH_2_), 4.38 (s, 2H, CH_2_), 4.33–4.28 (m, 1H, CH), 3.90 (s, 3H, OCH_3_), 3.44–3.27 (m, 4H, CH_2_×2, 1H, NH), 1.76–1.71 (m, 3H), 1.70–1.65 (m, 3H), 1.64 (s, 3H, CH_3_), 1.58 (dd, *J* = 14.3, 8.2 Hz, 3H), 1.51–1.44 (m, 3H), 1.40–1.30 (m, 5H), 1.23–1.19 (m, 3H), 1.02 (d, *J* = 5.5 Hz, 3H), 1.00 (d, *J* = 6.1 Hz, 3H), 0.93 (d, *J* = 6.3 Hz, 3H, CH_3_), 0.90–0.83 (m, 9H, CH_3_×3), 0.78 (s, 3H, CH_3_), 0.73 (s, 3H, CH_3_). ^13^C NMR (150 MHz, chloroform-*d*) *δ* 168.43 (C34), 166.14 (C36), 165.91 (C38), 158.64 (C43), 158.45 (C31), 139.97 (C22), 132.56 (C39), 130.53 (C45), 129.57 (C41), 122.48 (C40), 121.35 (C37), 121.00 (C42), 119.03 (C44), 111.28 (C29), 82.27 (C3), 55.64 (C5), 55.54 (C46), 50.39 (C9), 48.81 (C18), 43.95 (C17), 42.44 (C14), 42.29 (C35), 41.83 (C8), 40.37 (C21), 39.30 (C20), 38.49 (C13), 38.12 (C1), 37.06 (C4), 36.81 (C10), 36.43 (C19), 34.50 (C16), 34.24 (C7), 28.12 (C15), 27.74 (C12), 27.14 (C32), 24.13 (C2), 22.69 (C23), 21.76 (C24), 21.71 (C11), 18.27 (C30), 17.83 (C28), 16.66 (C6), 16.42 (C25), 16.14 (C26), 14.92 (C27). HRMS (ESI) *m*/*z*: calcd for C_46_H_65_N_3_O_6_NaS [M+Na]^+^ 810.4492, found 810.4501.

##### Lupeol-3-[(1-Ethylenediaminyl)-4]-5-(2,3,4-Trimethoxybenzylidene)-2,4-Thiazolidinedione (**12f**)

Yellow solid, yield: 51%; mp: 195.6–197.4 °C. ^1^H NMR (600 MHz, chloroform-*d*) *δ* 8.17 (s, 1H, CH_2_=CH_2_), 7.20 (d, *J* = 8.7 Hz, 1H, Ar-H), 6.84 (s, 1H, NH), 6.77 (d, *J* = 8.7 Hz, 1H, Ar-H), 5.26 (d, *J* = 6.8 Hz, 1H, CH_2_=CH_2_), 5.10 (s, 1H, CH_2_=CH_2_), 4.38 (s, 2H, CH_2_), 4.34 (d, *J* = 7.3 Hz, 1H, CH), 3.96–3.91 (m, 6H, OCH_3_), 3.88 (s, 3H, OCH_3_), 3.58–3.14 (m, 4H, CH_2_×2, 1H, NH), 1.77 (dd, *J* = 13.5, 4.8 Hz, 1H), 1.73 (d, *J* = 2.7 Hz, 1H), 1.69 (d, *J* = 13.1 Hz, 2H), 1.63 (s, 3H, CH_3_), 1.60 (s, 1H), 1.56 (d, *J* = 6.8 Hz, 1H), 1.50 (s, 2H), 1.43–1.34 (m, 4H), 1.30 (td, *J* = 13.4, 4.0 Hz, 3H), 1.25 (s, 3H), 1.21 (d, *J* = 15.7 Hz, 2H), 1.06 (d, *J* = 5.6 Hz, 1H), 1.03 (s, 3H), 1.00 (dd, *J* = 12.1, 6.5 Hz, 5H), 0.94 (s, 3H, CH_3_), 0.87 (d, *J* = 16.3 Hz, 6H, CH_3_×2), 0.79 (s, 3H, CH_3_), 0.73 (s, 3H, CH_3_). ^13^C NMR (150 MHz, chloroform-*d*) *δ* 168.40 (C34), 166.17 (C36), 165.91 (C38), 158.34 (C43), 156.46 (C31), 154.05 (C22), 142.48 (C39), 139.98 (C45), 130.08 (C41), 124.79 (C40), 120.47 (C37), 119.48 (C42), 119.02 (C44), 107.68 (C29), 82.22 (C3), 62.03 (C46), 61.08 (C47), 56.28 (C48), 55.57 (C5), 50.42 (C9), 48.81 (C18), 43.92 (C19), 42.44 (C17), 42.28 (C14), 41.19 (C35), 40.37 (C8), 39.31 (C32), 38.56 (C21), 38.16 (C33), 37.09 (C13), 36.80 (C4), 36.43 (C1), 34.50 (C10), 34.25 (C16), 32.48 (C7), 29.82 (C20), 28.12 (C15), 27.73 (C12), 27.15 (C2), 24.17 (C23), 22.66 (C11), 21.76 (C24), 18.28 (C30), 17.83 (C28), 16.67 (C6), 16.45 (C25), 16.14 (C26), 14.85 (C27). HRMS (ESI) *m*/*z*: calcd for C_48_H_69_N_3_O_8_NaS [M+Na]^+^ 870.4703, found 870.4707.

##### Lupeol-3-[(1-Ethylenediaminyl)-4]-5-(2-Furanylmethylene)-2,4-Thiazolidinedione (**12g**)

Light yellow solid, yield: 42%; mp: 187.8–189.2 °C. ^1^H NMR (600 MHz, chloroform-*d*) *δ* 8.07 (d, *J* = 8.8 Hz, 1H, CH_2_=CH_2_), 7.67 (s, 1H, NH), 7.26–7.13 (m, 1H, CH_2_=CH_2_), 6.88 (d, *J* = 8.7 Hz, 1H, CH_2_=CH_2_), 6.80 (d, *J* = 3.7 Hz, 1H, CH_2_=CH_2_), 6.58 (d, *J* = 3.5 Hz, 1H, CH_2_=CH_2_), 5.27–5.24 (m, 1H, CH_2_=CH_2_), 4.39 (s, 2H, CH_2_), 4.30 (tt, *J* = 10.9, 5.2 Hz, 1H, CH), 3.49–3.18 (m, 4H, CH_2_×2, 1H, NH), 2.09–1.91 (m, 1H), 1.69 (d, *J* = 12.5 Hz, 3H), 1.63 (s, 3H, CH_3_), 1.61–1.53 (m, 3H), 1.52–1.47 (m, 2H), 1.42 (dd, *J* = 9.3, 5.2 Hz, 2H), 1.37 (d, *J* = 9.6 Hz, 2H), 1.31 (d, *J* = 8.5 Hz, 1H), 1.25 (s, 4H), 1.16 (s, 1H), 1.04 (d, *J* = 5.0 Hz, 3H), 0.99 (dd, *J* = 10.6, 3.9 Hz, 4H), 0.95 (d, *J* = 4.5 Hz, 3H, CH_3_), 0.89–0.83 (m, 9H, CH_3_×3), 0.78 (s, 3H, CH_3_), 0.72 (d, *J* = 18.2 Hz, 3H, CH_3_). ^13^C NMR (150 MHz, chloroform-*d*) *δ* 168.82 (C34), 166.43 (C36), 165.82 (C38), 163.09 (C40), 149.75 (C31), 146.79 (C22), 140.90 (C39), 126.19 (C43), 120.34 (C37), 118.37 (C41), 115.79 (C42), 113.41 (C29), 82.44 (C3), 55.53 (C5), 50.70 (C9), 50.40 (C18), 48.80 (C19), 44.76 (C17), 43.82 (C14), 42.44 (C35), 42.27 (C8), 41.78 (C32), 41.17 (C21), 40.32 (C33), 39.29 (C13), 38.48 (C4), 38.11 (C1), 37.25 (C10), 37.07 (C15), 36.78 (C16), 36.41 (C7), 34.48 (C20), 32.47 (C12), 29.81 (C2), 28.11 (C23), 24.14 (C11), 22.67 (C6), 21.75 (C24), 17.81 (C30), 16.40 (C28), 16.13 (C25), 14.89 (C26), 12.07 (C27). HRMS (ESI) *m*/*z*: calcd for C_43_H_61_N_3_O_6_NaS [M+Na]^+^ 770.4179, found 770.4187.

##### Lupeol-3-[(1-Ethylenediaminyl)-4]-5-(2-Thienylmethylene)-2,4-Thiazolidinedione (**12h**)

Light yellow solid, yield: 47%; mp: 193.5–195.3 °C. ^1^H NMR (600 MHz, chloroform-*d*) *δ* 8.09 (s, 1H, CH_2_=CH_2_), 7.66 (d, *J* = 5.0 Hz, 1H, CH_2_=CH_2_), 7.42 (d, *J* = 3.9 Hz, 1H, CH_2_=CH_2_), 7.19 (q, *J* = 4.5 Hz, 1H, CH_2_=CH_2_), 7.15–7.07 (m, 1H, NH), 5.27 (d, *J* = 6.9 Hz, 1H, CH_2_=CH_2_), 5.13 (t, *J* = 6.3 Hz, 1H, CH_2_=CH_2_), 4.38 (s, 2H, CH_2_), 4.29 (dd, *J* = 11.7, 4.5 Hz, 1H, CH), 3.45–3.26 (m, 4H, CH_2_×2, 1H, NH), 1.78–1.71 (m, 3H, CH_3_), 1.68 (d, *J* = 12.1 Hz, 2H), 1.64 (s, 3H, CH_3_), 1.61 (s, 1H), 1.56 (dd, *J* = 16.4, 7.2 Hz, 2H), 1.49 (q, *J* = 7.9 Hz, 3H), 1.41 (d, *J* = 4.7 Hz, 1H), 1.36 (d, *J* = 9.6 Hz, 2H), 1.33–1.30 (m, 2H), 1.26 (d, *J* = 9.8 Hz, 5H), 1.04 (s, 2H), 1.01–0.99 (m, 3H), 0.96 (s, 3H, CH_3_), 0.86 (d, *J* = 12.9 Hz, 9H, CH_3_×3), 0.78 (s, 3H, CH_3_), 0.74 (s, 3H, CH_3_). ^13^C NMR (150 MHz, chloroform-*d*) *δ* 167.32 (C36), 165.78 (C34), 158.55 (C38), 139.97 (C40), 137.71 (C31), 133.75 (C22), 132.26 (C39), 129.90 (C43), 128.80 (C37), 127.08 (C41), 119.33 (C42), 119.03 (C29), 82.28 (C3), 55.53 (C5), 50.43 (C9), 48.82 (C18), 44.15 (C19), 42.46 (C17), 42.28 (C14), 41.95 (C35), 41.18 (C8), 40.34 (C32), 39.31 (C21), 38.52 (C33), 38.11 (C13), 37.08 (C4), 36.80 (C1), 36.43 (C10), 34.50 (C15), 34.25 (C16), 29.82 (C7), 28.11 (C20), 27.77 (C12), 27.15 (C2), 24.15 (C23), 22.70 (C11), 21.76 (C6), 18.26 (C24), 17.83 (C30), 16.67 (C28), 16.43 (C25), 16.09 (C26), 14.97 (C27). HRMS (ESI) *m*/*z*: calcd for C_43_H_61_N_3_O_5_NaS_2_ [M+Na]^+^ 786.3950, found 786.3953.

##### Lupeol-3-[(1-Ethylenediaminyl)-4]-5-(2-Methylpropylidene)-2,4-Thiazolidinedione (**12i**)

White solid, yield: 43%; mp: 191.7–193.6 °C. ^1^H NMR (600 MHz, chloroform-*d*) *δ* 7.03–7.00 (m, 1H, NH), 6.96 (d, *J* = 9.7 Hz, 1H, CH_2_=CH_2_), 5.31–5.25 (m, 1H, CH_2_=CH_2_), 5.22 (t, *J* = 6.2 Hz, 1H, CH_2_=CH_2_), 4.34 (s, 1H, CH), 4.32 (s, 2H, CH_2_), 3.43–3.26 (m, 4H, CH_2_×2, 1H, NH), 1.77–1.73 (m, 2H), 1.71–1.66 (m, 3H), 1.63 (s, 3H, CH_3_), 1.55 (dd, *J* = 16.0, 7.2 Hz, 3H, CH_3_), 1.40–1.37 (m, 3H, CH_3_), 1.31 (s, 1H), 1.30–1.30 (m, 2H), 1.29 (s, 2H), 1.27 (s, 6H), 1.05 (s, 3H), 0.99 (s, 2H), 0.95 (s, 2H), 0.89 (s, 3H, CH_3_), 0.88 (s, 6H, CH_3_×2), 0.87 (s, 3H, CH_3_), 0.80 (s, 3H, CH_3_), 0.74 (s, 3H, CH_3_). ^13^C NMR (150 MHz, chloroform-*d*) *δ* 167.76 (C34), 165.89 (C36), 165.05 (C38), 158.30 (C31), 145.30 (C22), 139.95 (C39), 123.00 (C37), 119.01 (C29), 82.13 (C3), 55.59 (C5), 50.47 (C9), 48.80 (C18), 43.68 (C19), 42.44 (C17), 42.28 (C1), 41.49 (C10), 41.19 (C8), 40.38 (C32), 39.31 (C21), 38.61 (C33), 38.16 (C13), 37.10 (C4), 36.80 (C1), 36.43 (C10), 34.49 (C15), 34.27 (C16), 32.06 (C40), 29.82 (C7), 28.10 (C20), 27.72 (C12), 27.14 (C2), 24.18 (C23), 22.65 (C11), 21.73 (C6), 21.39 (C41, C42), 18.28 (C24), 17.81 (C30), 16.65 (C28), 16.45 (C25), 16.14 (C26), 14.83 (C27). HRMS (ESI) *m*/*z*: calcd for C_42_H_65_N_3_O_5_NaS [M+Na]^+^ 746.4543, found 746.4545.

### 3.2. Cell Culture

The human cancer cell lines HepG2, A549, and MCF-7 were purchased from the cell bank of the Chinese Academy of Sciences (Shanghai, China). The human hepatic cell line LO2 was obtained from iCell Bioscience Inc. (Shanghai, China). These cell lines were cultured in Dulbecco’s Modified Eagle Medium containing 10% (*v*/*v*) fetal bovine serum (FBS, Wisent) and 1% (*v*/*v*) Penicillin–Streptomycin Solution mixture (Beyotime, Shanghai, China) at 37 °C under 5% CO_2_.

### 3.3. In Vitro Antiproliferative Assay

An MTT assay evaluated the cytotoxicity of lupeol derivatives toward the A549, MCF-7, HepG2, and LO2 cell lines. The cells were inoculated into 96-well plates (3 × 10^3^ cells/well) and cultured for 24 h at 37 °C in 5% CO_2_. After that, a gradient concentration of the test drug was added to continue the incubation for 48 h. Subsequently, the MTT solution continued to incubate with the cells for 4 h. Afterward, the upper waste layer was discarded, and DMSO was added (Kermel, Tianjin, China). After the formazan completely dissolved, the absorbance of each well at 490 nm was measured with a microplate reader. The mean values were derived from three independent experiments.

### 3.4. Apoptosis Analysis Using Flow Cytometry

HepG2 cells were inoculated into 6-well plates for 24 h, attached via cell adhesion, and then treated with **12i** (0, 2, 4, and 8 μM) for 48 h. The cells were harvested and treated with Annexin V-FITC/PI staining solution, and the data were gathered using a flow cytometer (FACS Calibur, BD, Milpitas, CA, USA).

### 3.5. Analysis of Apoptosis Using Confocal Microscopy

HepG2 cells were seeded in 24-well laser confocal plates for 24 h. Then, gradient concentrations (0, 2, 4, and 8 µM) of **12i** were added and co-incubated for 48 h. The supernatant was then discarded, the cells were washed using PBS, and 5 µL each of AO and EB dyes was added at 25 °C for 20 min. Then, the dyes were removed, and PBS was added. Finally, the samples were collected and imaged using confocal microscopy (LSM710, Zeiss, Oberkochen, Germany).

### 3.6. Mitochondrial Membrane Potential Analysis Using Flow Cytometry

HepG2 cells were inoculated into 6-well plates for 24 h, attached via cell adhesion, and then treated with **12i** (0, 2, 4, and 8 μM) for 48 h. The cells were cleaned with PBS and treated with JC-1 solution at 25 °C for 20 min, and the data were gathered using a flow cytometer.

### 3.7. Mitochondrial Membrane Potential Analysis Using Confocal Microscopy

HepG2 cells were inoculated into 6-well plates for 24 h, attached via cell adhesion, and then treated with **12i** (0, 2, 4, and 8 μM) for 48 h. The cells were cleaned with PBS and treated with JC-1 solution at 25 °C for 20 min, and the samples were collected and imaged using confocal microscopy.

### 3.8. ROS Content Detection Using Flow Cytometry

HepG2 cells were inoculated into 6-well plates for 24 h, attached via cell adhesion, and treated with **12i** (0, 2, 4, and 8 μM) for 48 h. After the cells were washed with PBS, the supernatant was removed and incubated with 500 μL of 10 μm/L DCFH-DA solution at 25 °C for 20 min, and the data were gathered using a flow cytometer.

### 3.9. Measurement of ROS Using Confocal Microscopy

HepG2 cells were inoculated into 6-well plates for 24 h, attached via cell adhesion, and treated with **12i** (0, 2, 4, and 8 μM) for 48 h. After the cells were washed with PBS, the supernatant was removed and incubated with 500 μL of 10 μm/L DCFH-DA solution at 25 °C for 20 min, and the samples were collected and imaged using confocal microscopy.

### 3.10. Western Blot Analysis

HepG2 cells were seeded in 6-well plates at a density of 1 × 10^6^ overnight and treated with **12i** (2, 4, and 8 μM) for 48 h. They were then washed twice with cooled PBS to remove the medium and lysed using a lysis buffer containing RIPA (Beyotime, Shanghai, China). The protein levels were quantified using a bicinchoninic acid protein assay kit (Beyotime, Shanghai, China). The proteins were separated via SDS-PAGE electrophoresis and transferred to PVDF membranes (Beyotime, Shanghai, China). After blocking with 5% skim milk for 2 h, the membranes were incubated with the primary antibody overnight at 4 °C, followed by 2 h with the specific secondary antibody. The antibodies were sourced commercially, including Bax (5023T, Cell Signaling Technology, Danvers, MA, USA), Bcl2 (3498T, Cell Signaling Technology, Danvers, MA, USA), Caspase-7 (12827T, Cell Signaling Technology, Danvers, MA, USA), Caspase-9 (9508T, Cell Signaling Technology, Danvers, MA, USA), Cleaved-Caspase-7 (8438T, Cell Signaling Technology, Danvers, MA, USA), Cleaved-Caspase-9 (7237T, Cell Signaling Technology, Danvers, MA, USA), GAPDH (2118T, Cell Signaling Technology, Danvers, MA, USA), PI3K (4249T, Cell Signaling Technology, Danvers, MA, USA), p-PI3K (4228T, Cell Signaling Technology, Danvers, MA, USA), AKT (4691T, Cell Signaling Technology, Danvers, MA, USA), p-AKT (4060T, Cell Signaling Technology, Danvers, MA, USA), mTOR (2972T, Cell Signaling Technology, Danvers, MA, USA). Immunoreactive bands were visualized using BeyoECL Plus (Beyotime, Shanghai, China) and scanned in a ChemiDoc MP Imaging System (Bio-Rad, Shanghai, China).

## 4. Conclusions

In conclusion, we successfully designed and synthesized a series of novel lupeol-3-thiazolidinedione derivatives by altering the linker between lupeol and thiazolidinedione. An MTT assay was conducted to evaluate the in vitro antiproliferative activity of all compounds against three cancer cell lines and a normal cell line. We successfully selected compound **12i**, which exhibited strong antitumor effects in HepG2 cells, with an IC_50_ of 4.40 μM that was 9.9-fold higher than that of the parent lupeol. According to the results of the in vitro antitumor activity test, the IC_50_ value of compound **12i** is marginally higher than that of the marketed drug cisplatin. In addition, compound **12i** was weakly cytotoxic to human hepatic cell LO2 and, to some extent, selective for cancer cells. Mechanistic studies demonstrated that compound **12i** promotes ROS production, decreases mitochondrial membrane potential, and induces HepG2 cell apoptosis through the mitochondria pathway. Thus, compound **12i** provides a lead structure for the development of novel anticancer drugs.

## Figures and Tables

**Table 1:** IC_50_ values of lupeol derivatives **6a**–**i**, **9a**–**i**, and **12a**–**i** tested against a panel of cell lines for 48 h. SI (selectivity index) = IC_50_ values on L02 cells/HepG2 cells.

Compounds	IC_50_ (μM) *				SI
	A549	MCF-7	HepG2	LO2	
**6a**	17.30 ± 1.58	23.99 ± 1.87	22.72 ± 1.59	92.56 ± 1.03	4.07
**6b**	14.16 ± 1.72	13.78 ± 0.75	16.58 ± 1.38	70.48 ± 1.51	4.25
**6c**	10.37 ± 0.82	22.12 ± 1.42	18.20 ± 1.27	76.44 ± 1.22	4.2
**6d**	14.19 ± 1.43	15.41 ± 1.22	10.86 ± 0.78	39.25 ± 1.07	3.61
**6e**	9.43 ± 0.74	12.52 ± 0.74	15.48 ± 0.86	70.82 ± 0.93	4.57
**6f**	10.34 ± 0.86	16.10 ± 0.89	19.22 ± 1.57	85.34 ± 1.12	4.44
**6g**	14.94 ± 1.21	12.79 ± 0.22	15.34 ± 1.32	60.57 ± 1.84	3.95
**6h**	14.03 ± 1.39	11.34 ± 0.85	12.82 ± 0.97	58.36 ± 0.78	4.55
**6i**	11.35 ± 0.91	8.70 ± 0.12	9.48 ± 0.71	45.53 ± 1.33	4.80
**9a**	18.71 ± 1.25	19.07 ± 1.78	20.46 ± 1.62	79.14 ± 1.25	3.87
**9b**	15.47 ± 0.64	14.04 ± 0.87	18.11 ± 1.73	80.49 ± 1.79	4.44
**9c**	12.74 ± 0.68	16.27 ± 0.76	15.71 ± 1.52	65.11 ± 0.93	4.14
**9d**	11.71 ± 0.75	18.12 ± 1.93	9.52 ± 0.61	42.17 ± 1.28	4.43
**9e**	10.37 ± 0.38	18.60 ± 1.27	16.52 ± 1.26	63.31 ± 1.14	3.83
**9f**	9.24 ± 0.48	9.19 ± 0.46	10.81 ± 0.45	56.08 ± 0.77	5.19
**9g**	9.91 ± 0.42	12.04 ± 0.75	14.98 ± 0.74	62.77 ± 0.62	4.19
**9h**	16.45 ± 1.69	13.83 ± 0.58	15.33 ± 0.98	68.93 ± 1.19	4.50
**9i**	13.78 ± 0.84	20.13 ± 1.59	6.91 ± 0.31	35.40 ± 0.72	5.12
**12a**	10.53 ± 0.56	16.83 ± 1.29	16.51 ± 0.54	71.03 ± 0.57	4.30
**12b**	17.05 ± 1.28	19.11 ± 1.63	17.67 ± 0.79	76.87 ± 0.69	4.35
**12c**	13.39 ± 0.79	14.29 ± 0.78	10.74 ± 0.12	40.68 ± 0.31	3.79
**12d**	16.37 ± 0.43	9.12 ± 0.24	15.75 ± 0.68	59.91 ± 1.03	3.80
**12e**	13.77 ± 0.76	14.38 ± 0.72	12.93 ± 0.52	57.67 ± 1.14	4.46
**12f**	9.68 ± 0.52	10.02 ± 0.22	9.43 ± 0.78	35.53 ± 0.34	3.77
**12g**	15.70 ± 1.42	16.44 ± 0.87	14.29 ± 0.56	63.22 ± 1.31	4.42
**12h**	13.40 ± 1.08	17.32 ± 1.28	18.62 ± 1.49	77.51 ± 1.27	4.14
**12i**	10.06 ± 0.62	8.74 ± 0.34	4.40 ± 0.02	29.23 ± 0.53	6.69
Lupeol	35.86 ± 1.18	62.03 ± 1.79	43.62 ± 1.37	-	-
Cisplatin	4.96 ± 0.48	5.79 ± 0.49	4.65 ± 0.54	-	-

* The data are expressed as means ± standard deviation (SD) from three independent experiments.

## Data Availability

The original contributions presented in the study are included in the article and Supplementary Material, further inquiries can be directed to the corresponding authors.

## Supplementary Materials

The following supporting information can be downloaded at: https://www.mdpi.com/article/10.3390/molecules29204957/s1. Supplementary File: HRMS, ^1^H NMR, and ^13^C NMR spectral data of all derivatives.

## References

[1] Sung H., Ferlay J., Siegel R.L., Laversanne M., Soerjomataram I., Jemal A., Bray F. (2021). Global Cancer Statistics 2020: GLOBOCAN Estimates of Incidence and Mortality Worldwide for 36 Cancers in 185 Countries. CA. Cancer. J. Clin..

[2] Siegel R.L., Miller K.D., Wagle N.S., Jemal A. (2023). Cancer statistics, 2023. CA. Cancer. J. Clin..

[3] Fujita S., Kotake K. (2014). Chemotherapy. Nihon. Rinsho..

[4] Liu S., Khan A.R., Yang X., Dong B., Ji J., Zhai G. (2021). The reversal of chemotherapy-induced multidrug resistance by nanomedicine for cancer therapy. J. Control. Release..

[5] Mir R.H., Mir P.A., Uppal J., Chawla A., Patel M., Bardakci F., Adnan M., Mohi-Ud-Din R. (2023). Evolution of Natural Product Scaffolds as Potential Proteasome Inhibitors in Developing Cancer Therapeutics. Metabolites.

[6] Naeem A., Hu P., Yang M., Zhang J., Liu Y., Zhu W., Zheng Q. (2022). Natural Products as Anticancer Agents: Current Status and Future Perspectives. Molecules.

[7] Wang X., Zhuang Y., Wang Y., Jiang M., Yao L. (2023). The recent developments of camptothecin and its derivatives as potential anti-tumor agents. Eur. J. Med. Chem..

[8] Song Z., Lu Q., Tao A., Wu T. (2021). Synthesis and Anti-cancer Activity of Paclitaxel-Coumarin Conjugate. Curr. Org. Synth..

[9] Xiao J., Gao M., Sun Z., Diao Q., Wang P., Gao F. (2020). Recent advances of podophyllotoxin/epipodophyllotoxin hybrids in anticancer activity, mode of action, and structure-activity relationship: An update (2010–2020). Eur. J. Med. Chem..

[10] Imam S., Azhar I., Hasan M.M., Ali M.S., Ahmed S.W. (2007). Two triterpenes lupanone and lupeol isolated and identified from Tamarindus indica linn. Pak. J. Pharm. Sci..

[11] You Y.J., Nam N.H., Kim Y., Bae K.H., Ahn B.Z. (2003). Antiangiogenic activity of lupeol from Bombax ceiba. Phytother. Res..

[12] Fernández M.A., de las Heras B., García M.D., Sáenz M.T., Villar A. (2001). New insights into the mechanism of action of the anti-inflammatory triterpene lupeol. J. Pharm. Pharmacol..

[13] Molnár J., Gyémánt N., Tanaka M., Hohmann J., Bergmann-Leitner E., Molnár P., Deli J., Didiziapetris R., Ferreira M.J. (2006). Inhibition of multidrug resistance of cancer cells by natural diterpenes, triterpenes and carotenoids. Curr. Pharm. Des..

[14] Zhang L., Zhang Y., Zhang L., Yang X., Lv Z. (2009). Lupeol, a dietary triterpene, inhibited growth, and induced apoptosis through down-regulation of DR3 in SMMC7721 cells. Cancer. Invest..

[15] Liu K., Zhang X., Xie L., Deng M., Chen H., Song J., Long J., Li X., Luo J. (2021). Lupeol and its derivatives as anticancer and anti-inflammatory agents: Molecular mechanisms and therapeutic efficacy. Pharmacol. Res..

[16] Najid A., Simon A., Cook J., Chable-Rabinovitch H., Delage C., Chulia A.J., Rigaud M. (1992). Characterization of ursolic acid as a lipoxygenase and cyclooxygenase inhibitor using macrophages, platelets and differentiated HL60 leukemic cells. FEBS. Lett..

[17] Nagumo A., Takanashi K., Hojo H., Suzuki Y. (1991). Cytotoxicity of bacteriohopane-32-ol against mouse leukemia L1210 and P388 cells in vitro. Toxicol. Lett..

[18] Min T.R., Park H.J., Ha K.T., Chi G.Y., Choi Y.H., Park S.H. (2019). Suppression of EGFR/STAT3 activity by lupeol contributes to the induction of the apoptosis of human non-small cell lung cancer cells. Int. J. Oncol..

[19] Bhattacharyya S., Mitra D., Ray S., Biswas N., Banerjee S., Majumder B., Mustafi S.M., Murmu N. (2019). Reversing effect of Lupeol on vasculogenic mimicry in murine melanoma progression. Microvasc. Res..

[20] Zhang X., Gao Z., Chen K., Zhuo Q., Chen M., Wang J., Lai X., Wang L. (2022). Lupeol inhibits the proliferation and migration of MDA-MB-231 breast cancer cells via a novel crosstalk mechanism between autophagy and the EMT. Food. Funct..

[21] Fatma H., Jameel M., Siddiqui A.J., Kuddus M., Buali N.S., Bahrini I., Siddique H.R. (2024). Chemotherapeutic potential of lupeol against cancer in pre-clinical model: A systematic review and meta-analysis. Phytomedicine.

[22] Nigam N., Prasad S., Shukla Y. (2007). Preventive effects of lupeol on DMBA induced DNA alkylation damage in mouse skin. Food. Chem. Toxicol..

[23] Bu M., Wang L., Luo R., Xu T., Lin Y., Ge P., Liu J. (2024). Lupinol-3β-succinate induce apoptosis of HepG2 cells by regulating the mitochondrial apoptosis pathway. Drugs Clin. Drugs Clin..

[24] Tian S., Zhao Y., Deng S., Hou L., Song J., Wang M., Bu M. (2024). Lupeol-3-carbamate Derivatives: Synthesis and Biological Evaluation as Potential Antitumor Agents. Molecules.

[25] Ortiz A., Sansinenea E. (2011). Synthetic thiazolidinediones: Potential antidiabetic compounds. Curr. Org. Chem..

[26] Kaur Manjal S., Kaur R., Bhatia R., Kumar K., Singh V., Shankar R., Kaur R., Rawal R.K. (2017). Synthetic and medicinal perspective of thiazolidinones: A review. Bioorg. Chem..

[27] Mansure J.J., Nassim R., Kassouf W. (2009). Peroxisome proliferator-activated receptor gamma in bladder cancer: A promising therapeutic target. Cancer. Biol. Ther..

[28] Fu X., Mao Q., Zhang B., Lv J., Ping K., Zhang P., Lin F., Zhao J., Feng Y., Yang J. (2022). Thiazolidinedione-Based Structure Modification of Celastrol Provides Thiazolidinedione-Conjugated Derivatives as Potent Agents against Non-Small-Cell Lung Cancer Cells through a Mitochondria-Mediated Apoptotic Pathway. J. Nat. Prod..

[29] Knight S.D., Adams N.D., Burgess J.L., Chaudhar A.M., Darcy M.G., Donatelli C.A., Luengo J.I., Newlander K.A., Parrish C.A., Ridgers L.H. (2010). Discovery of GSK2126458, a Highly Potent Inhibitor of PI3K and the Mammalian Target of Rapamycin. ACS. Med. Chem. Lett..

[30] Liu K., Rao W., Parikh H., Li Q., Guo T.L., Grant S., Kellogg G.E., Zhang S. (2012). 3,5-Disubstituted-thiazolidine-2,4-dione analogs as anticancer agents: Design, synthesis and biological characterization. Eur. J. Med. Chem..

[31] Sun J., Chen Y.H., Liu H.Y., Hondo E., Zhou Y., Wu Y.F. (2021). Thiazolidinedione: A Privileged Scaffold for the Development of Anticancer Agents. Curr. Top. Med. Chem..

[32] Sethi N.S., Prasad D.N., Singh R.K. (2020). An Insight into the Synthesis and SAR of 2,4-Thiazolidinediones (2,4-TZD) as Multifunctional Scaffold: A Review. Mini. Rev. Med. Chem..

[33] Wu S., Zhang Y., He X., Che X., Wang S., Liu Y., Jiang Y., Liu N., Dong G., Yao J. (2014). From antidiabetic to antifungal: Discovery of highly potent triazole-thiazolidinedione hybrids as novel antifungal agents. Chem. Med. Chem..

[34] Sahoo B.M., Banik B.K., Borah P., Jain A. (2022). Reactive Oxygen Species (ROS): Key Components in Cancer Therapies. Anticancer. Agents. Med. Chem..

[35] Jin X.Y., Chen H., Li D.D., Li A.L., Wang W.Y., Gu W. (2019). Design, synthesis, and anticancer evaluation of novel quinoline derivatives of ursolic acid with hydrazide, oxadiazole, and thiadiazole moieties as potent MEK inhibitors. J. Enzyme. Inhib. Med. Chem..

