# Supplementary material for: Thiazolidinedione-Conjugated Lupeol Derivatives as Potent Anticancer Agents Through a Mitochondria-Mediated Apoptotic Pathway
Source: Molecules. 2024 Oct 20;29(20):4957. doi: 10.3390/molecules29204957 (PMC11510666; doi:10.3390/molecules29204957)

Supporting Information

Thiazolidinedione-Based Structure Modification of Lupeol Provides Thiazolidinedione-Conjugated Derivatives as Potent Agents against Hepatocarcinoma Cells through a Mitochondria-Mediated Apoptotic Pathway

Siqi Deng [1], Yinxu Zhao [1], Xiaoshan Guo [1], Xian Hong [2], Gang Li [2], Yuchun Wang [1], Qingyi Li [1], Ming Bu [1,*], Ming Wang [1,*]

[1] *College of Pharmacy, Qiqihar Medical University, Qiqihar, 161006, Heilongjiang, PR China*

[2] *Research Institute of Medicine & Pharmacy, Qiqihar Medical University, Qiqihar, 161006, Heilongjiang, PR China*

[*] *Corresponding author.*

E-mail address: buming@qmu.edu.cn (M. Bu); wangming_qy@163.com (M. Wang)

^{1}H NMR, ^{13}C NMR and ESI-HRMS spectra of target compound ***6a***

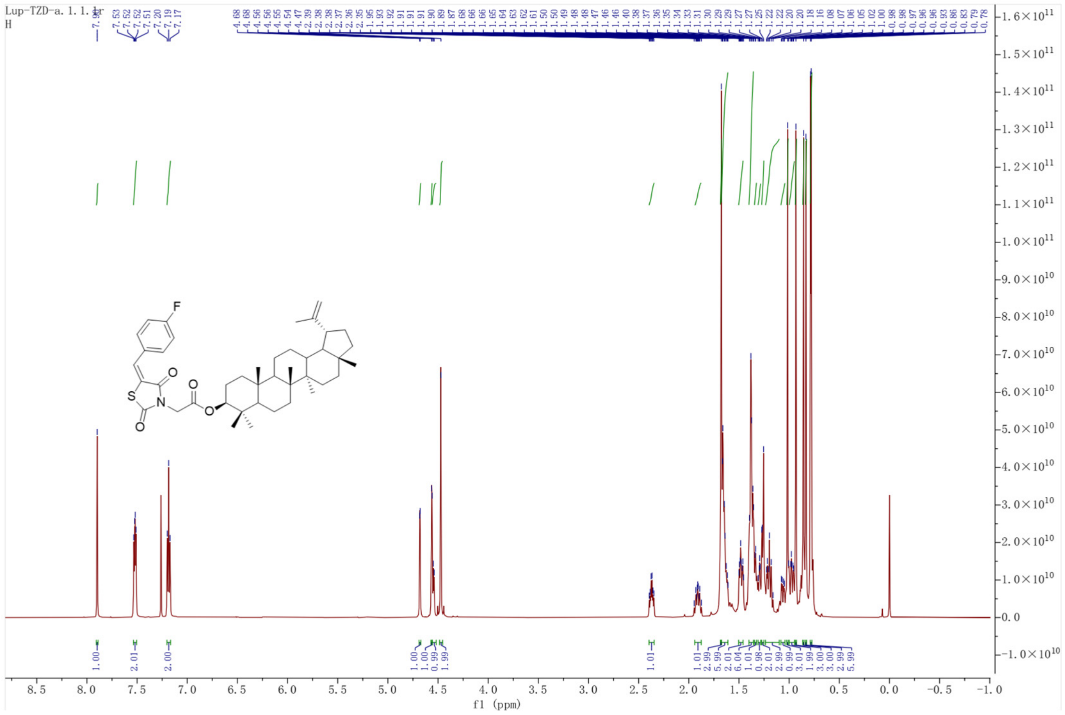

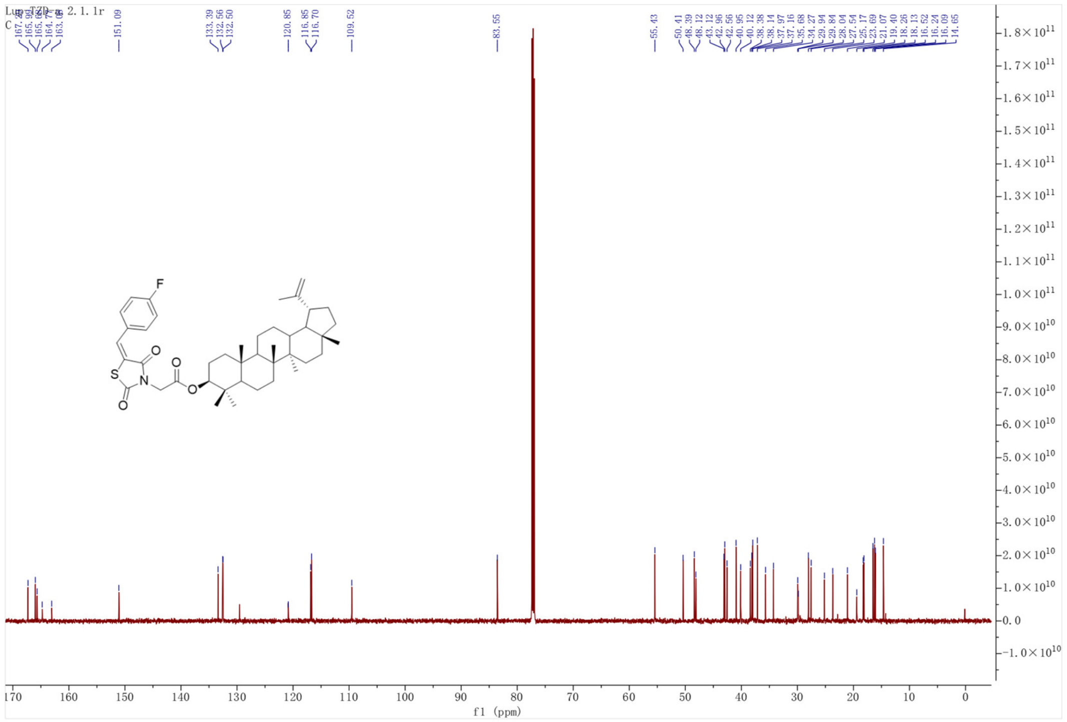

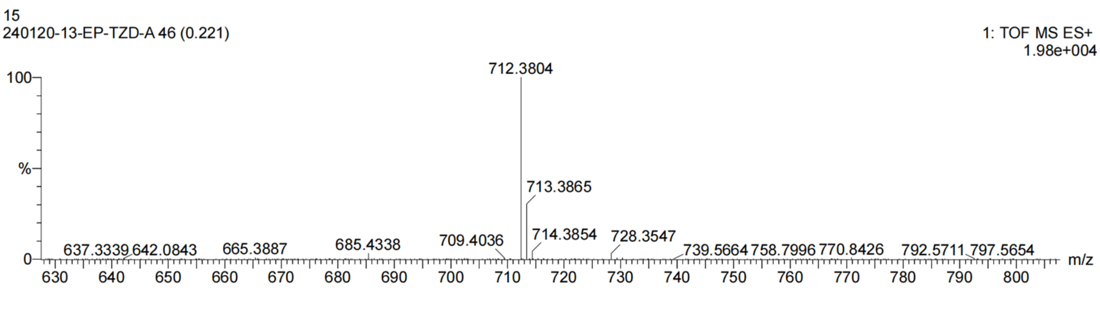

^{1}H NMR, ^{13}C NMR and ESI-HRMS spectra of target compound ***6b***

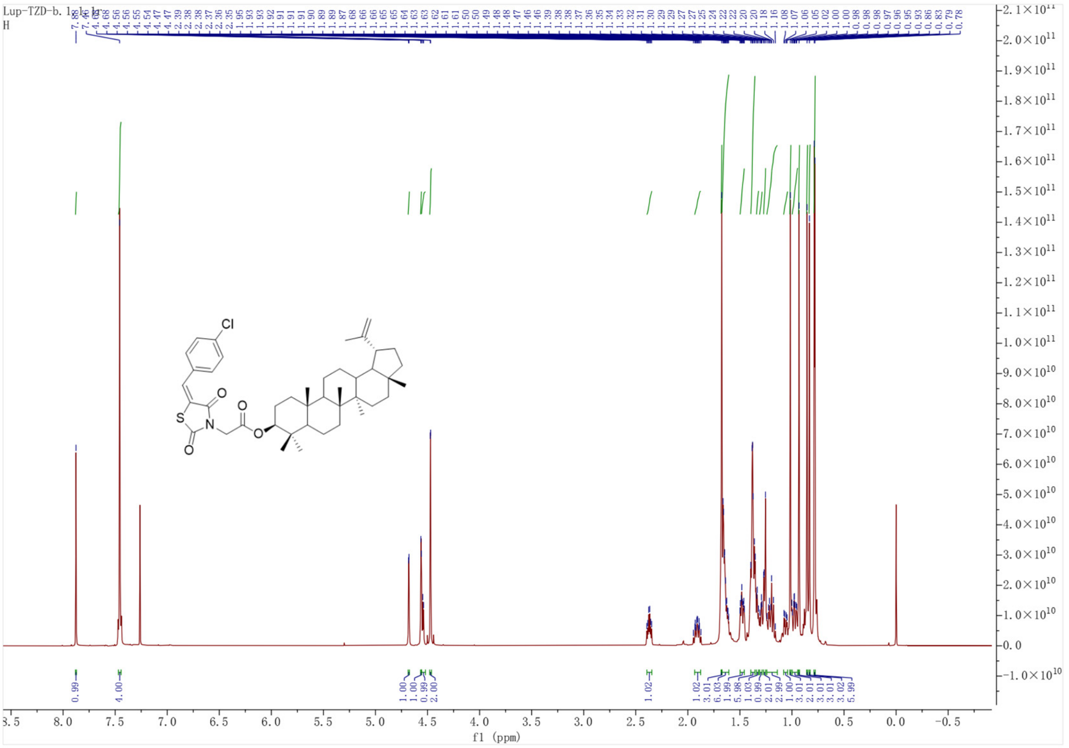

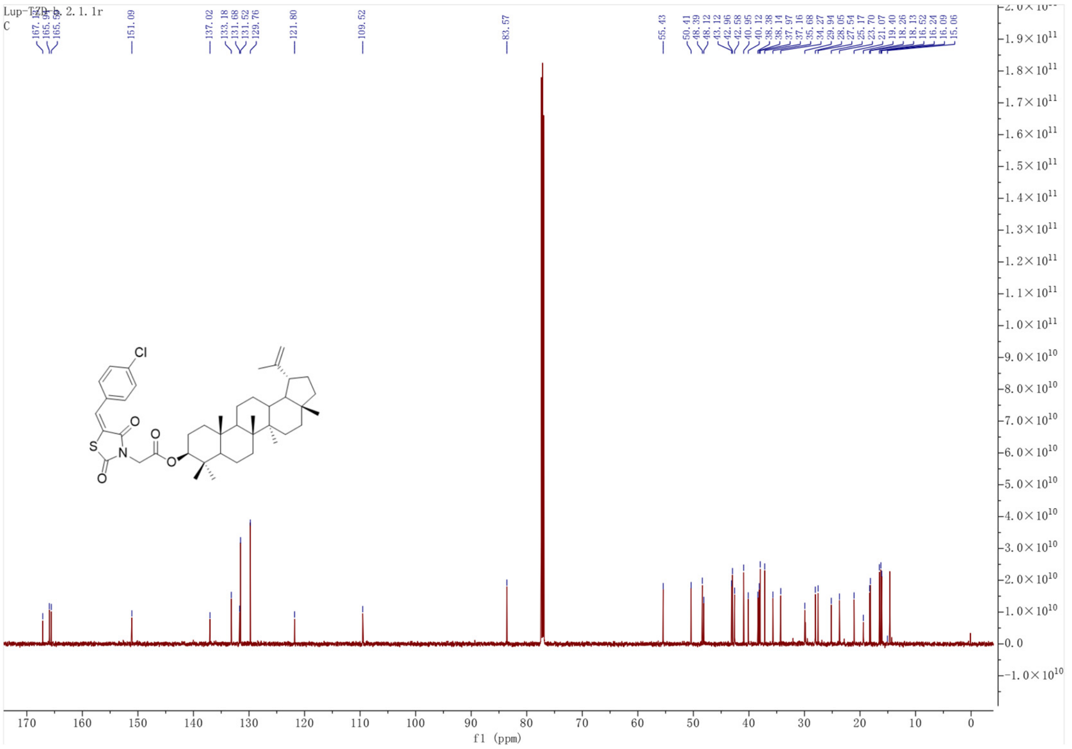

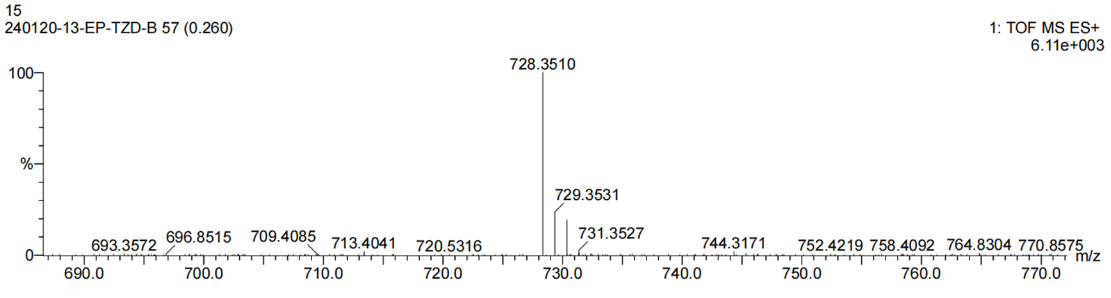

*^{1}H NMR, ^{13}C NMR and ESI-HRMS spectra of target compound **6c***

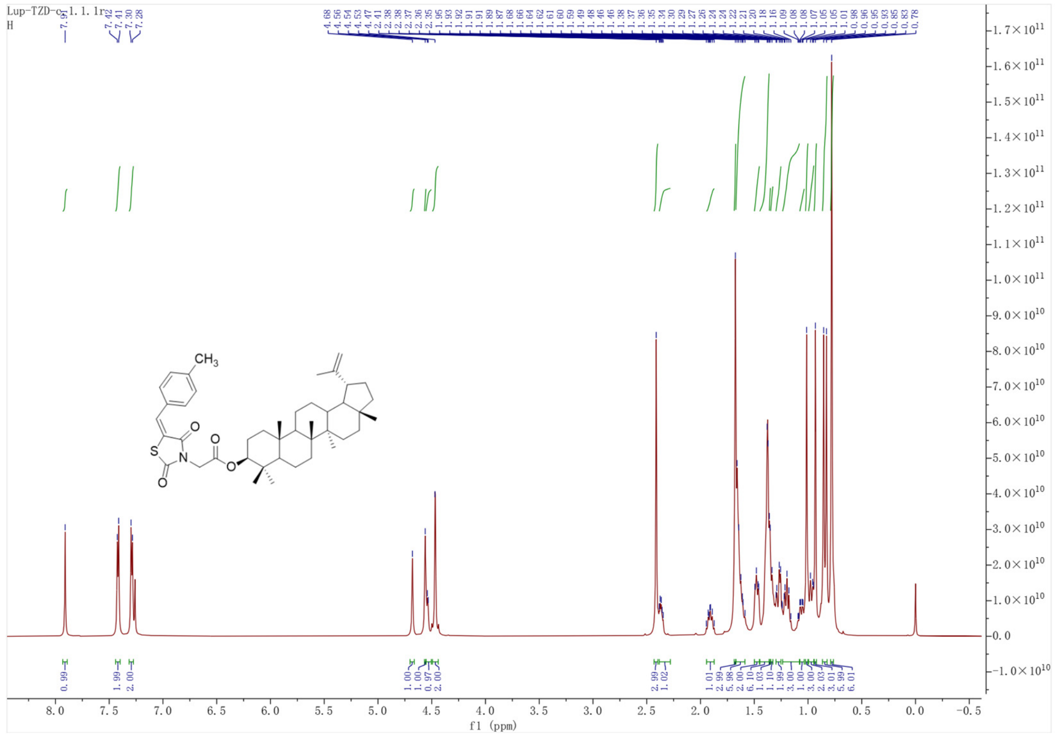

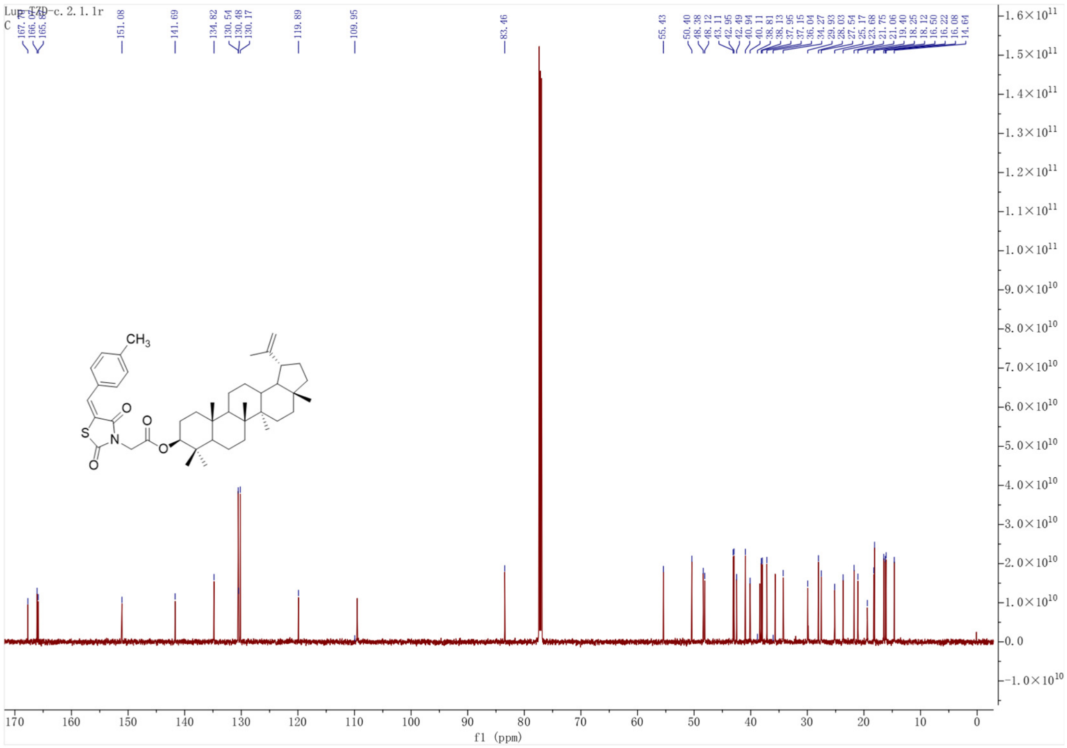

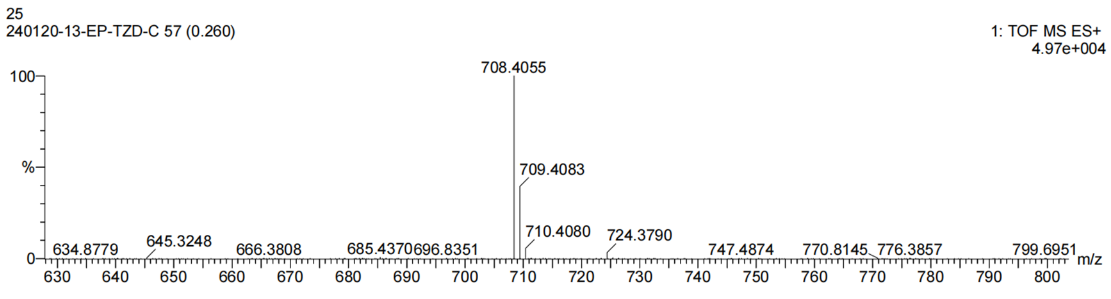

^{1}H NMR, ^{13}C NMR and ESI-HRMS spectra of target compound ***6d***

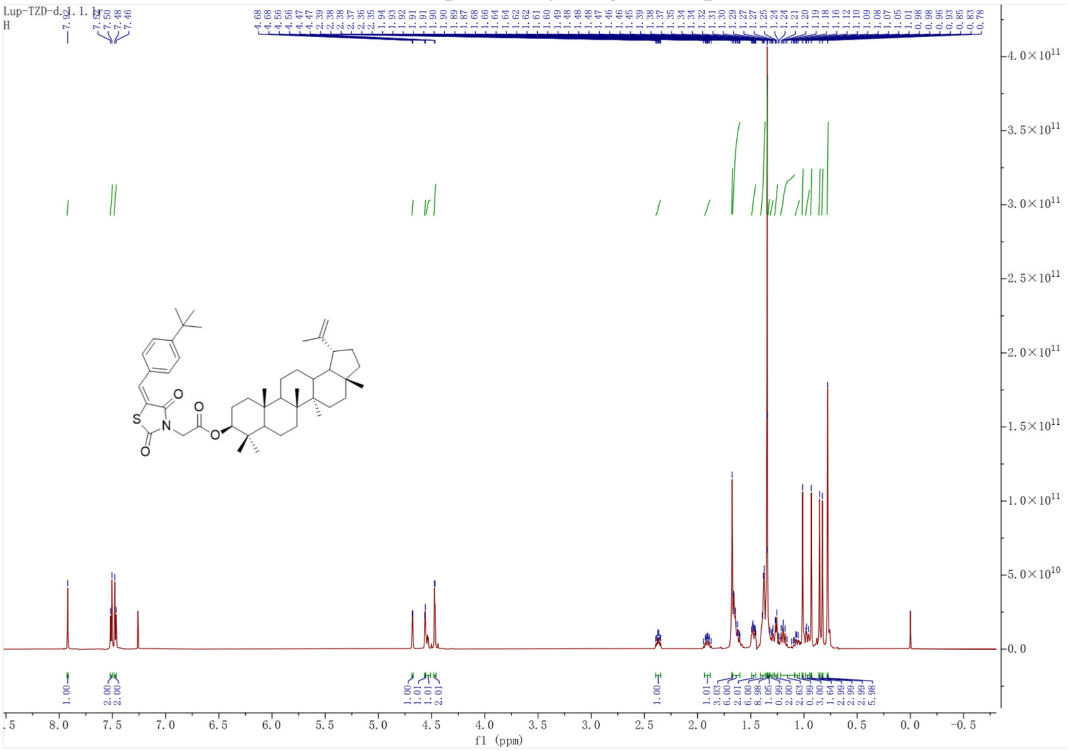

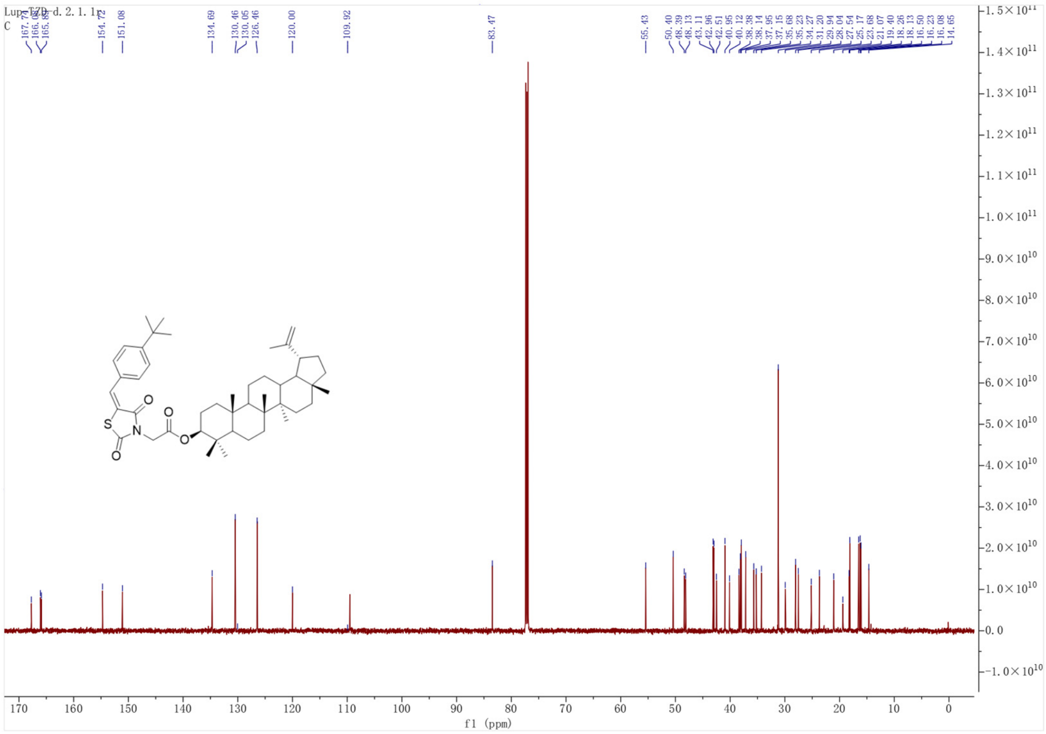

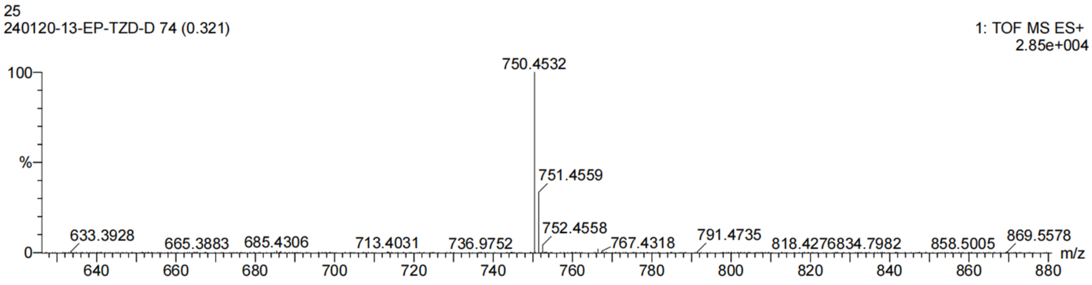

^{1}*H NMR,* ^{13}*C NMR and ESI-HRMS spectra of target compound* ***6e***

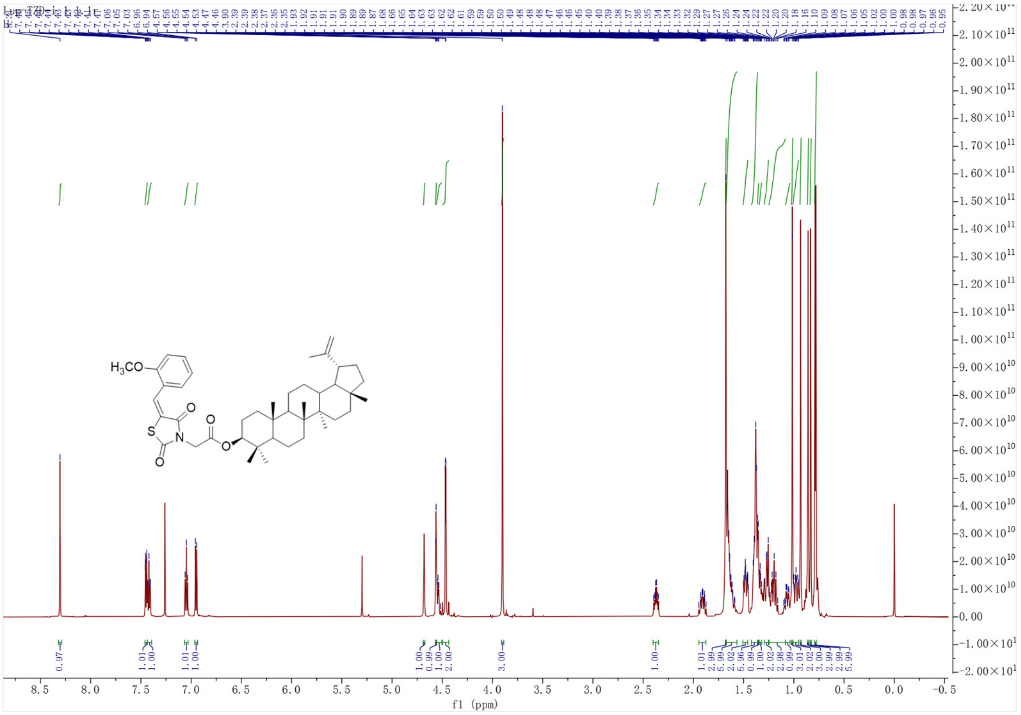

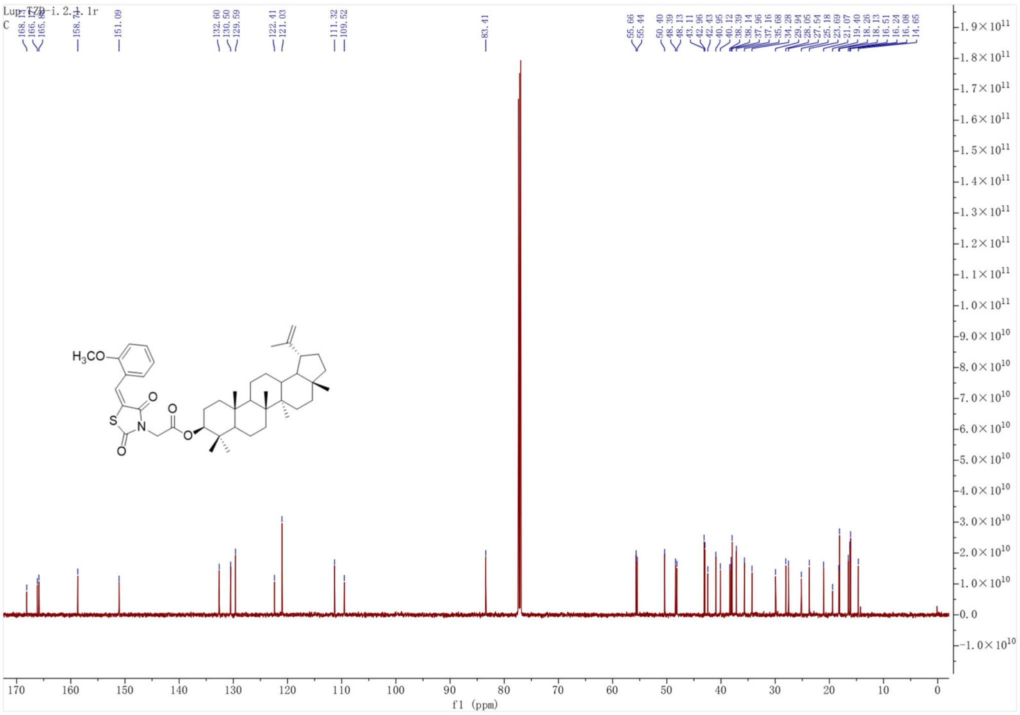

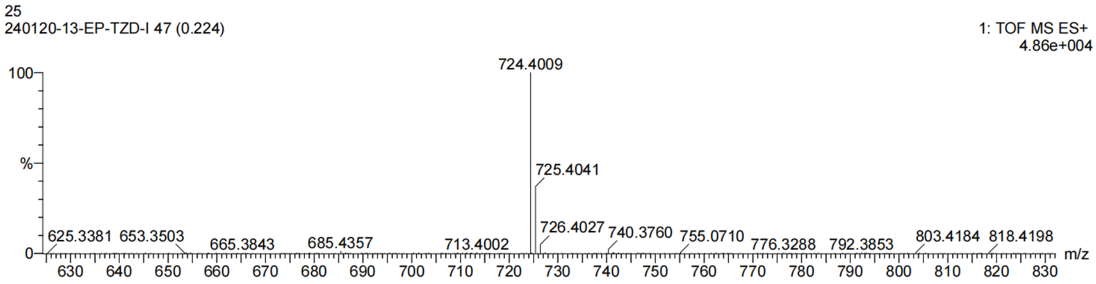

^{1}H NMR, ^{13}C NMR and ESI-HRMS spectra of target compound **6f**

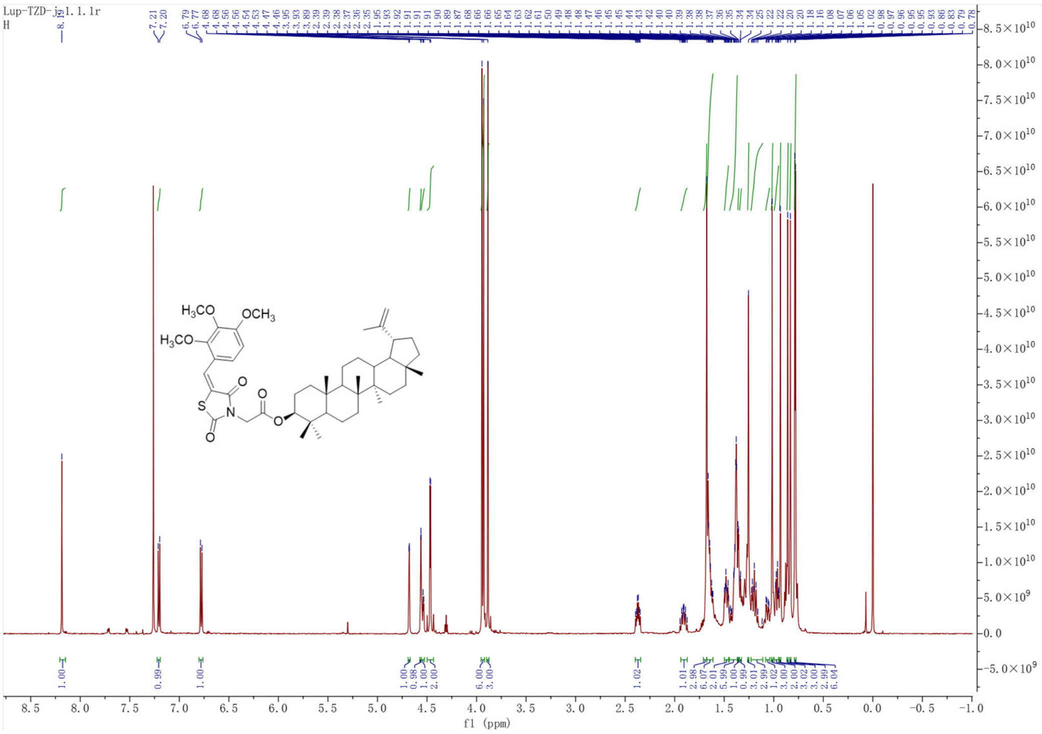

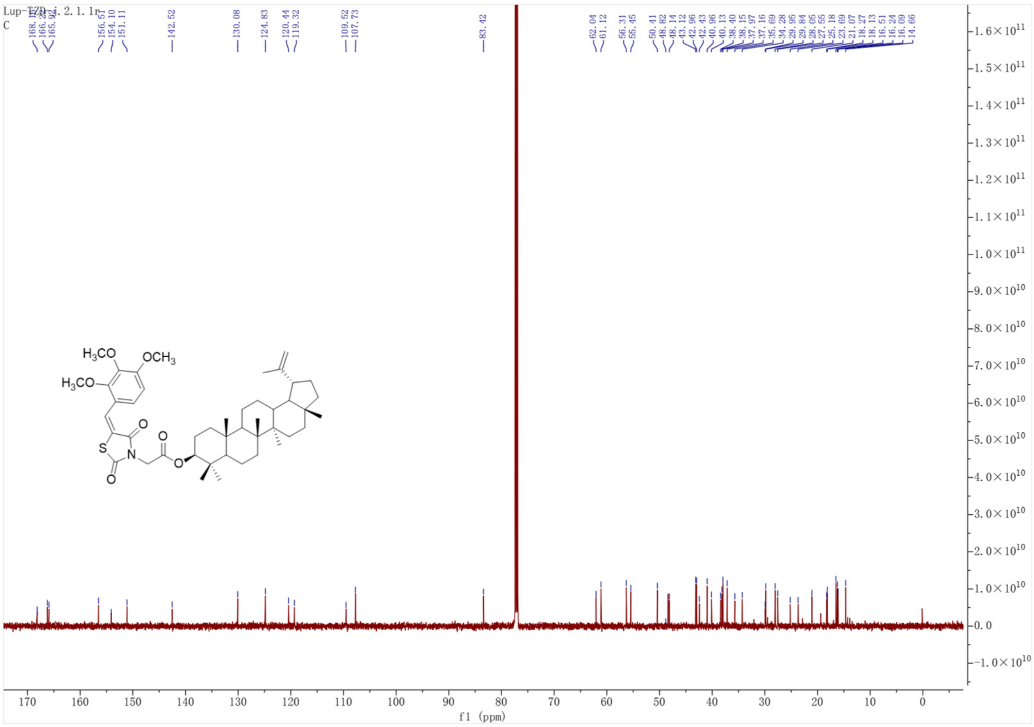

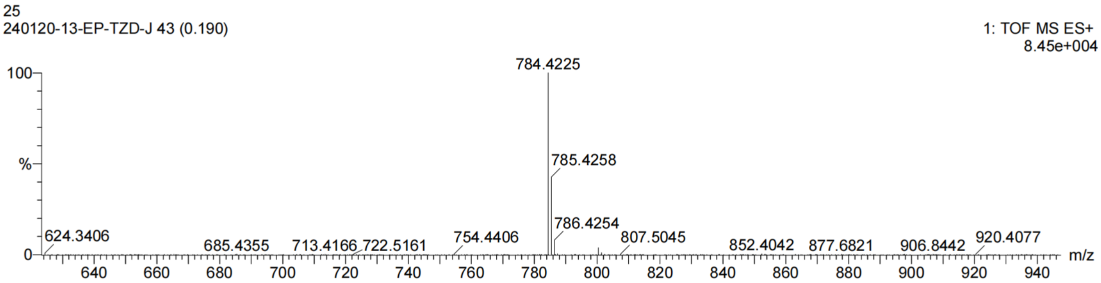

^{1}H NMR, ^{13}C NMR and ESI-HRMS spectra of target compound ***6g***

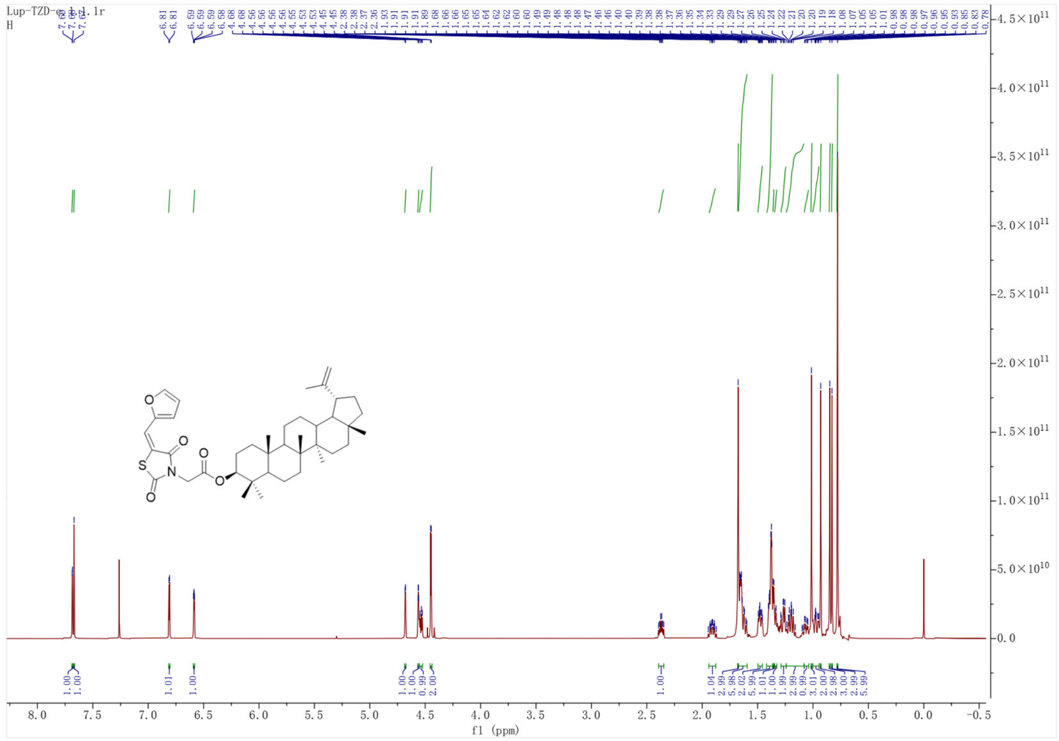

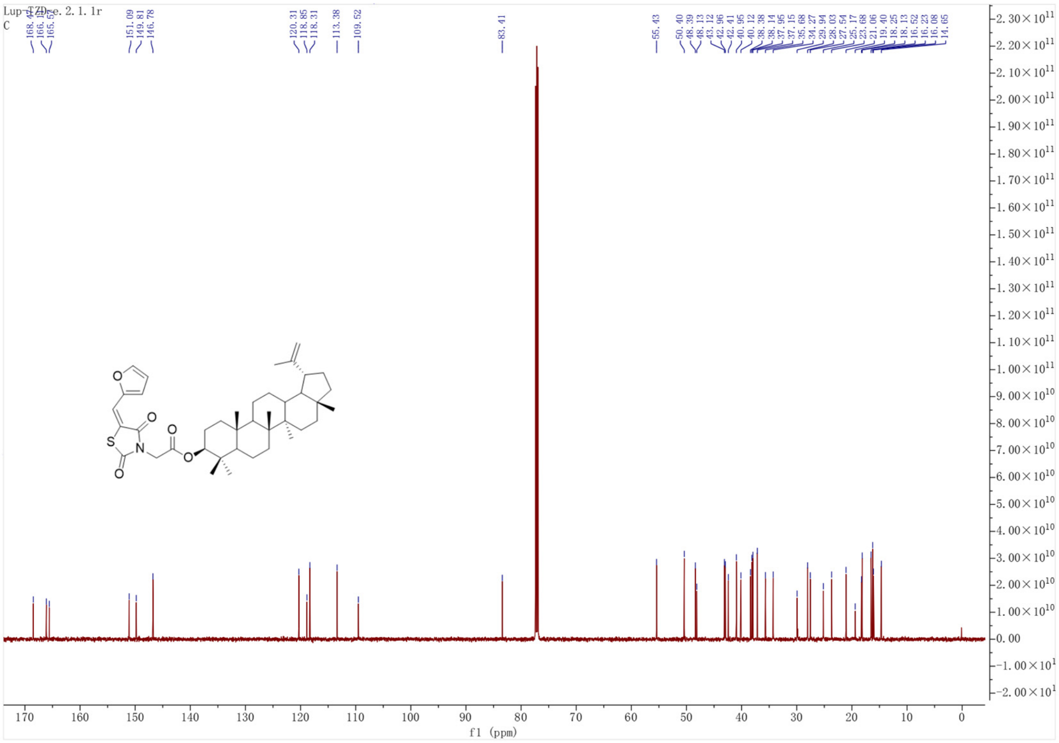

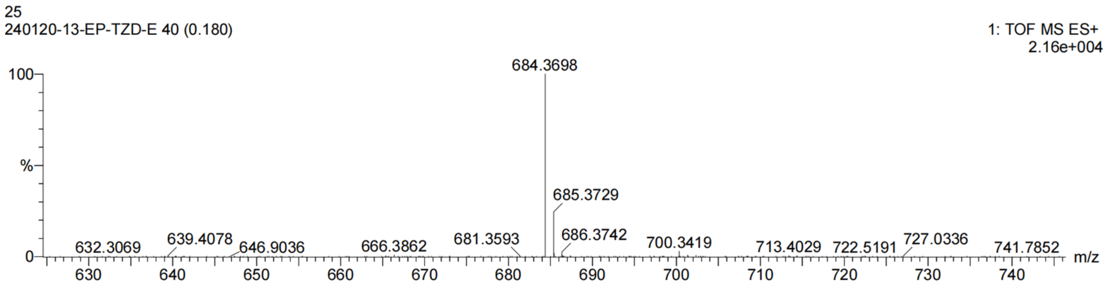

*^{1}H NMR, ^{13}C NMR and ESI-HRMS spectra of target compound **6h***

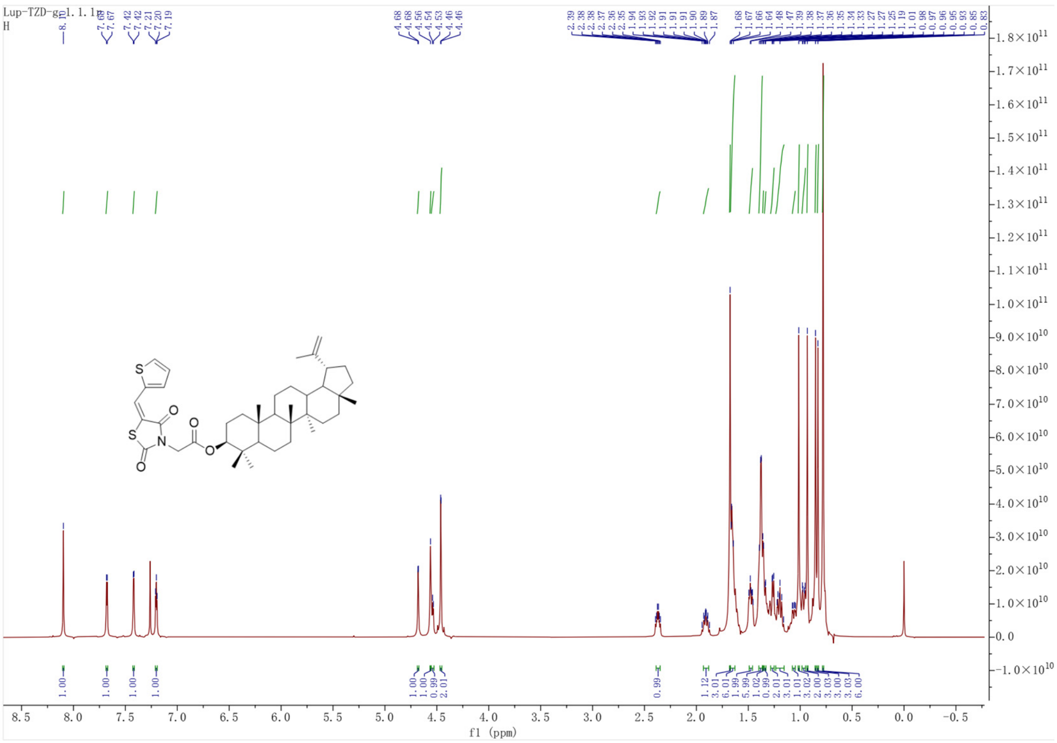

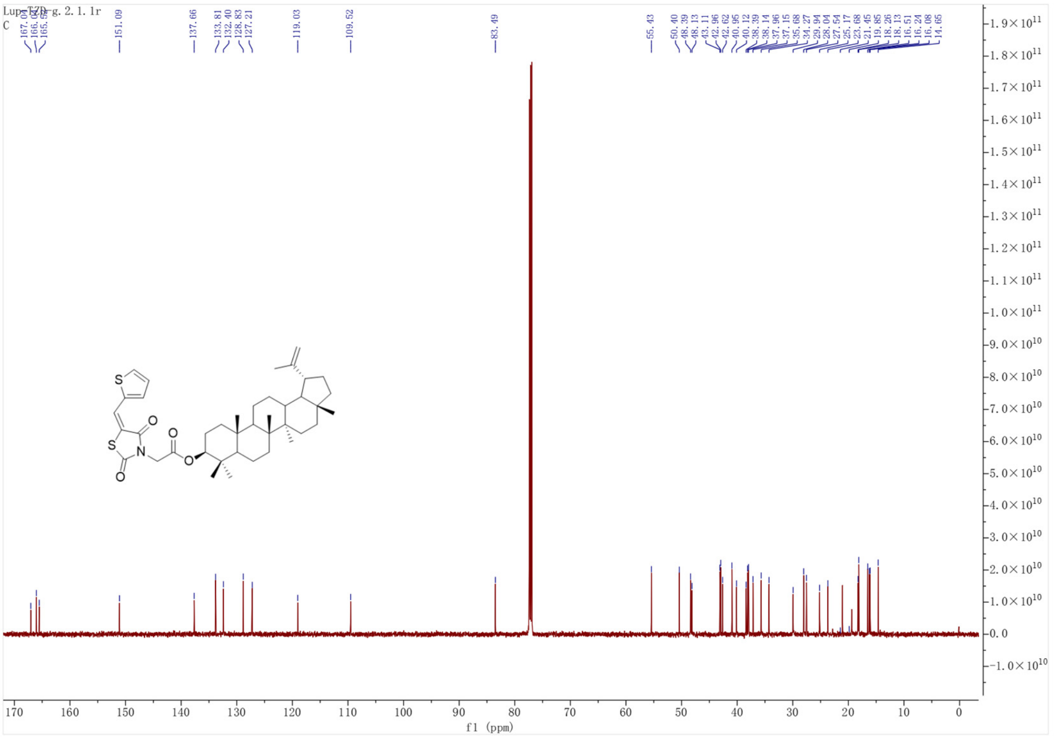

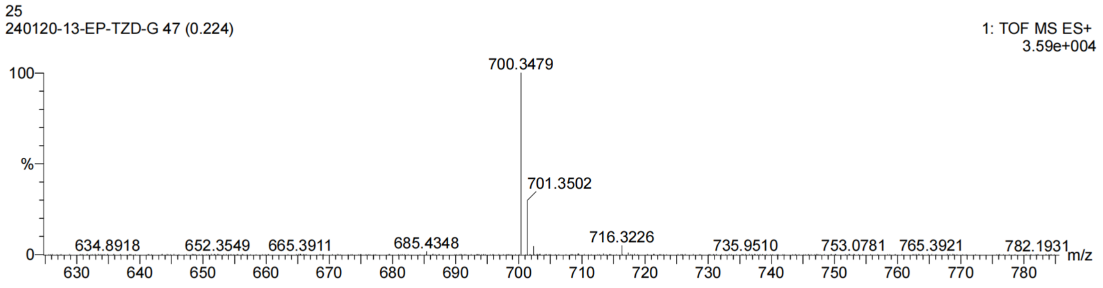

^{1}H NMR, ^{13}C NMR and ESI-HRMS spectra of target compound ***6i***

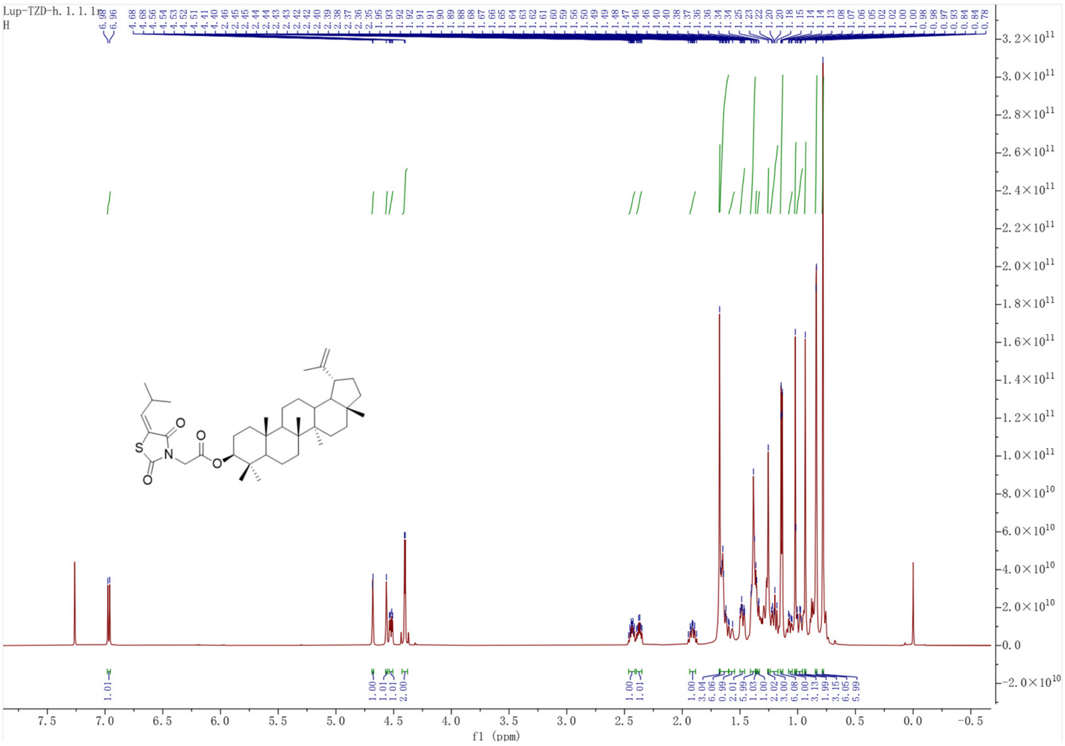

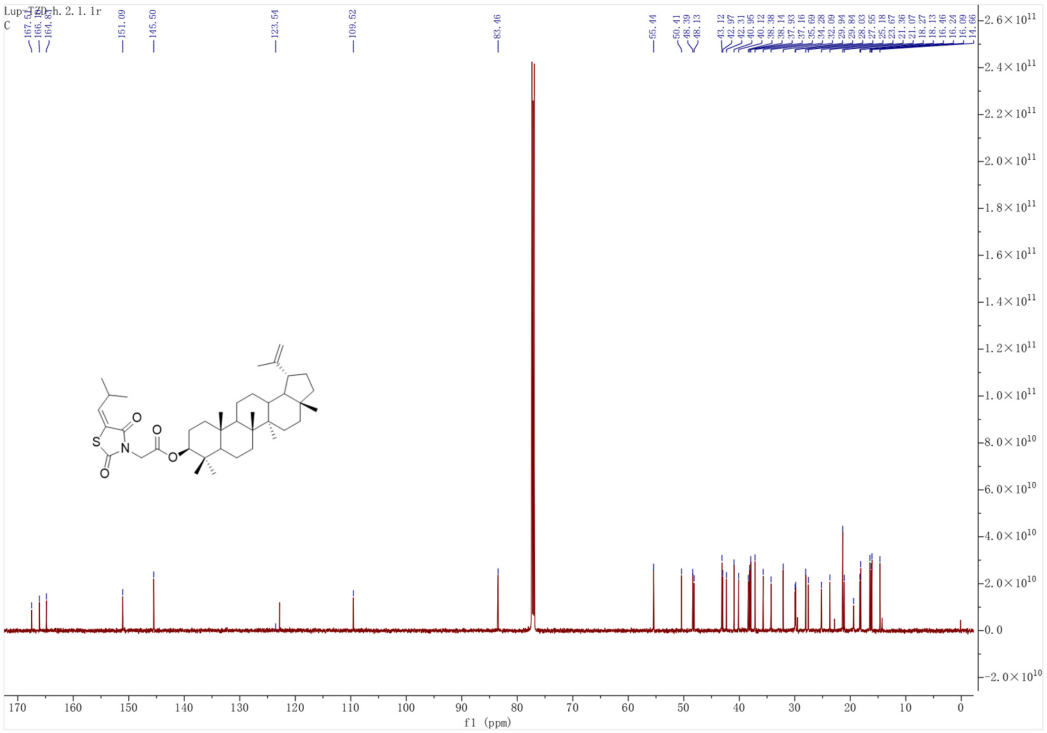

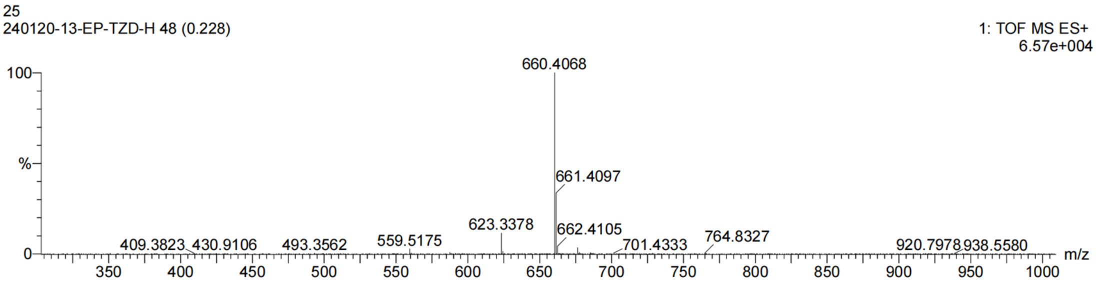

^{1}H NMR and ^{13}C NMR spectra of intermediate ***7***

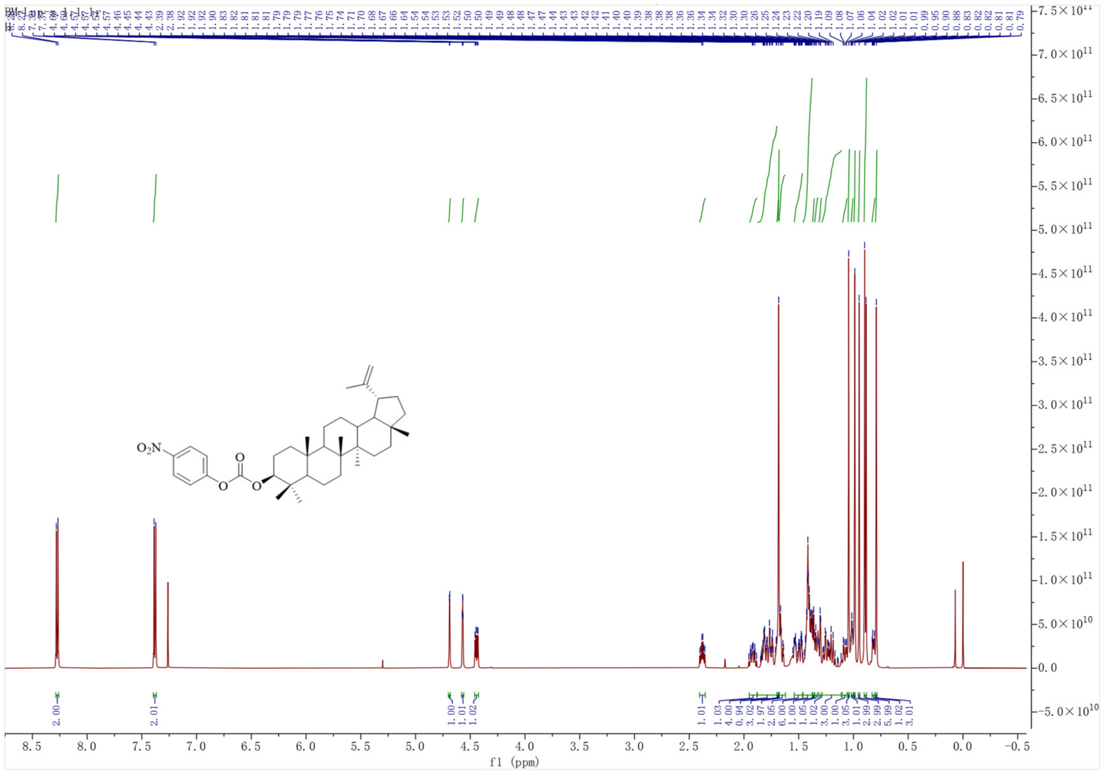

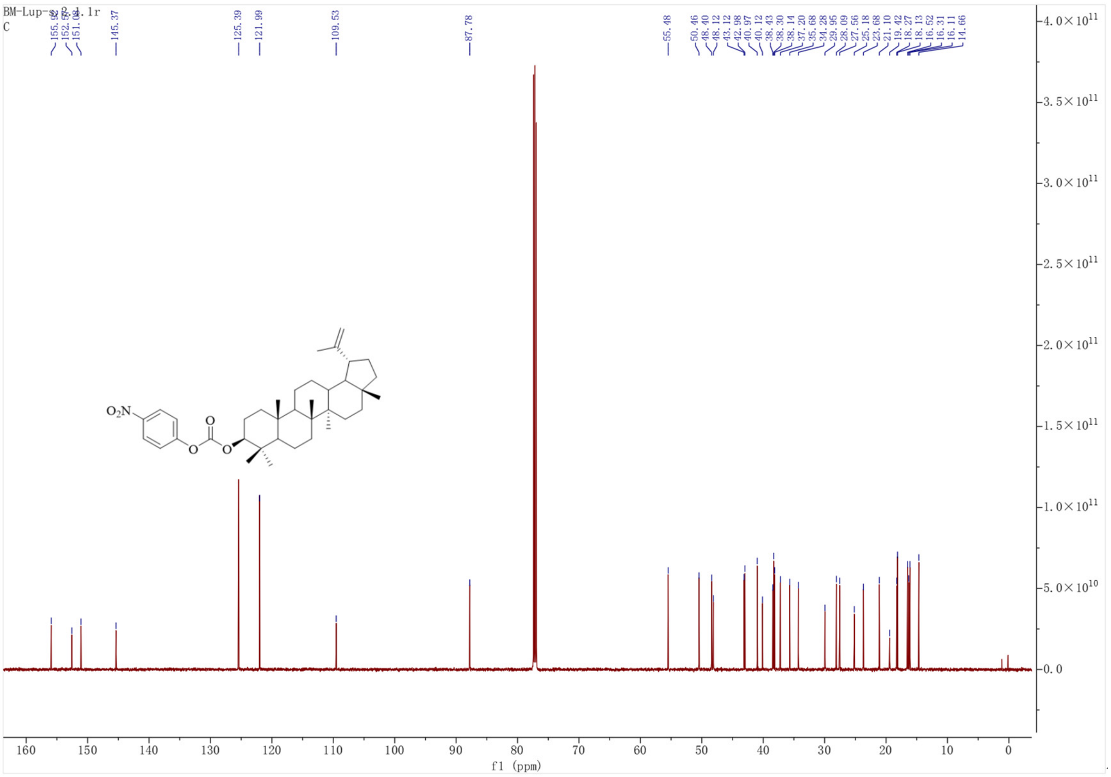

^{1}H NMR and ^{13}C NMR spectra of intermediate ***8***

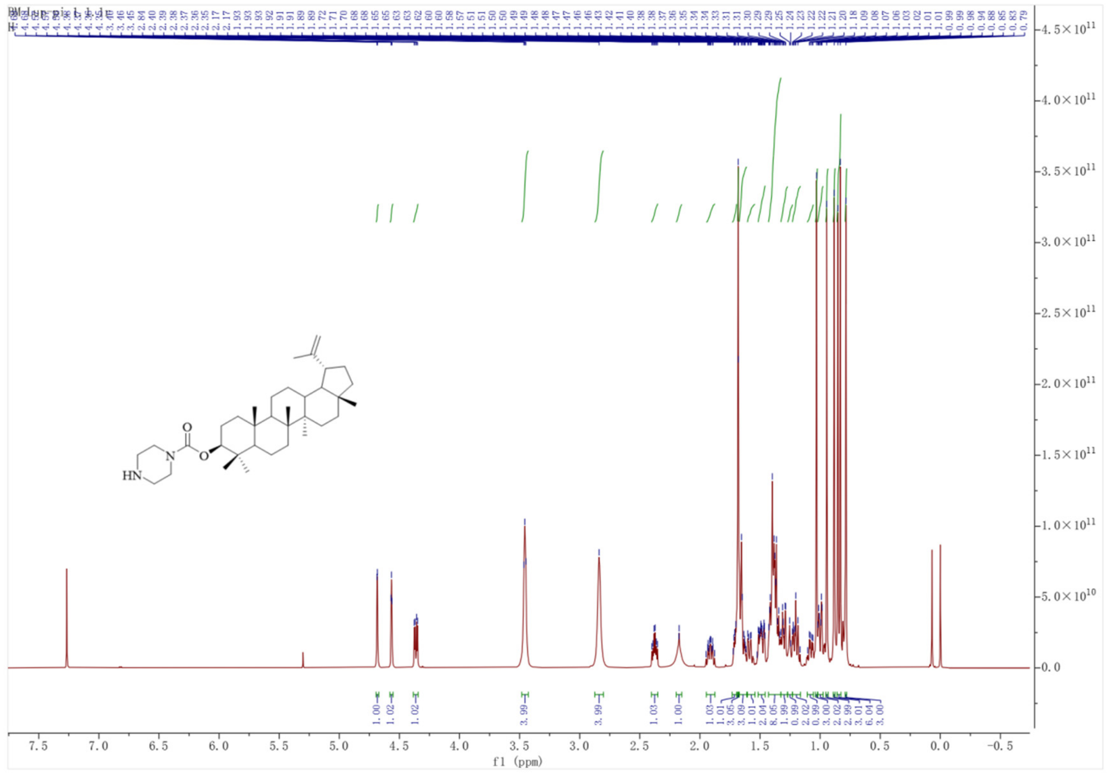

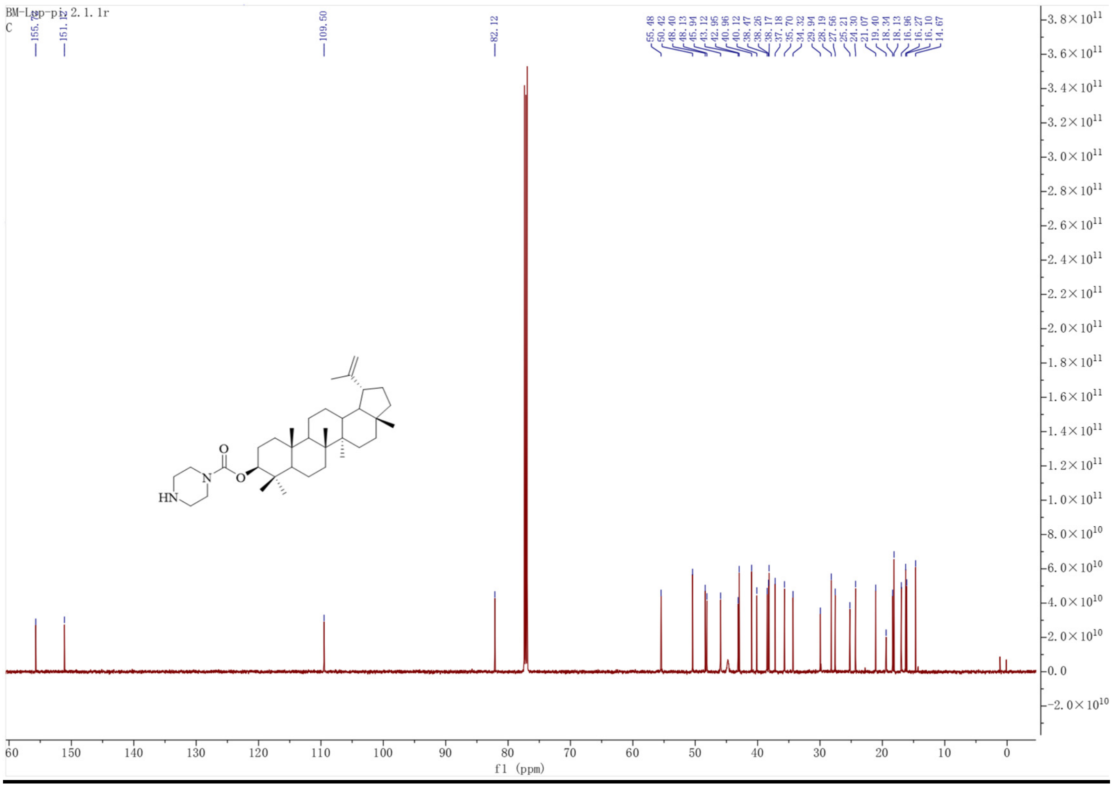

*^{1}H NMR, ^{13}C NMR and ESI-HRMS spectra of target compound **9a***

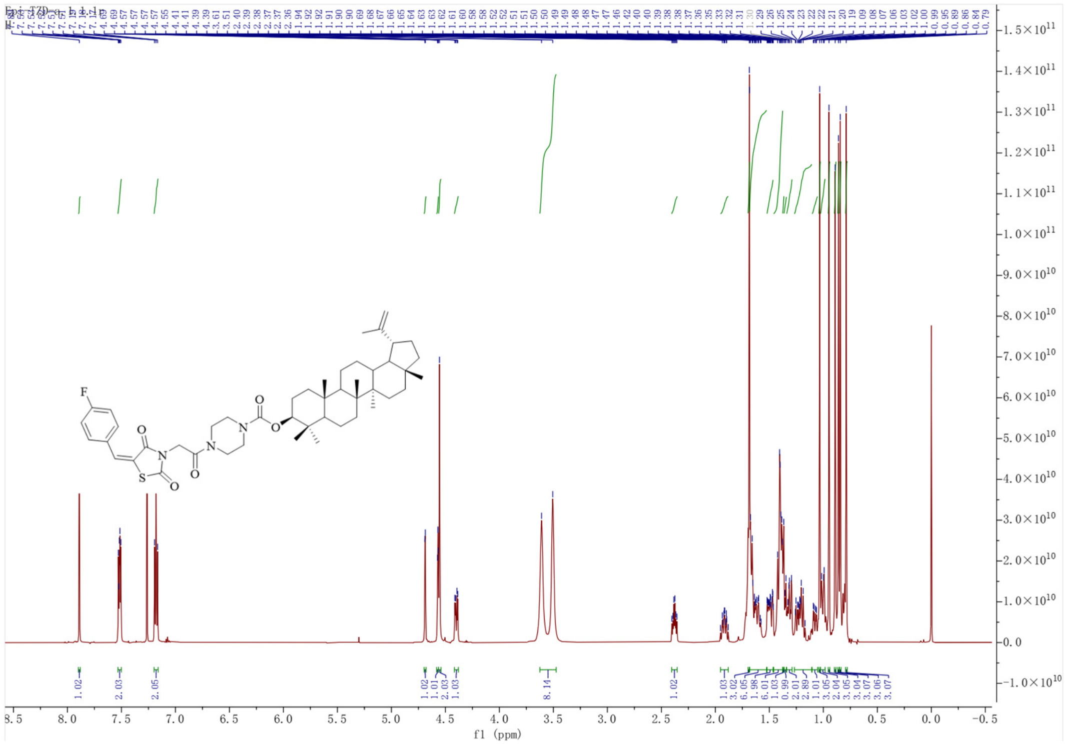

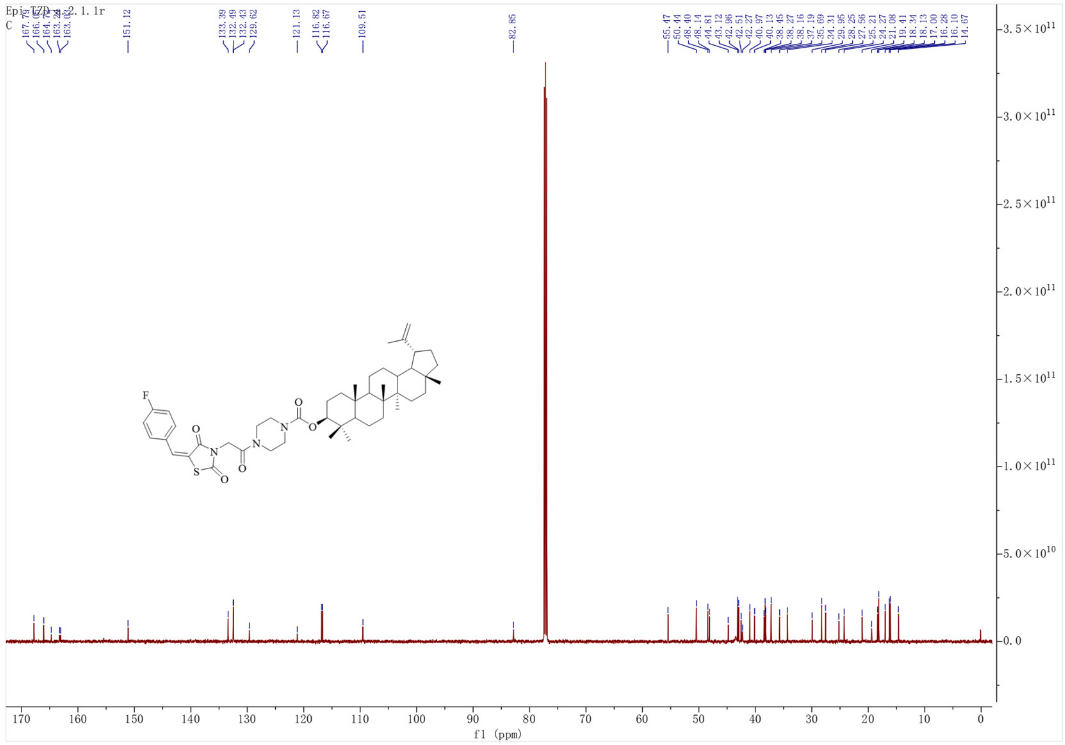

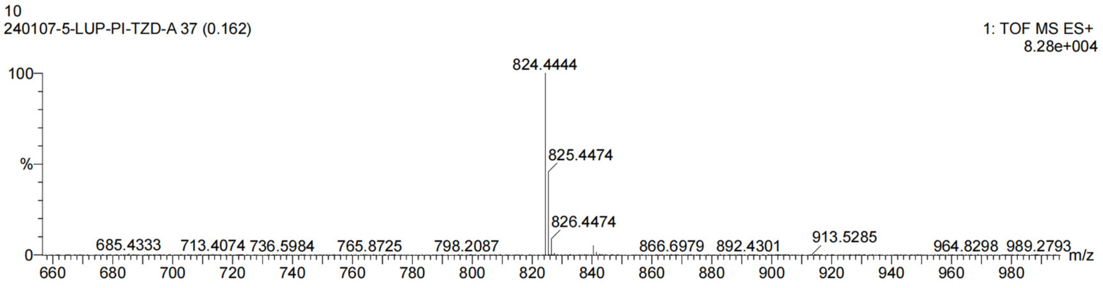

*^{1}H NMR, ^{13}C NMR and ESI-HRMS spectra of target compound **9b***

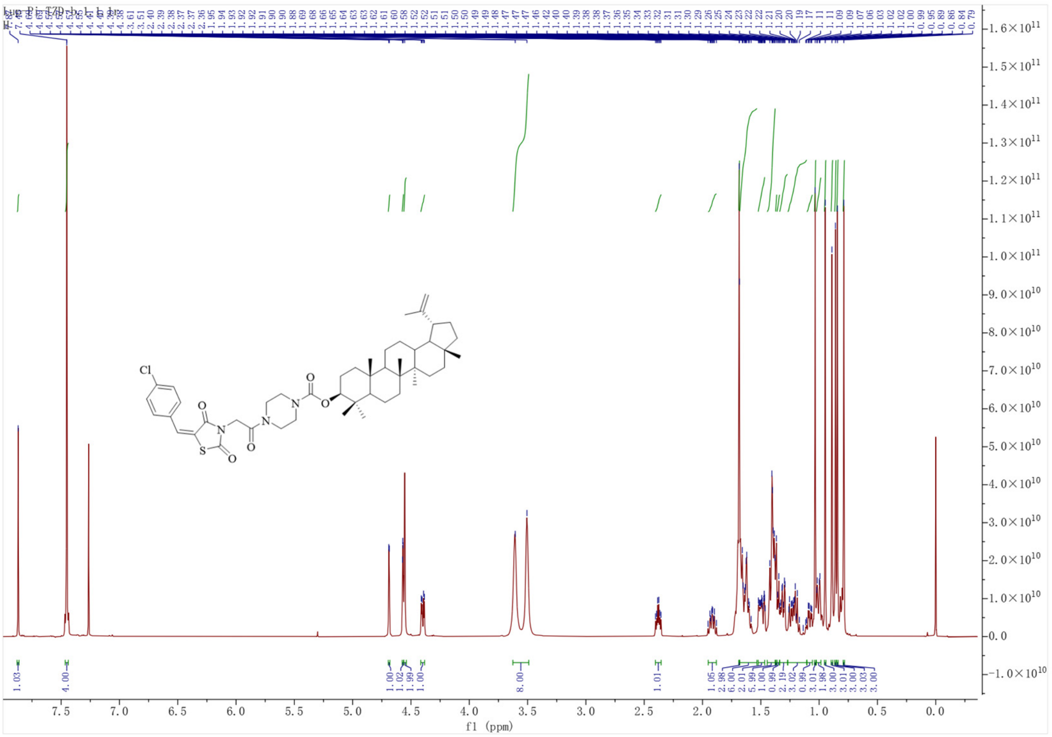

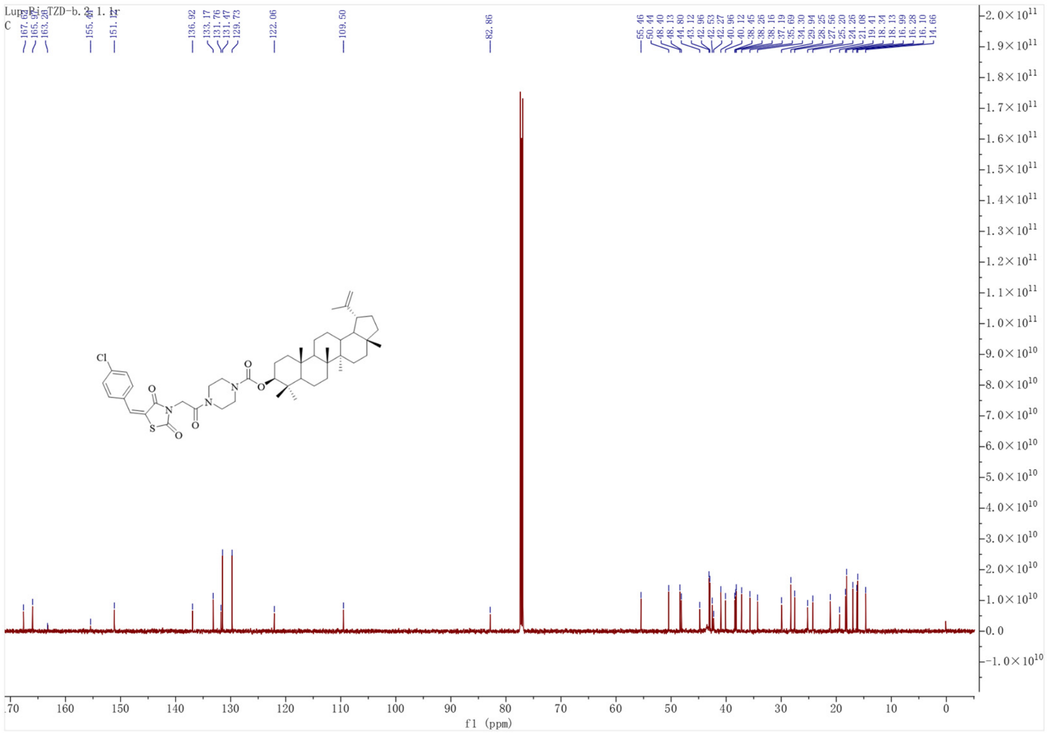

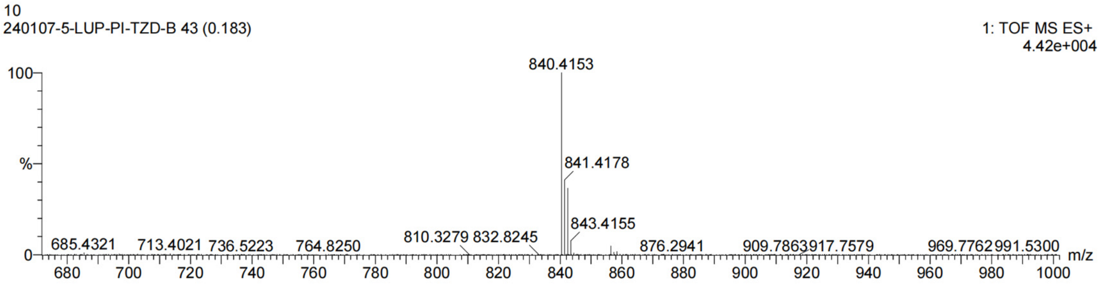

^{1}H NMR, ^{13}C NMR and ESI-HRMS spectra of target compound ***9c***

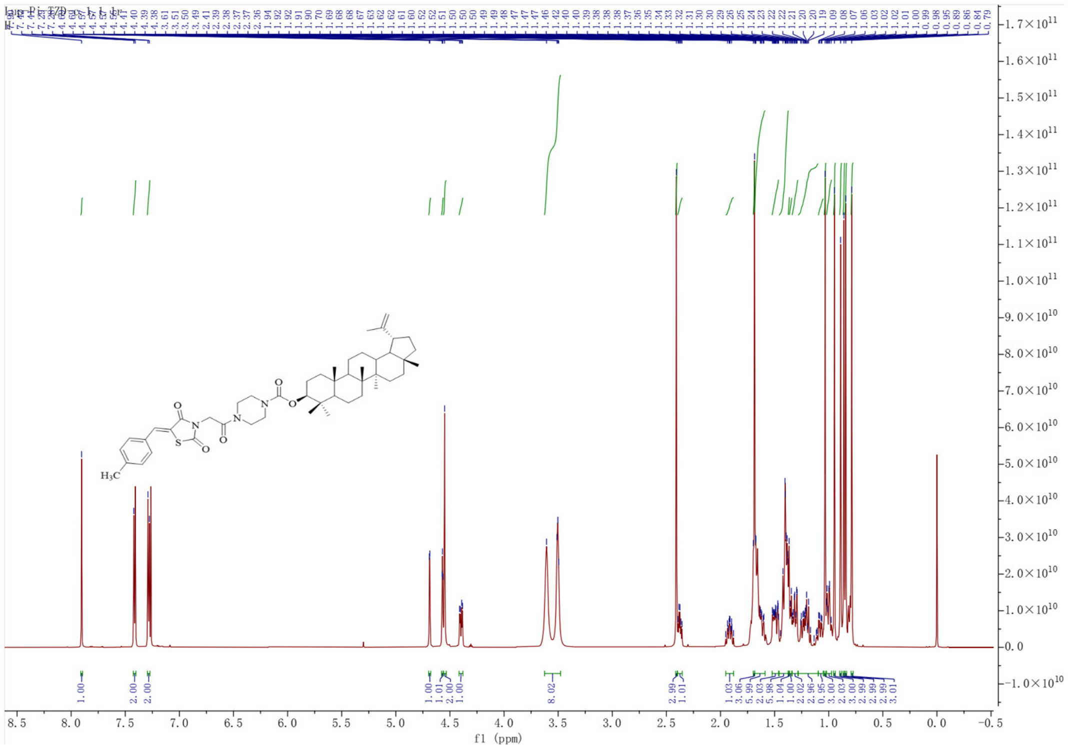

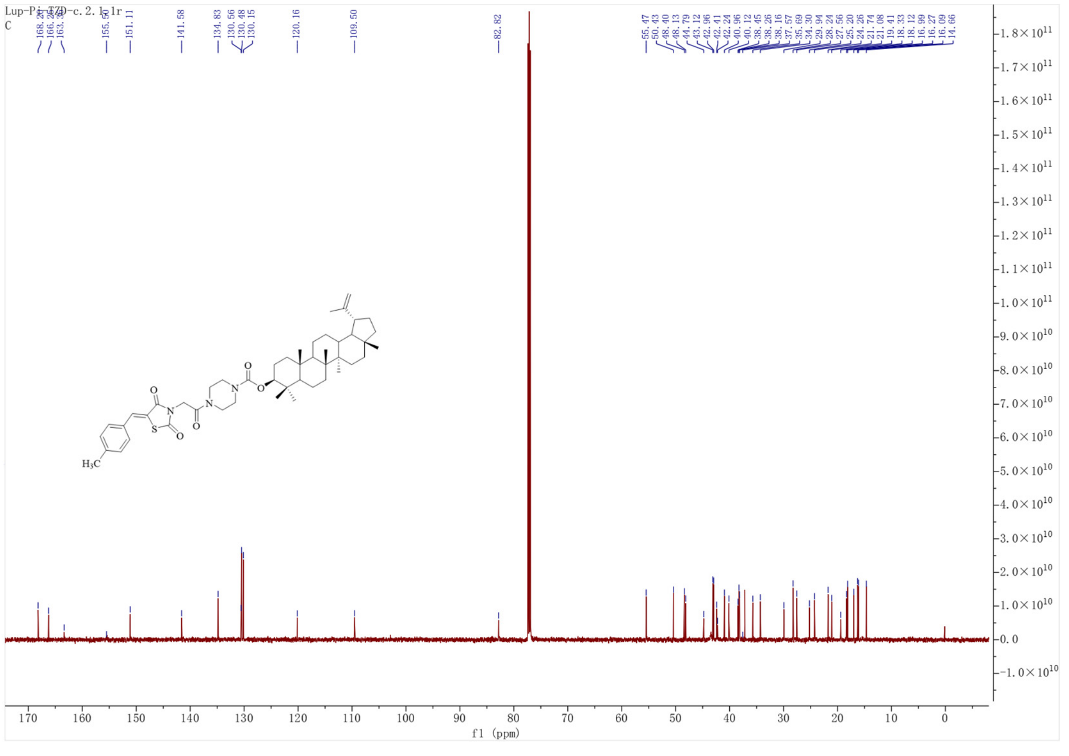

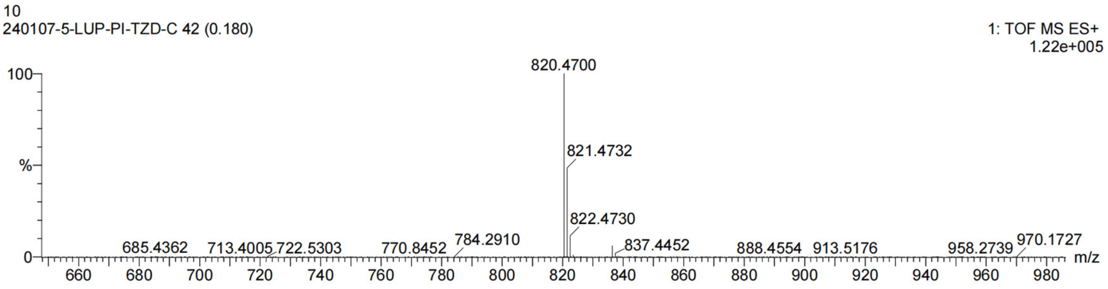

<u>*^{1}H NMR, ^{13}C NMR and ESI-HRMS spectra of target compound **9d***</u>

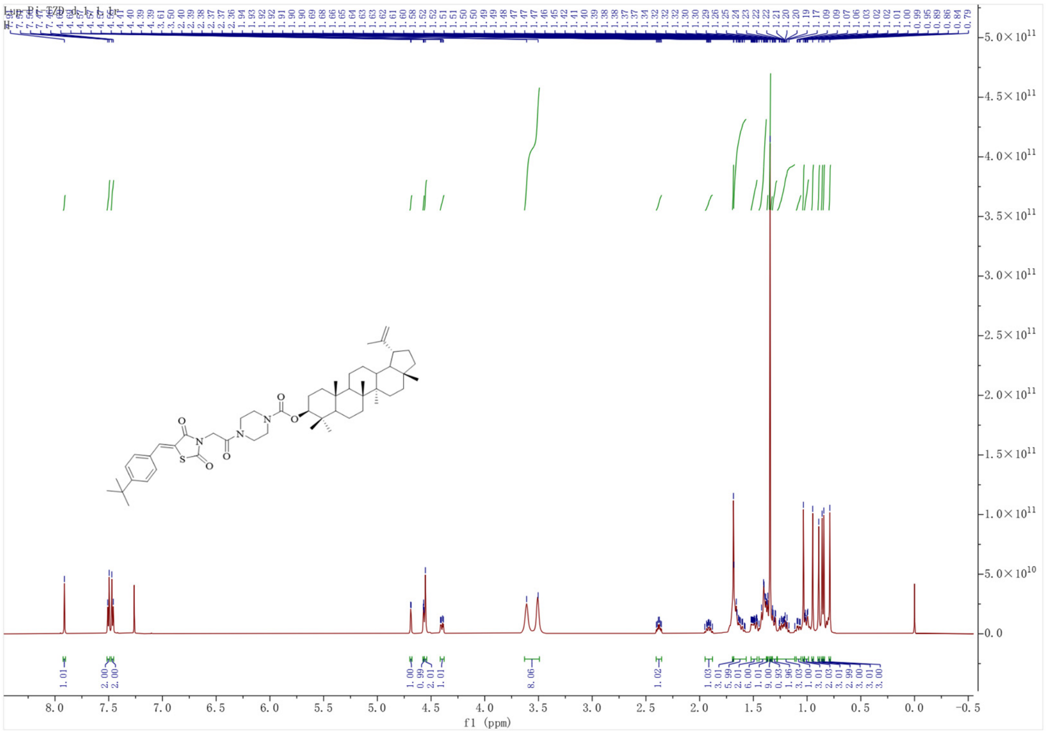

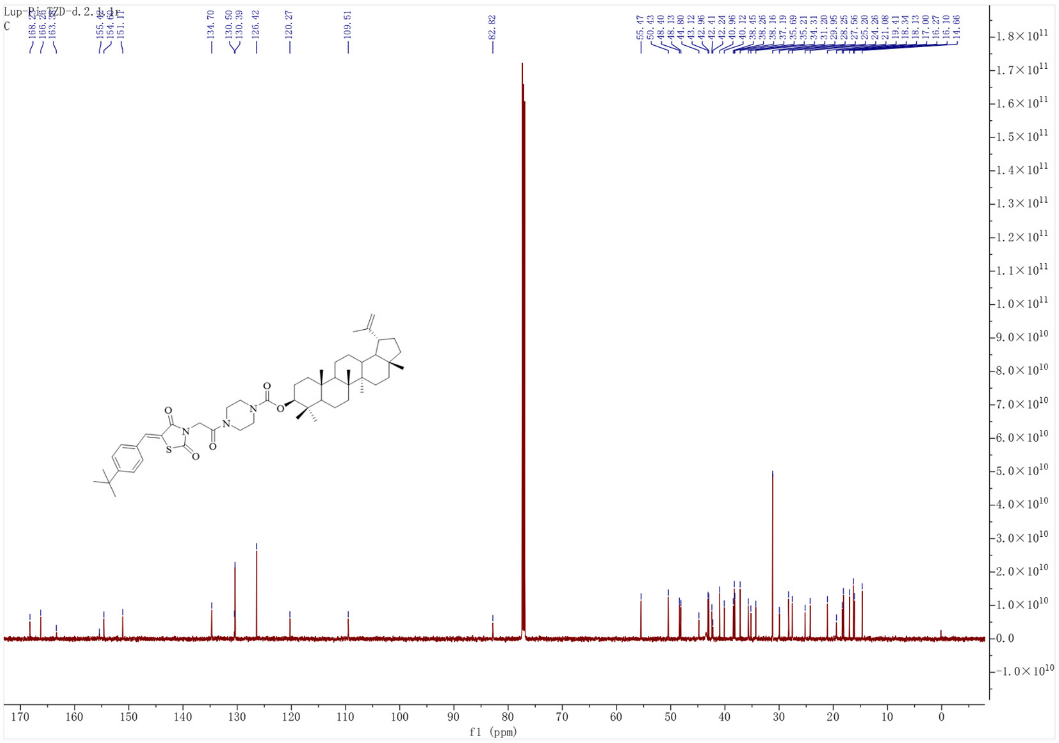

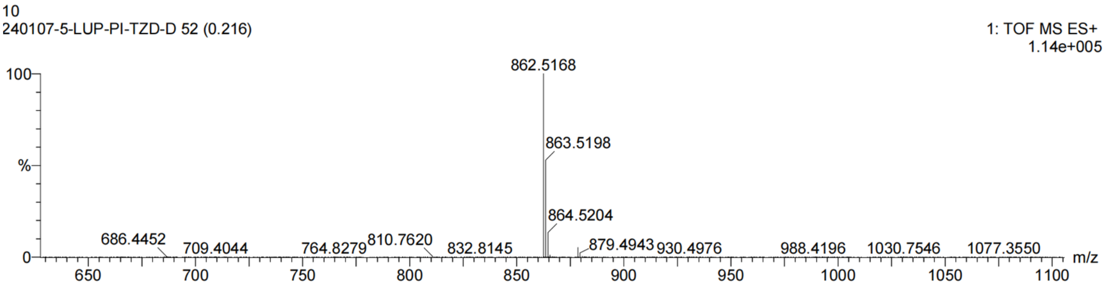

*^{1}H NMR, ^{13}C NMR and ESI-HRMS spectra of target compound **9e***

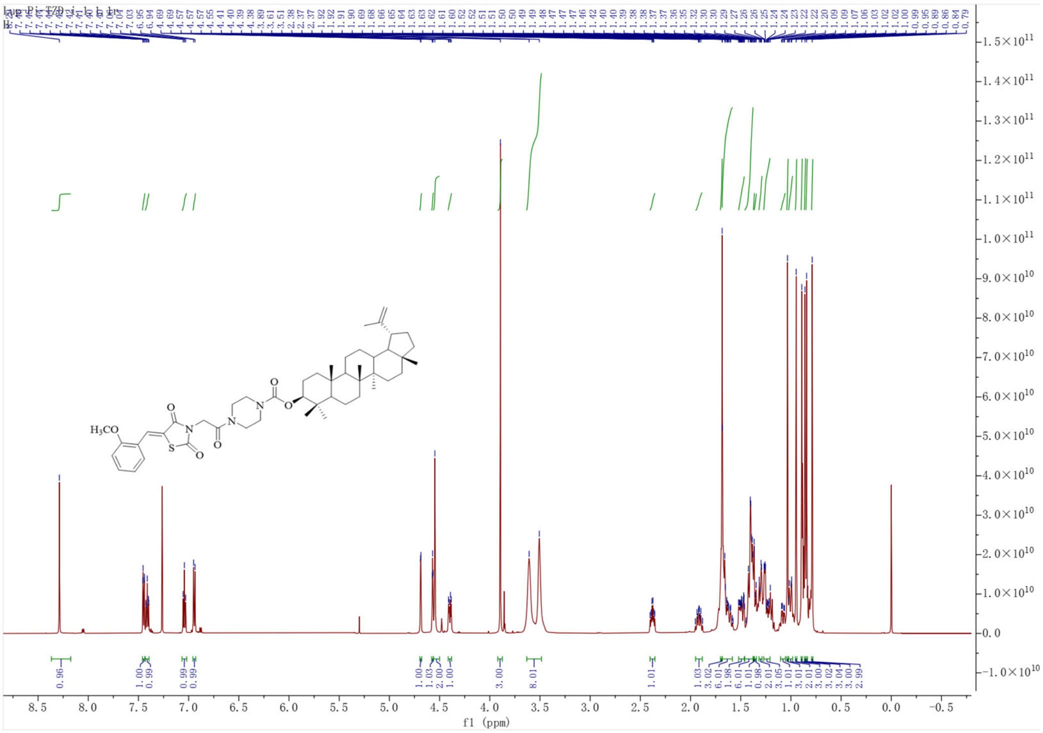

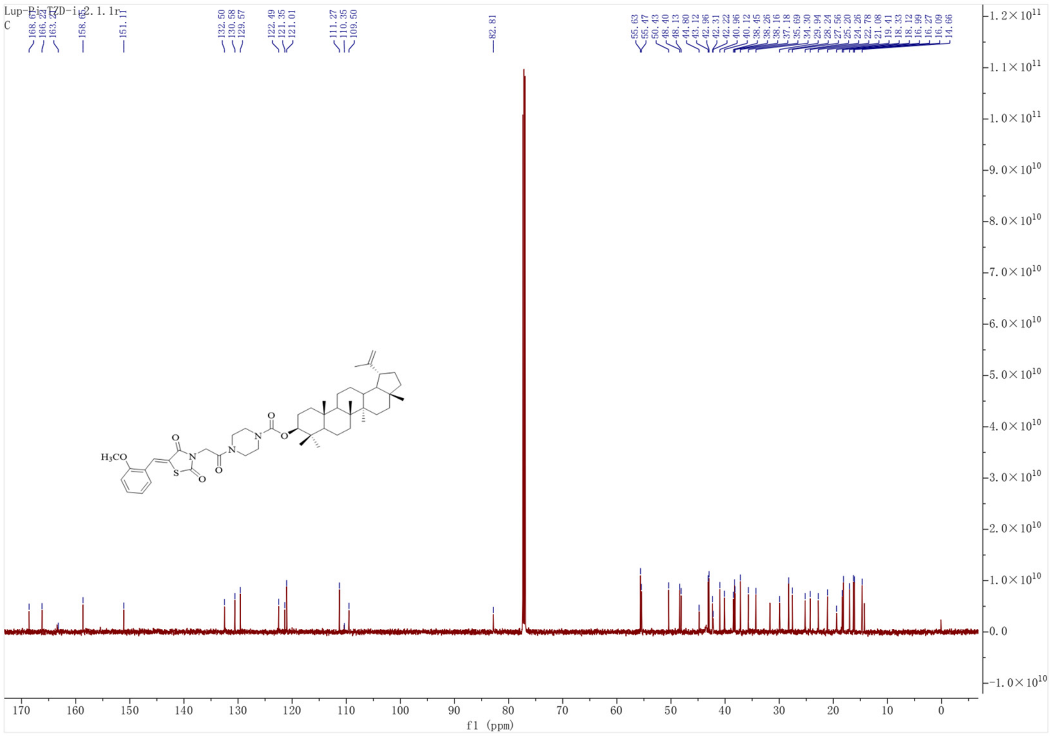

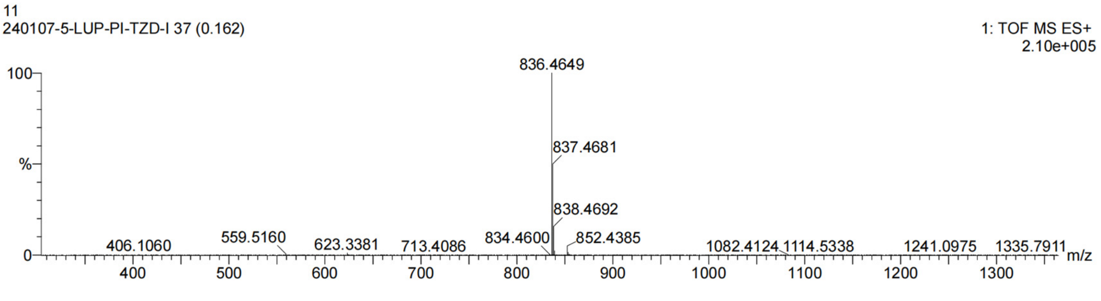

^{1}H NMR, ^{13}C NMR and ESI-HRMS spectra of target compound ***9f***

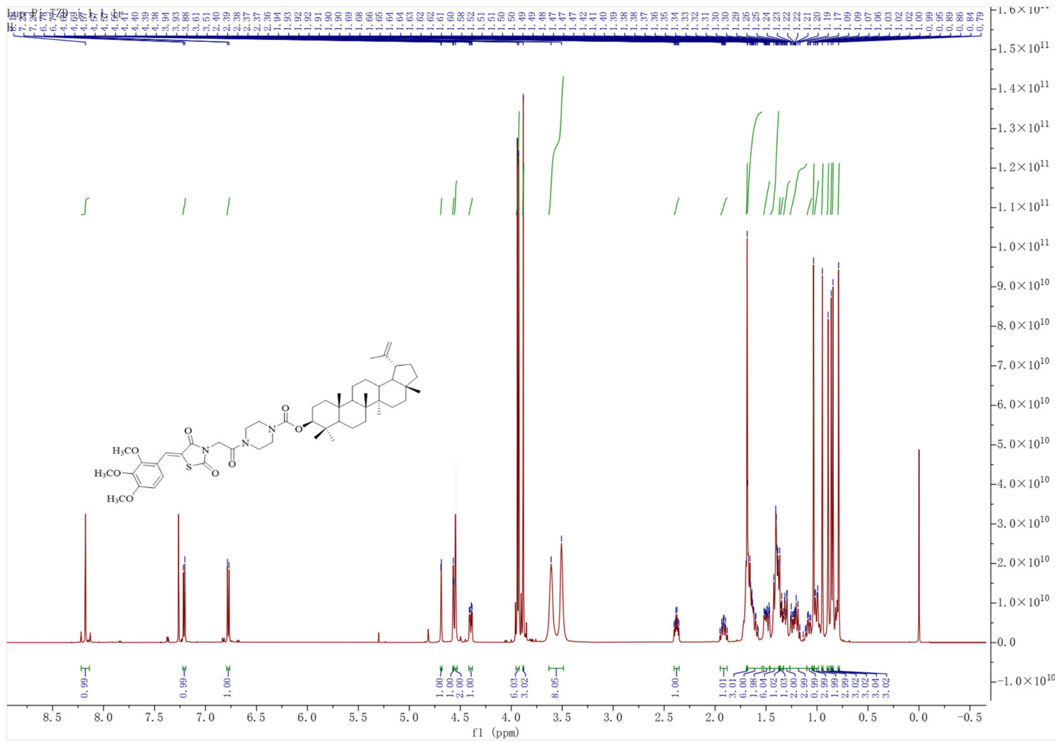

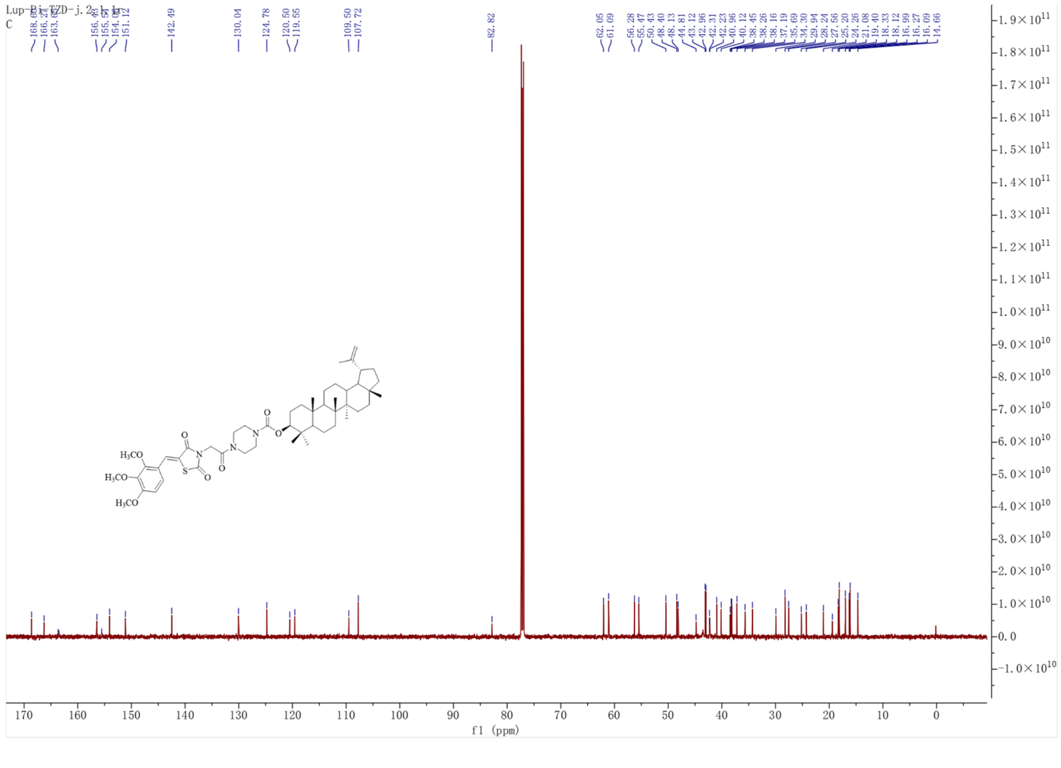

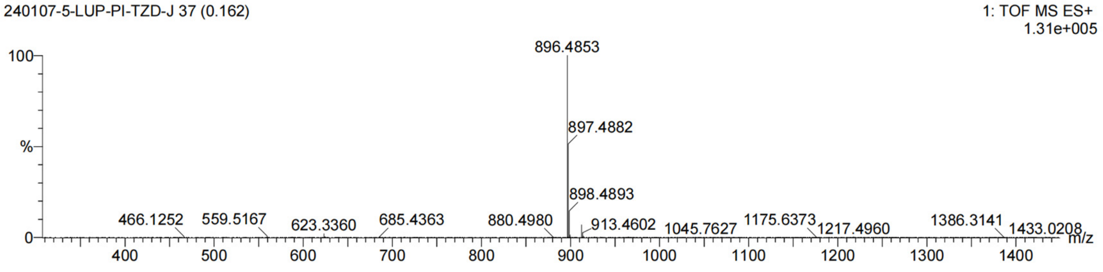

^{1}H NMR, ^{13}C NMR and ESI-HRMS spectra of target compound ***9g***

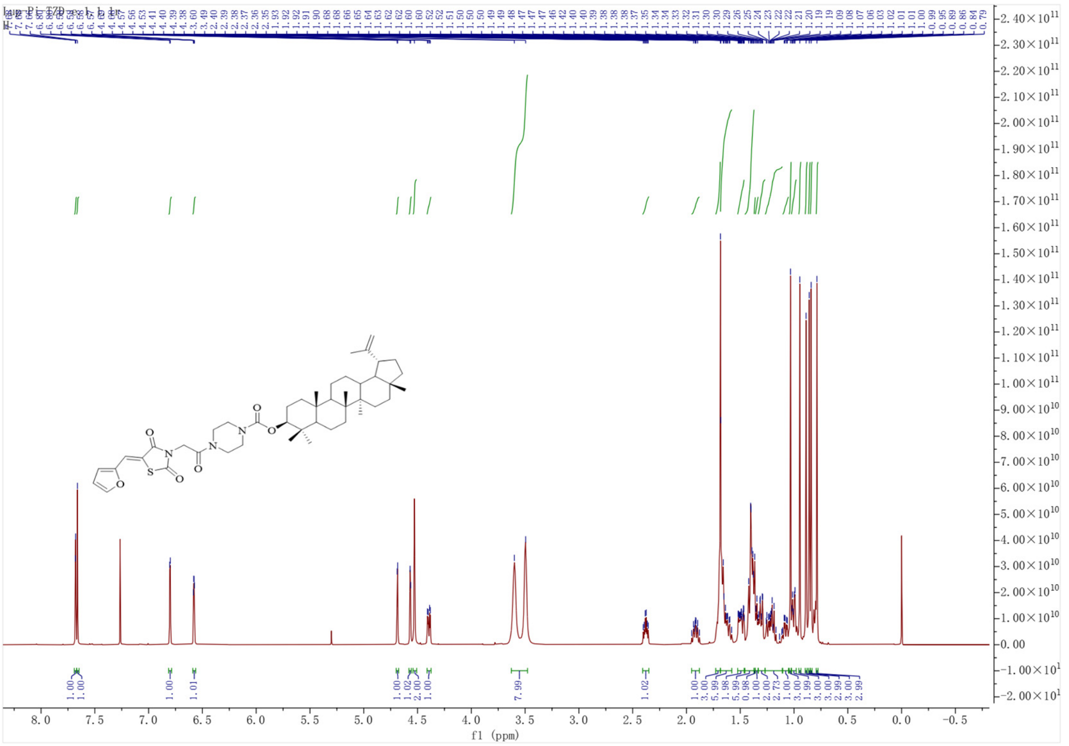

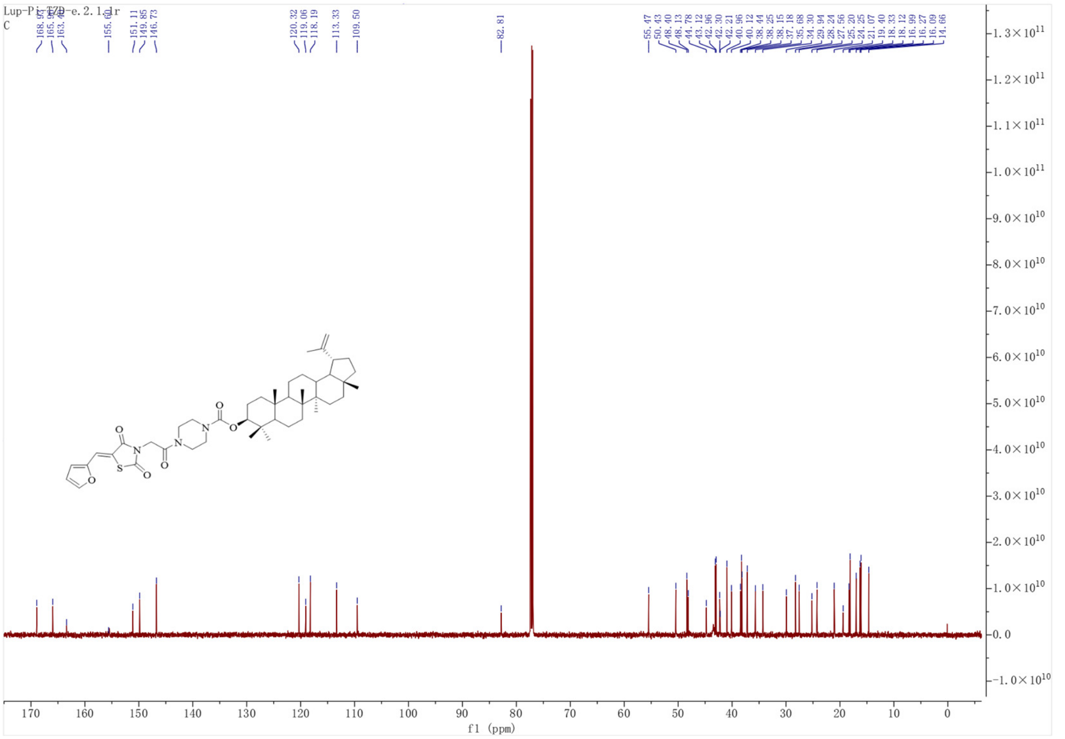

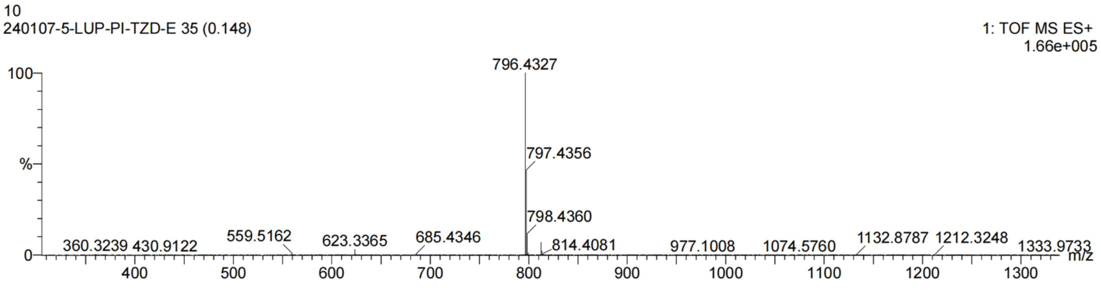

^{1}H NMR, ^{13}C NMR and ESI-HRMS spectra of target compound ***9h***

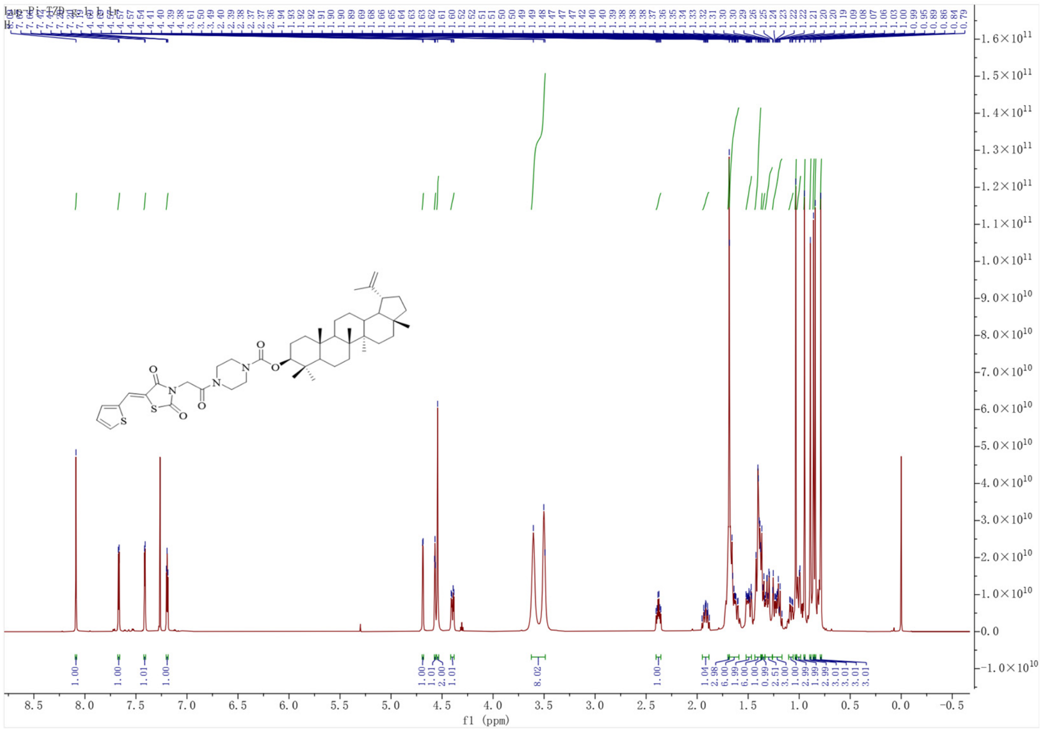

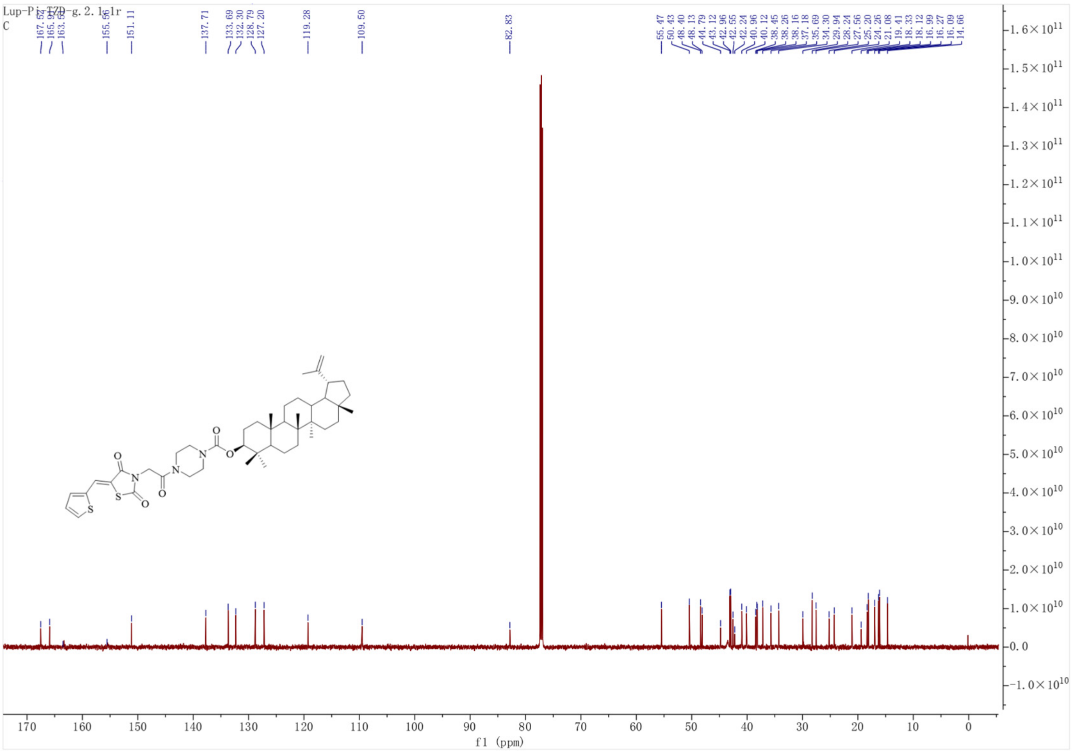

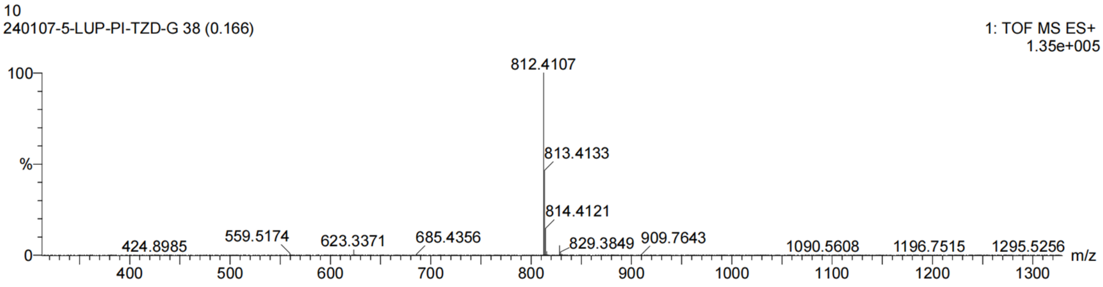

^{1}H NMR, ^{13}C NMR and ESI-HRMS spectra of target compound **9i**

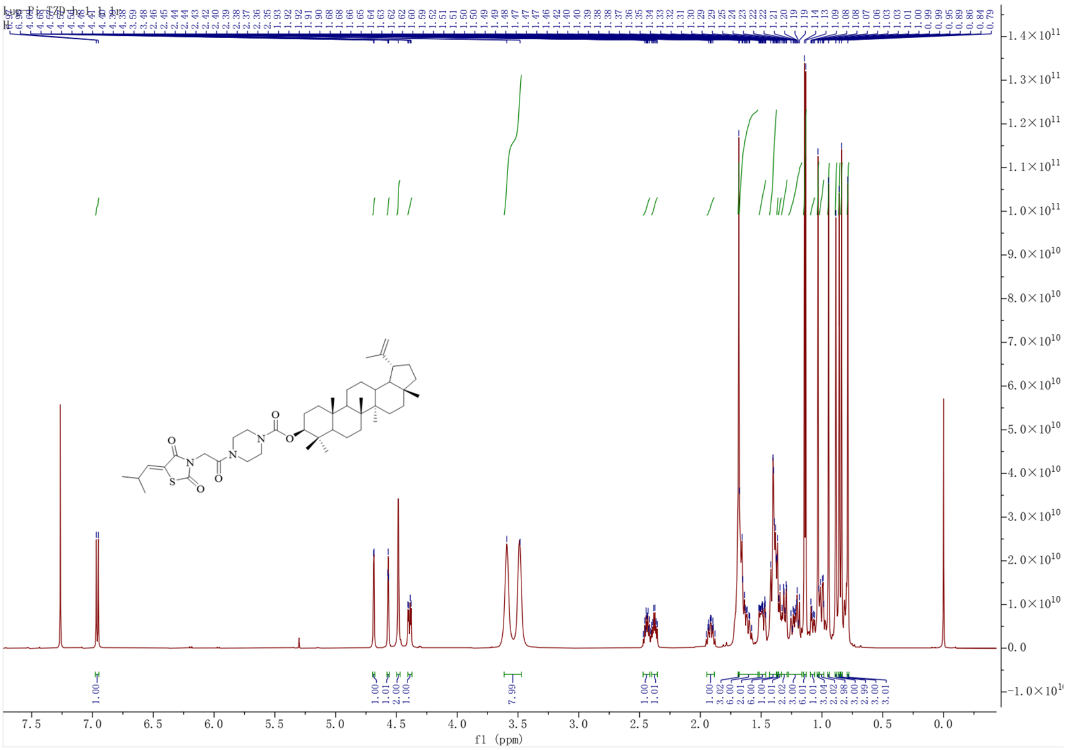

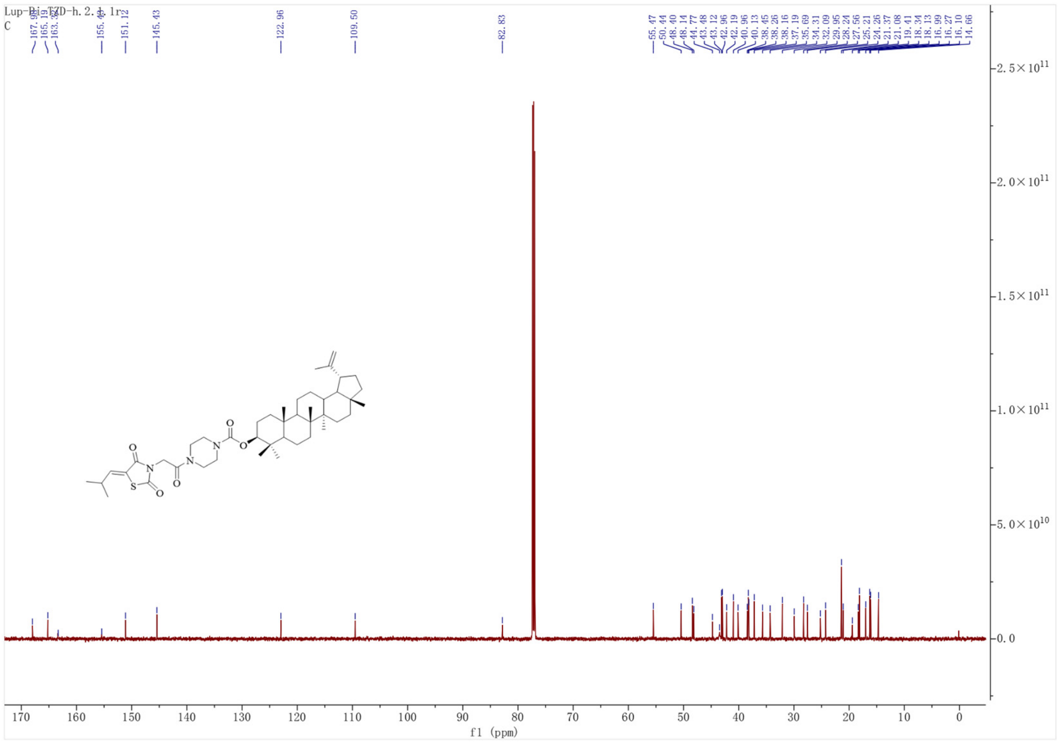

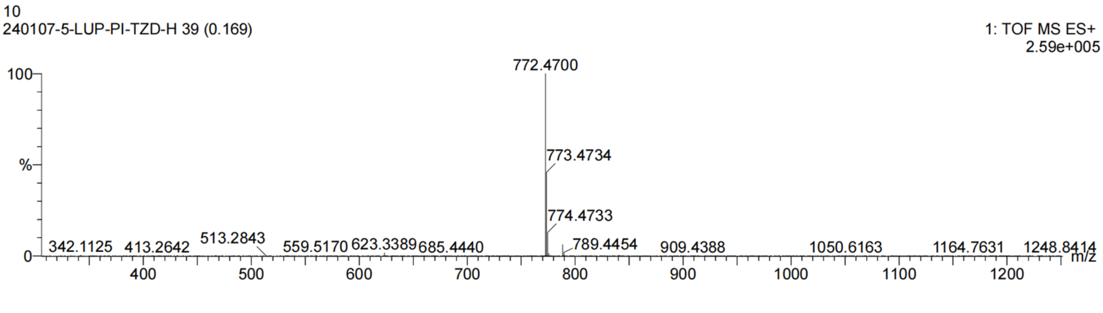

*^{1}H NMR and ^{13}C NMR spectra of intermediate **10***

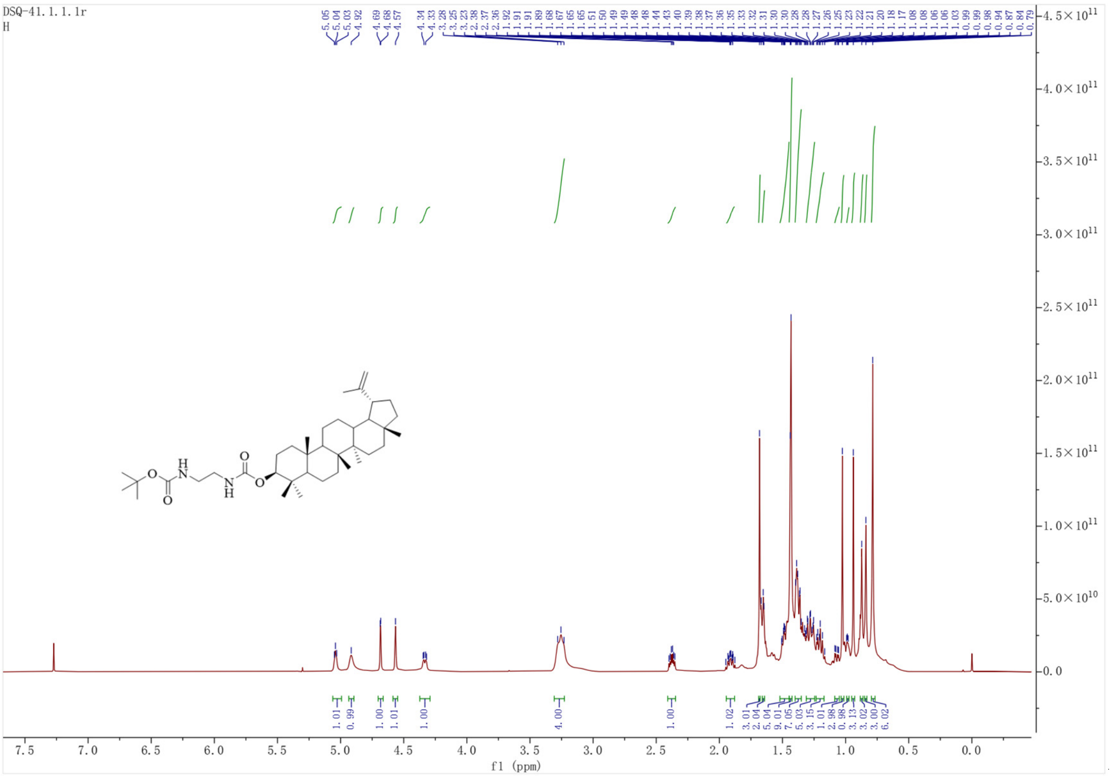

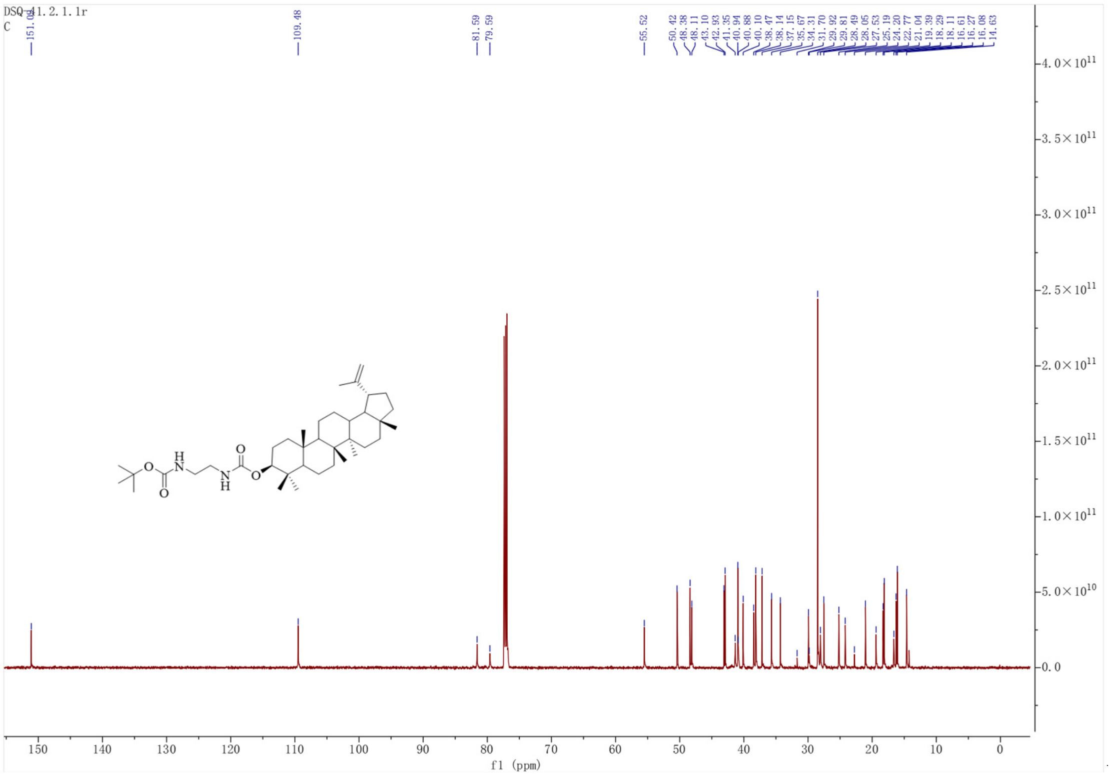

^{1}H NMR, ^{13}C NMR and ESI-HRMS spectra of target compound ***12a***

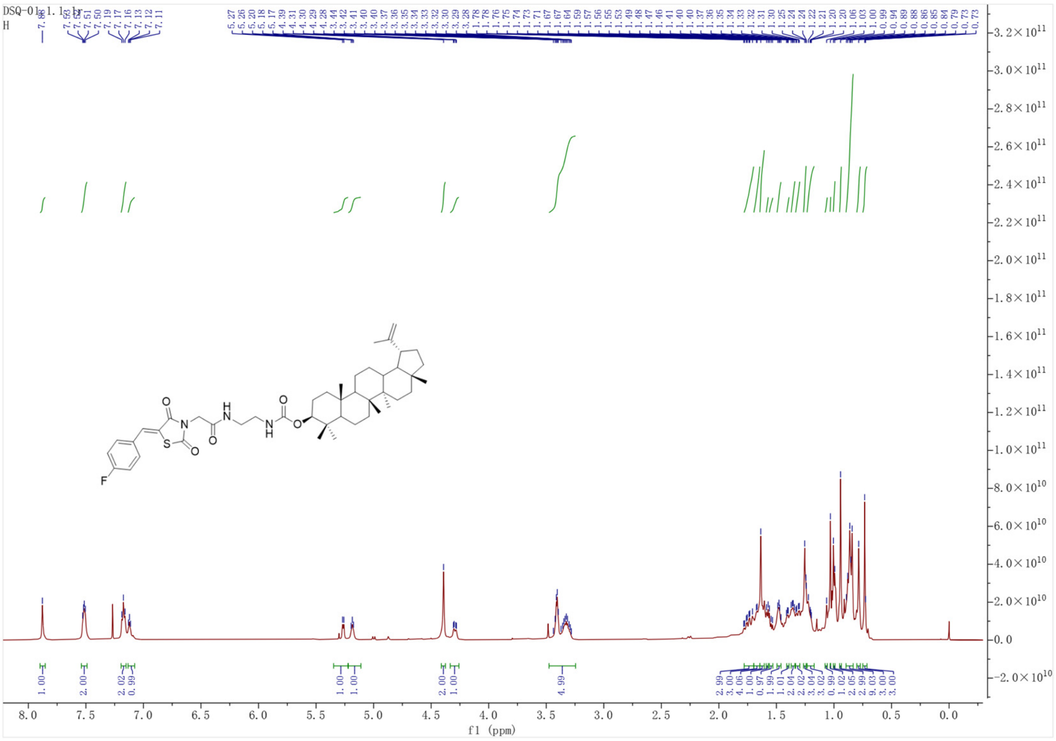

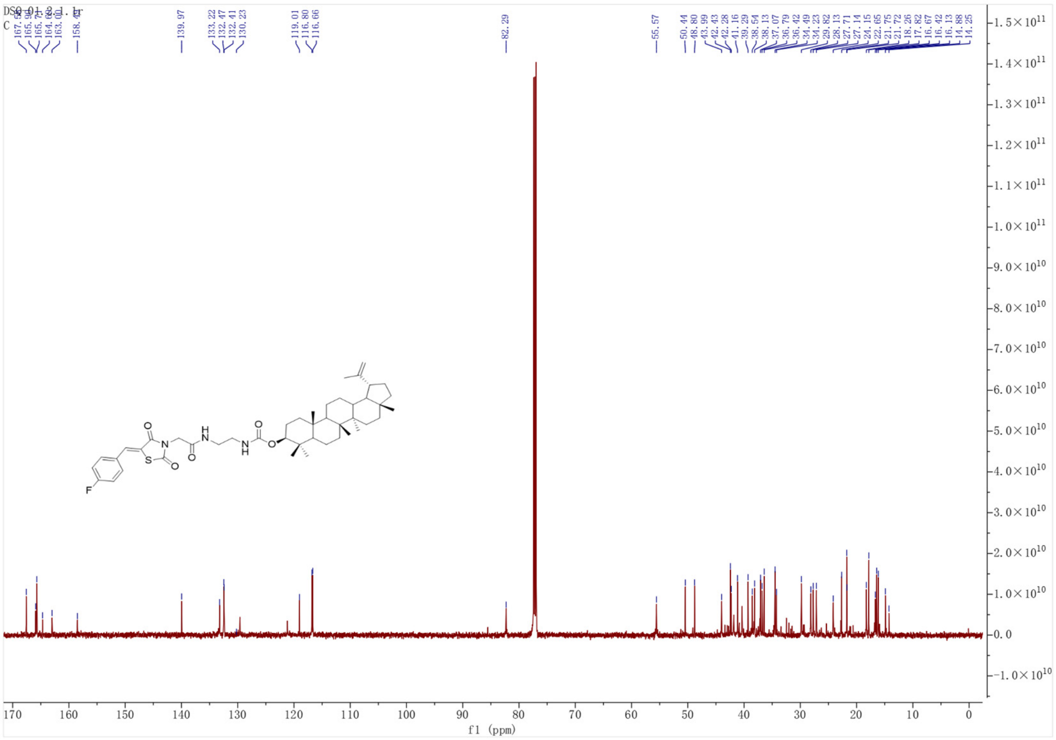

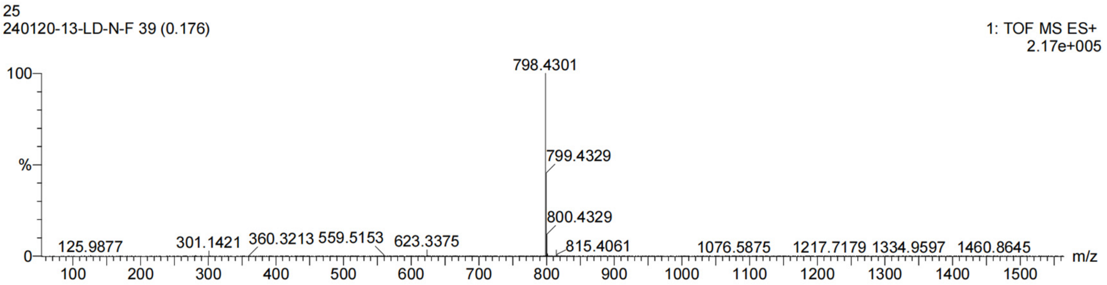

^{1}H NMR, ^{13}C NMR and ESI-HRMS spectra of target compound ***12b***

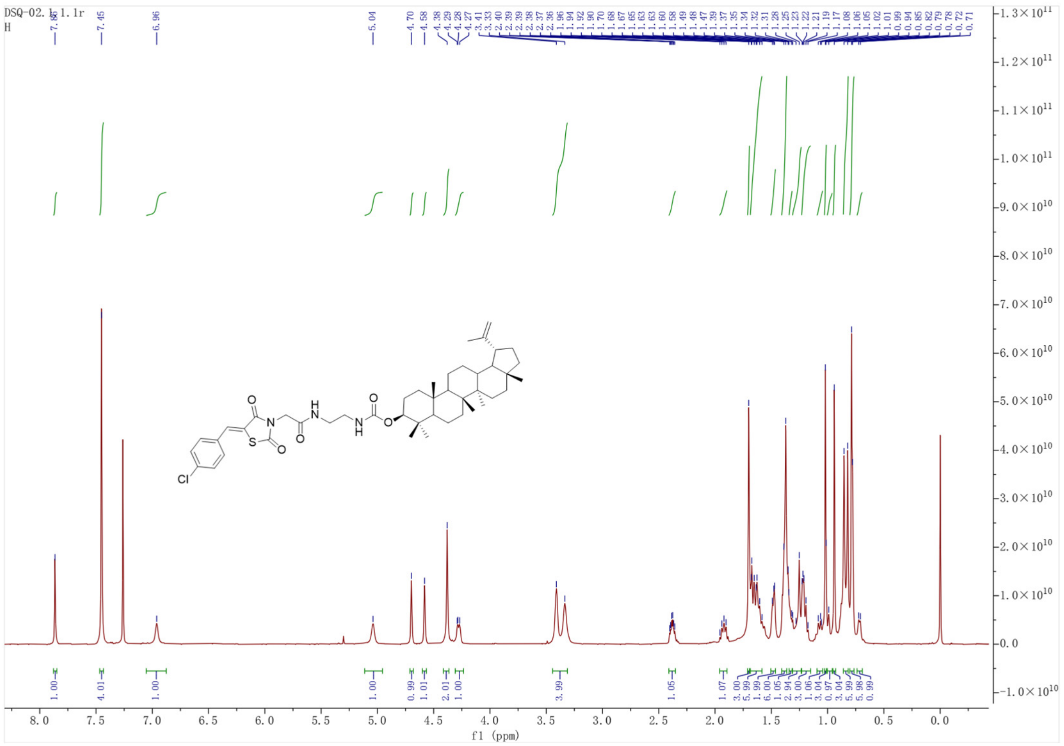

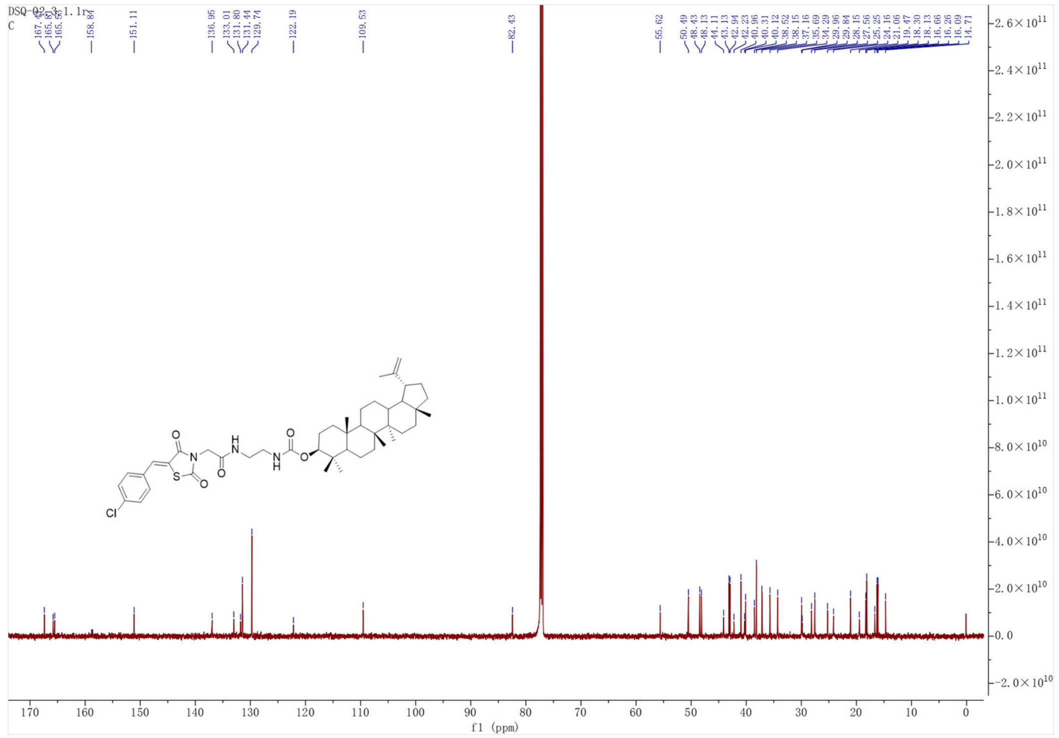

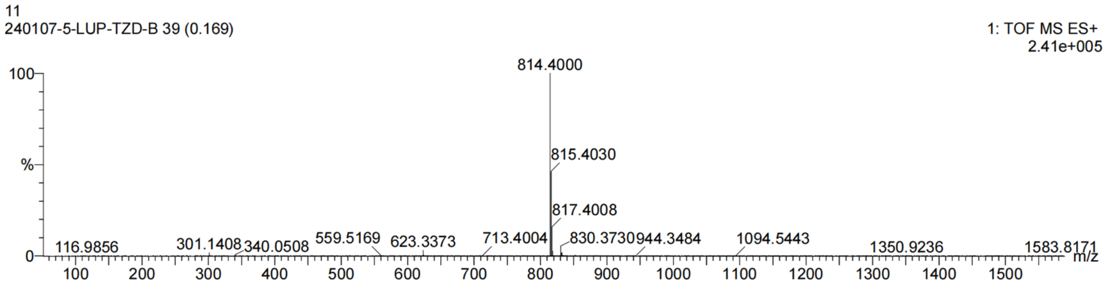

^{1}H NMR, ^{13}C NMR and ESI-HRMS spectra of target compound **12c**

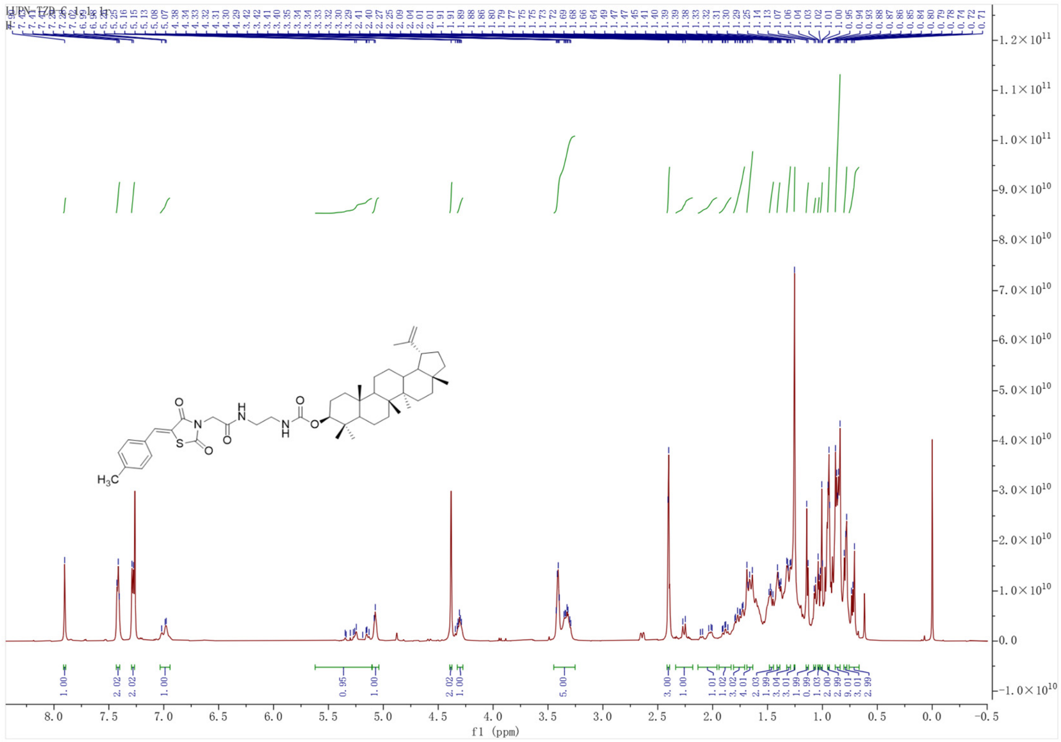

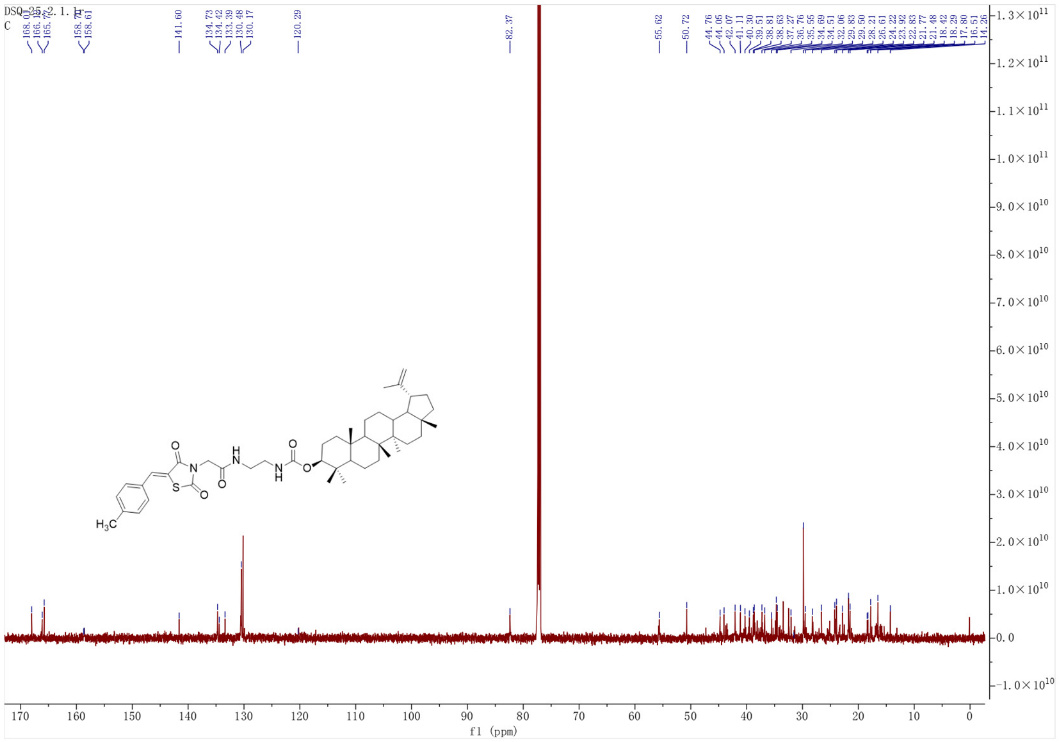

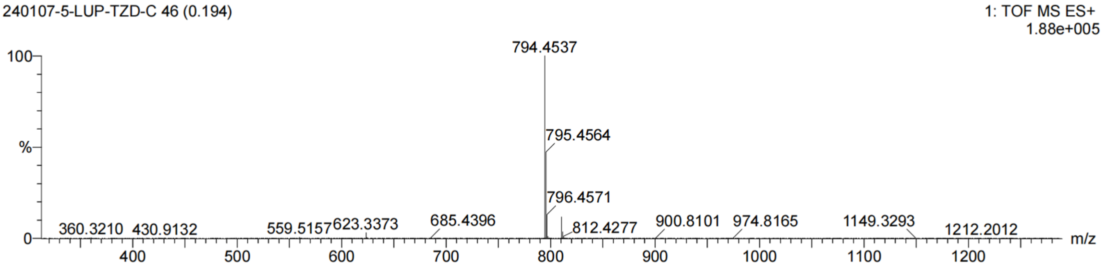

*^{1}H NMR, ^{13}C NMR and ESI-HRMS spectra of target compound **12d***

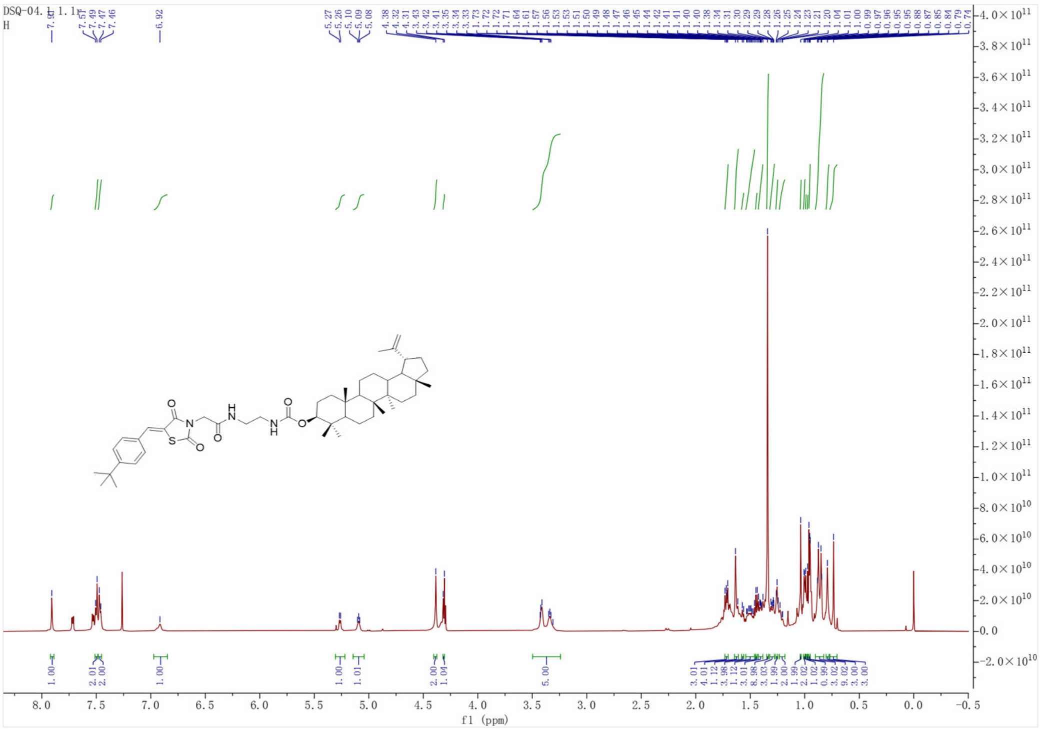

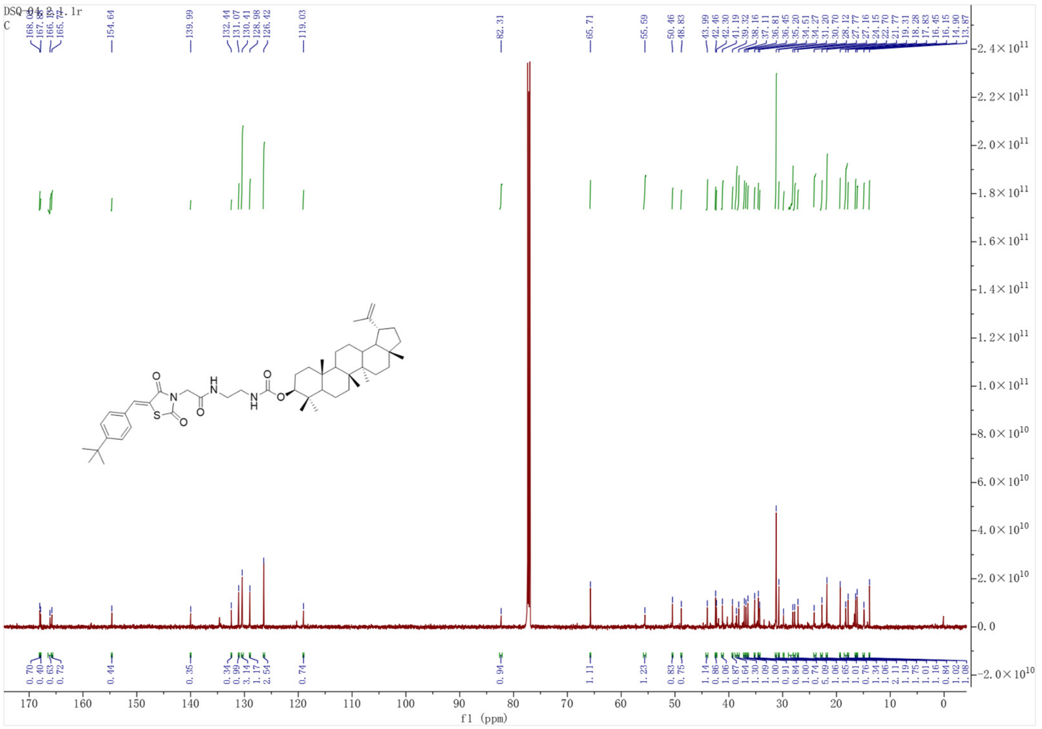

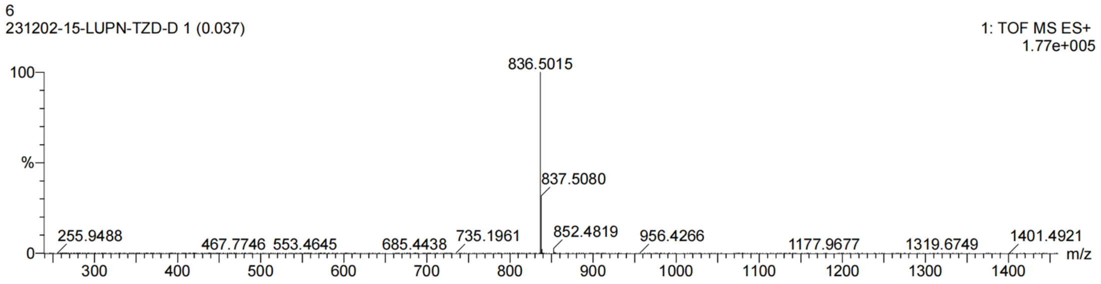

^{1}H NMR, ^{13}C NMR and ESI-HRMS spectra of target compound ***12e***

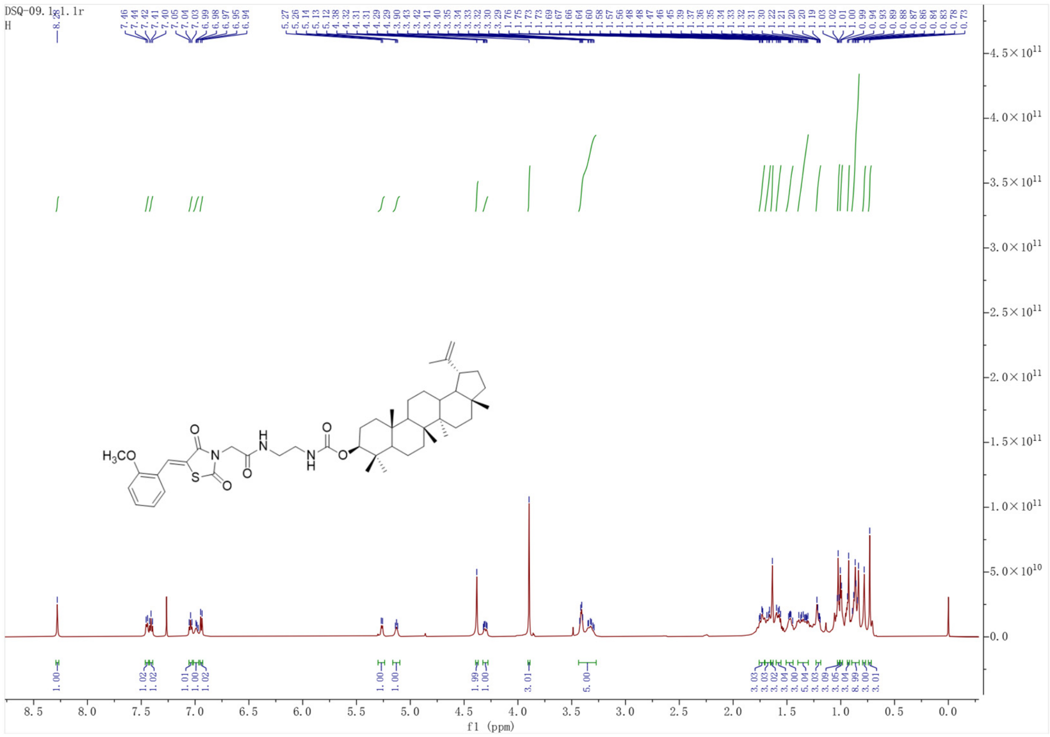

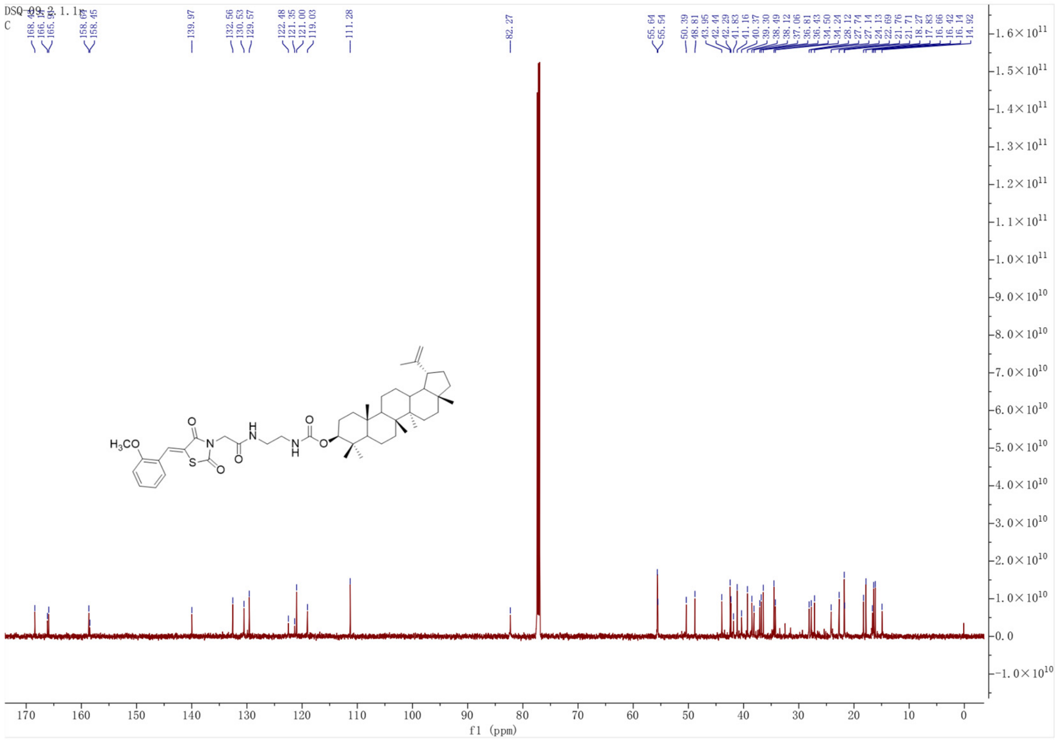

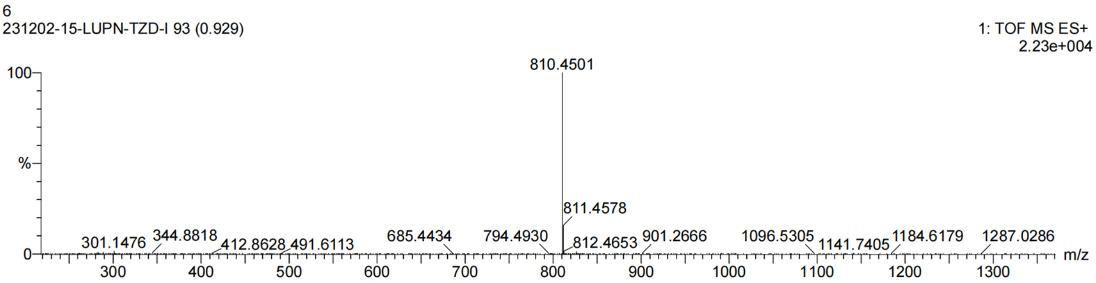

^{1}H NMR, ^{13}C NMR and ESI-HRMS spectra of target compound ***12f***

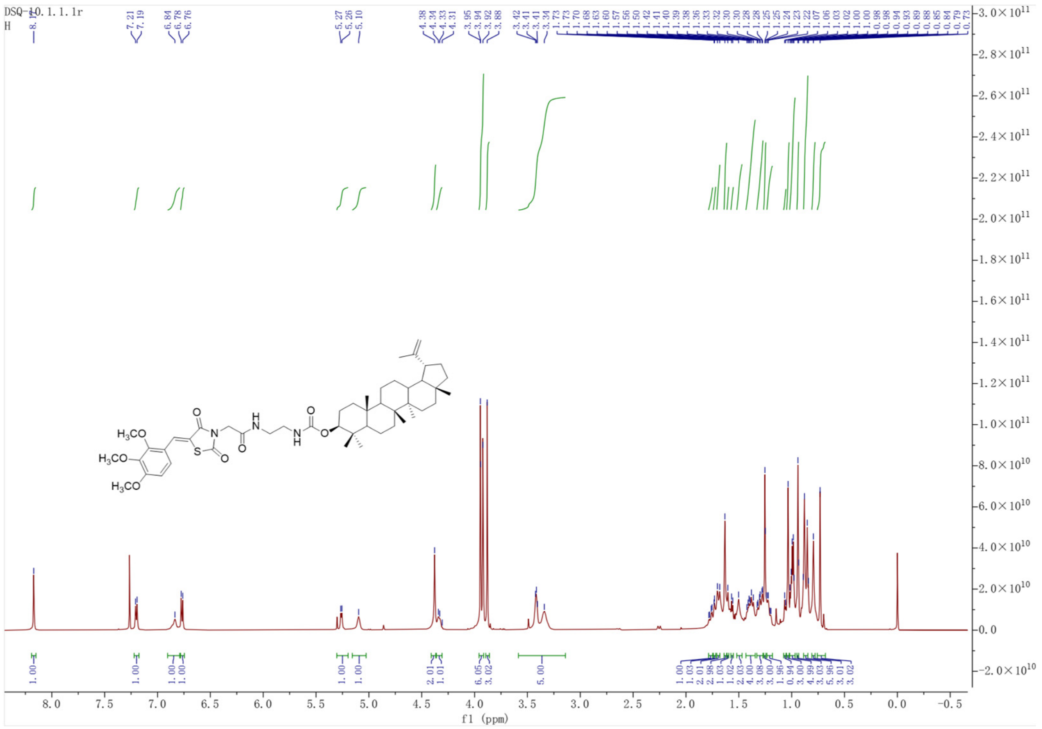

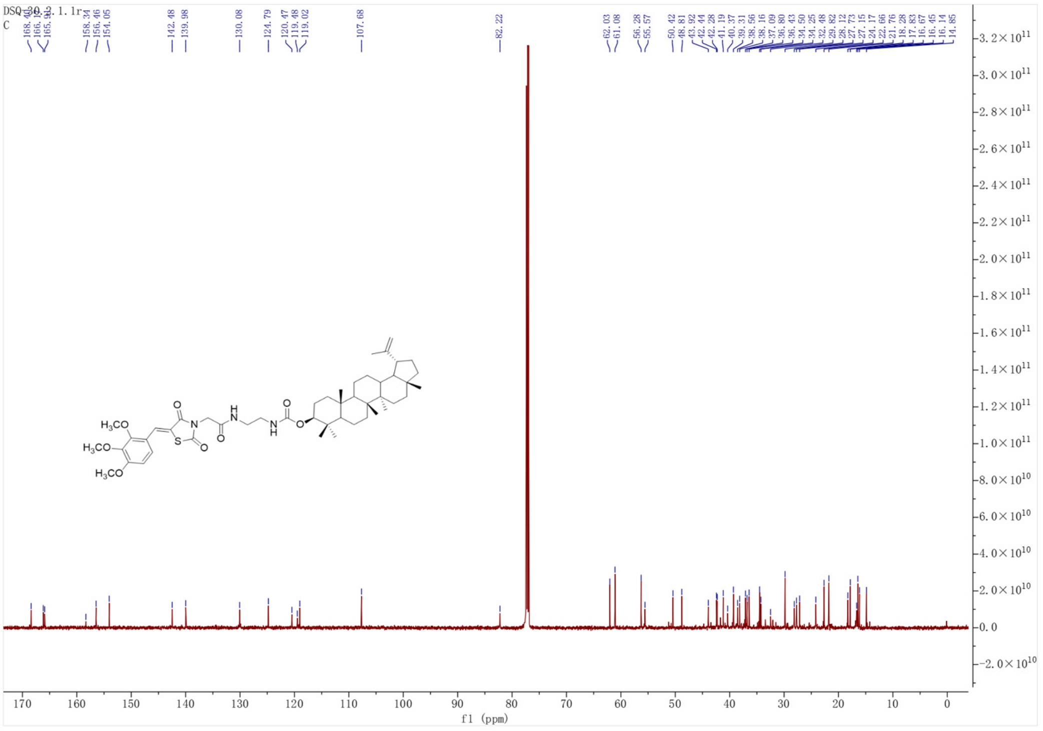

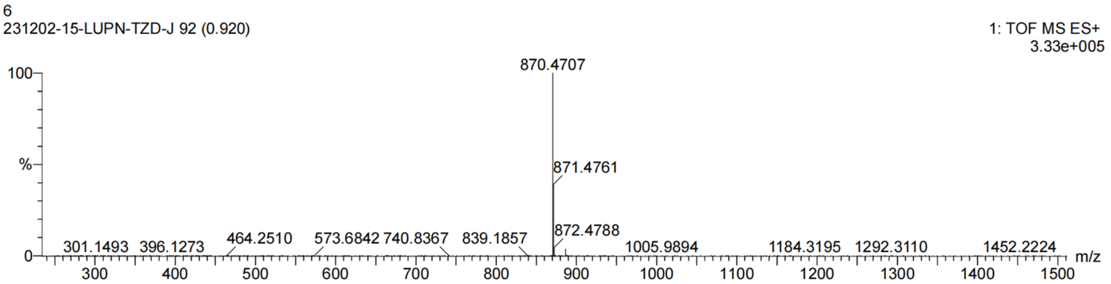

^{1}H NMR, ^{13}C NMR and ESI-HRMS spectra of target compound ***12g***

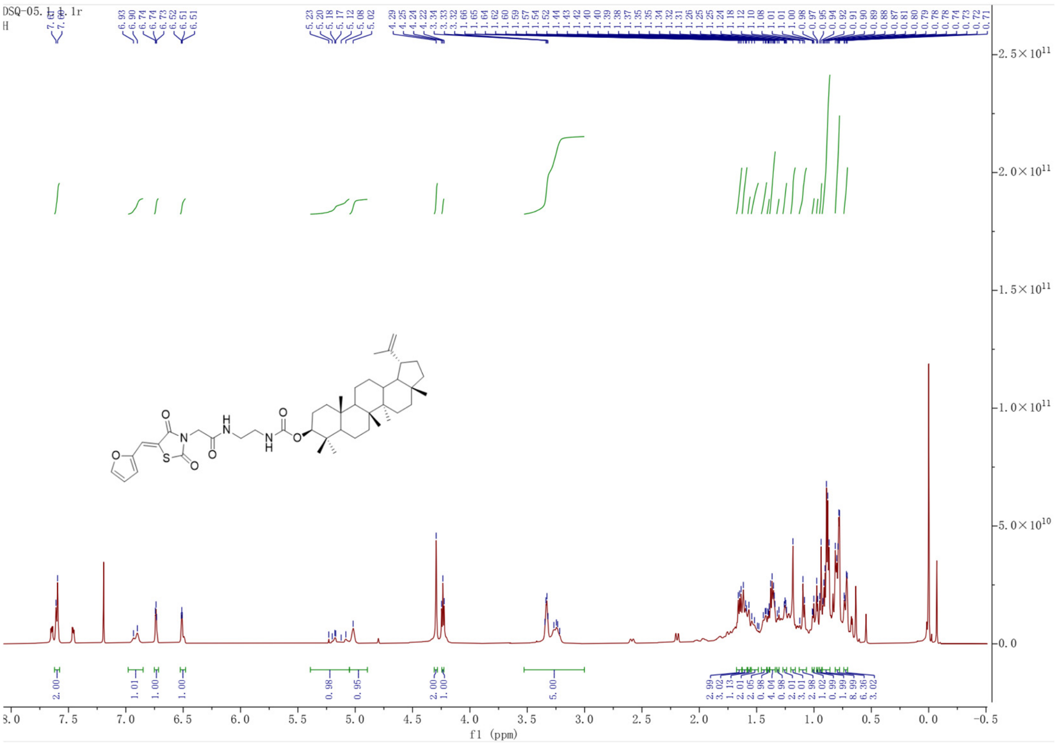

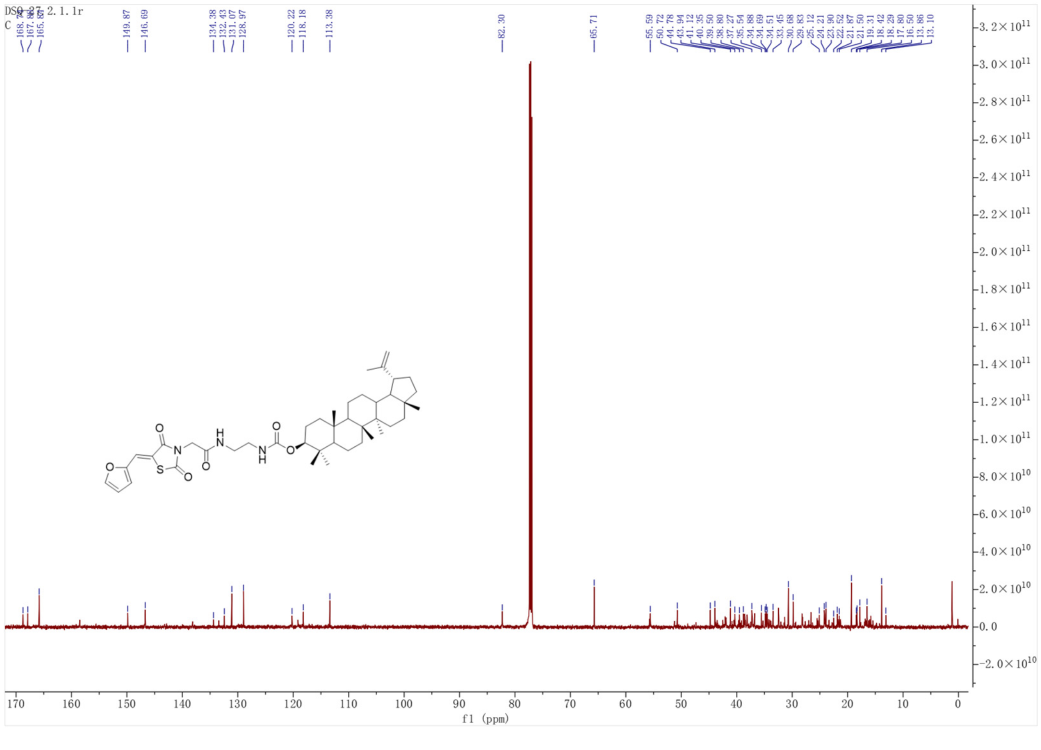

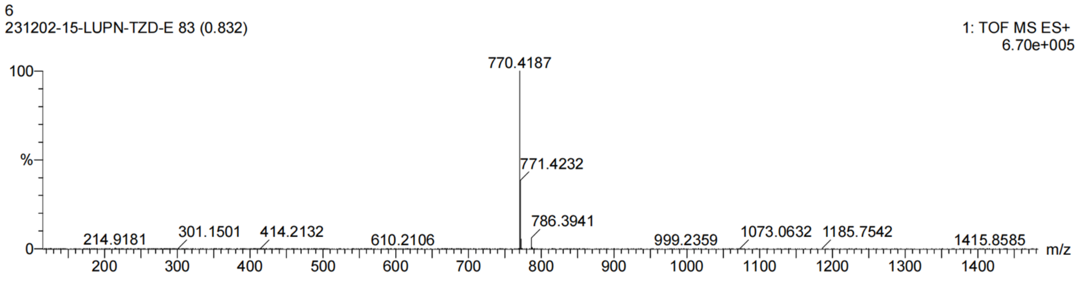

^{1}H NMR, ^{13}C NMR and ESI-HRMS spectra of target compound ***12h***

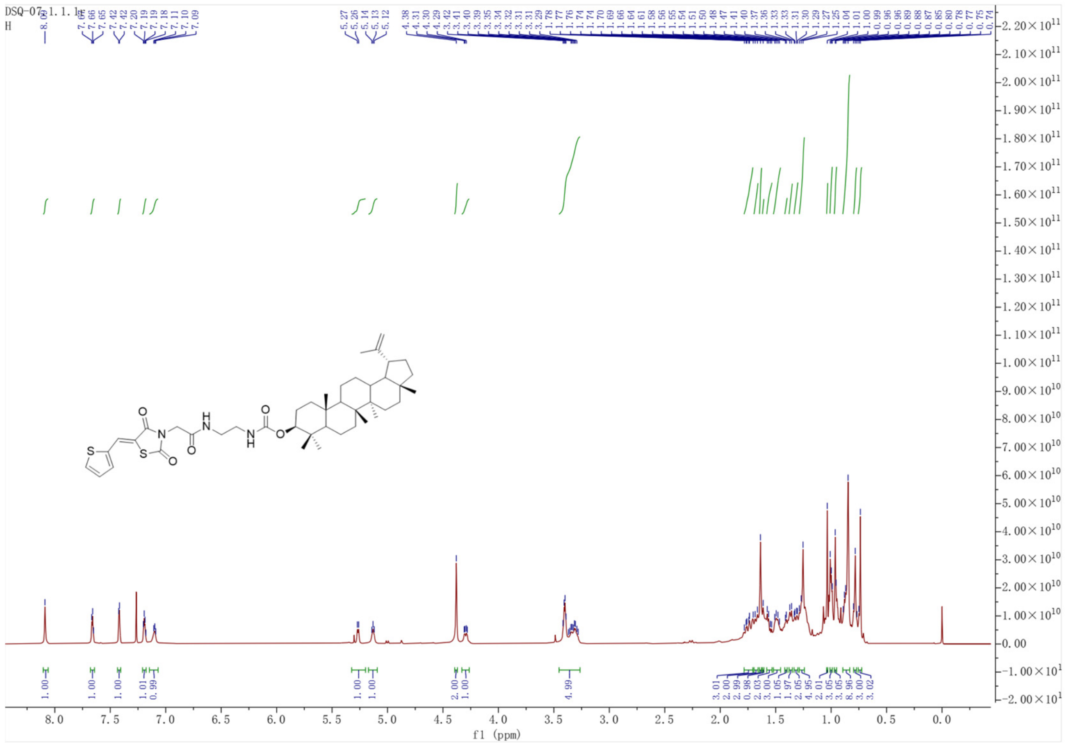

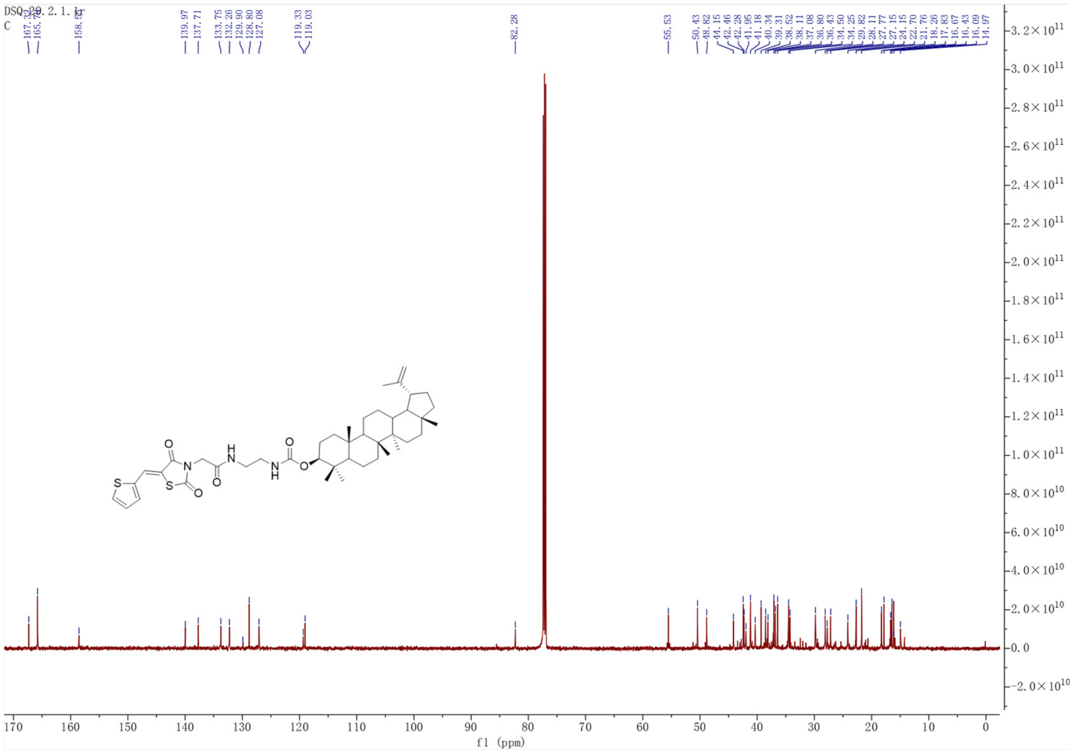

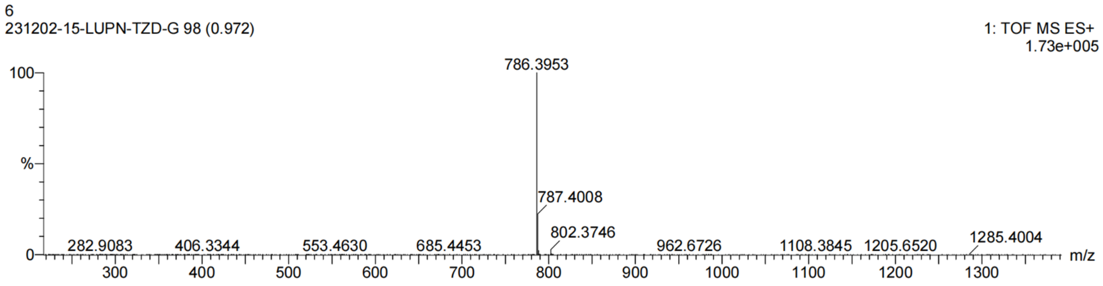

^{1}H NMR, ^{13}C NMR and ESI-HRMS spectra of target compound ***12i***

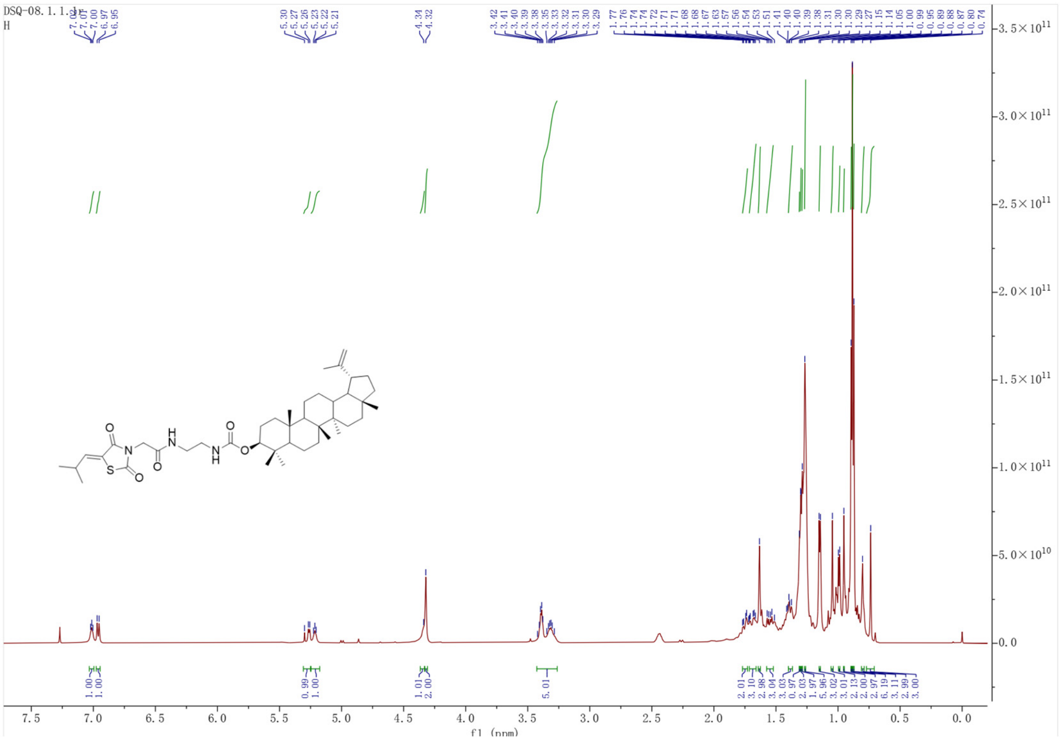

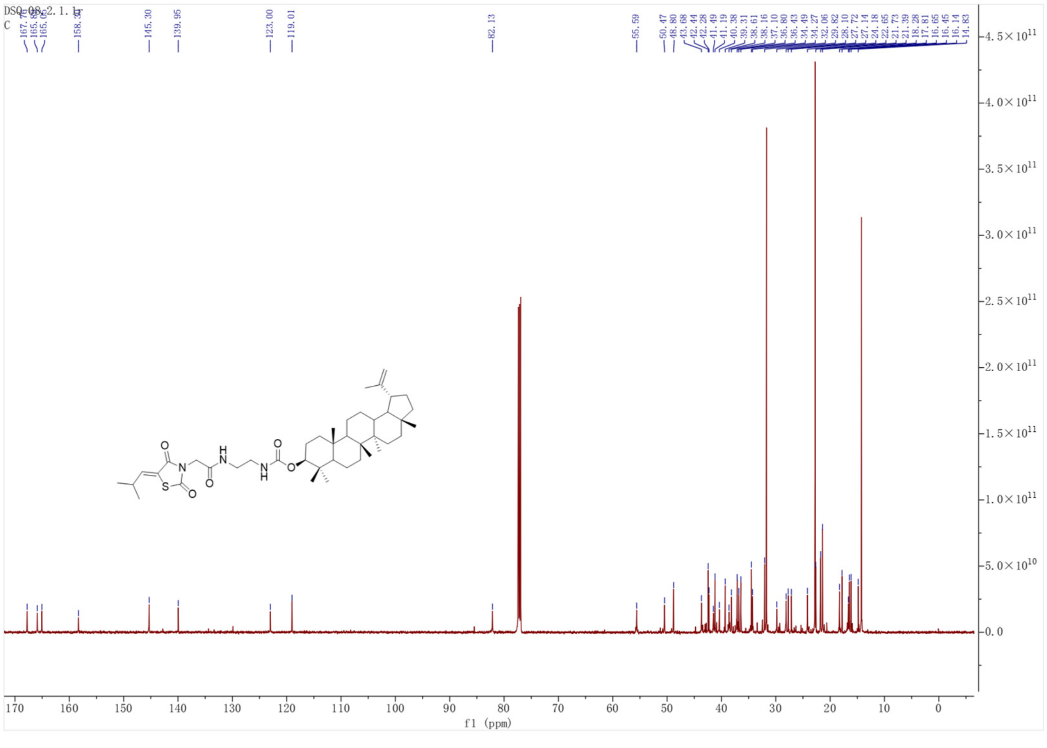

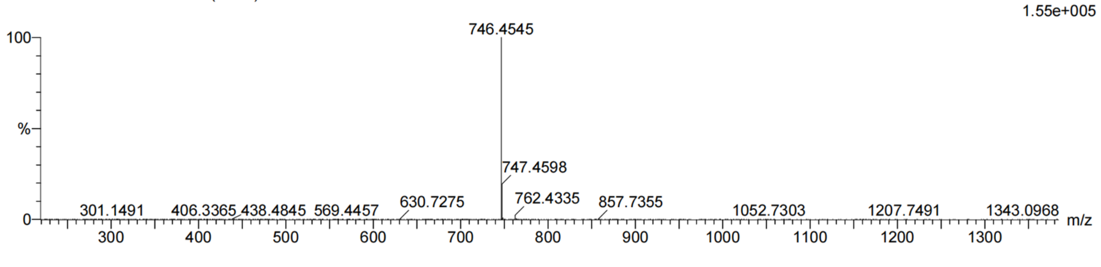

Supplement: Supplementary file 1 [file molecules-29-04957-s001.zip › molecules-3209625-SI.pdf]
